# A Triple-Layer HFM–LFM–CAZAC Preamble Framework for Underwater Acoustic Integrated Sensing and Communication

**DOI:** 10.3390/s26154814

**Published:** 2026-07-29

**Authors:** Seunggyu Kim, Saeyong Park, Taeho Im

**Affiliations:** Division of Information and Communication Engineering, Hoseo University, Asan 31499, Republic of Korea; ksg980920@naver.com (S.K.); parksaeyong98@gmail.com (S.P.)

**Keywords:** cell identification, constant-amplitude zero-auto-correlation (CAZAC) sequence, hyperbolic frequency modulation (HFM), integrated sensing and communication (ISAC), linear frequency modulation (LFM), low probability of intercept (LPI), matched filter detection, preamble design, sonar signal processing, underwater acoustic communication

## Abstract

We propose a three-functional-layer decomposition framework for underwater acoustic (UWA) integrated sensing and communication (ISAC) preambles, instantiated as P5. Two spectrally separated chirp layers—hyperbolic frequency modulation (HFM) for wideband Doppler invariance and linear frequency modulation (LFM) for sub-meter ranging—are carried under a common constant-amplitude zero-autocorrelation (CAZAC) envelope that supplies cell identification and despreading against a root-blind attacker. Closed-form screening conditions constrain the layers to a near-orthogonal subspace, and direct cross-ambiguity measurement confirms the realized separation. In matched-filter Monte Carlo simulation, P5 meets the continuous-sensing target (range root mean square error $\sigma_{R}\leq 1$ m at 10 dB signal-to-noise ratio) and has the smallest normalized matched-filter peak loss across twelve modeled UWA environments among four tested waveforms. Against four classical structure-aware attackers it stays below the strict $P_{d}\leq 0.1$ low-probability-of-intercept target at 0 dB attacker-input SNR. A 10-seed, 11.2-million-parameter spectrogram ResNet-18 reaches $P_{d}=0.5$ against P5 at mean +21.24 dB total-energy SNR (95% CI [+21.06,+21.42] dB) and $P_{d}=0.1$ at +18.72 dB ([+18.45,+19.00] dB); these crossings are lower bounds on adversary capability, not a security guarantee. The integration also has explicit costs: composite peak-sidelobe level (−7.60 dB default, −11.29 dB optimized) remains inferior to equal-aperture single-waveform baselines, and sixteen-cell identification falls to ≤0.07 under a +2 dB near–far interferer. All-60-sounding WATERMARK replay further gives adverse P5^def^–B5 losses of −0.816 dB on NOF1 (sounding-cluster 95% CI [−1.021,−0.621] dB) and −0.950 dB on NCS1 ([−0.977,−0.923] dB) after all waveforms are scaled into the same measured 8-kHz band. The evidence is therefore simulation dominant and supplemented by measured-channel replay of band-scaled variants; native-band transducer, pool, and sea-trial validation remain future work.

## 1. Introduction

Underwater acoustic (UWA) communication [1,2] has evolved from pure synchronization preambles [3] toward multifunctional designs that must simultaneously support wideband Doppler tracking (R1), sub-sample timing synchronization (R3), cell identification (CID; requirement R2), integrated sensing in the spirit of joint radar-communication, i.e., integrated sensing and communication (ISAC; R4), and low-probability-of-intercept (LPI; requirement D5) operation. Representative UWA mission profiles, distributed autonomous underwater vehicle (AUV) swarms, submarine-to-unmanned underwater vehicle (UUV) low-observability telemetry, ship-to-UUV long-range telemetry, and mine-hunting sonar each stress a different subset of these requirements, and a single deployable preamble family must address all five without requiring mission-specific waveform redesign.

The underwater acoustic channel constrains multifunctional waveform design on every axis. Coherence times range from ∼24 s in calm shallow water to ∼0.08 s in rough seas [4]; multipath delay spreads of 5–50 ms [5] coexist with platform-induced Doppler scales α=(c−v)/(c+v) that cannot be treated as narrowband shifts; and the bounded 4–24 kHz operational band constrains the achievable time-bandwidth product relative to radio-frequency systems by three orders of magnitude. Any preamble that claims to satisfy all five functional requirements must therefore prove orthogonality between sensing and communication components, quantify the Cramér–Rao lower bound (CRLB) reachable within realistic signal-to-noise ratio (SNR) operating points, and demonstrate resistance against the published attacker taxonomy (energy, cyclostationary, cepstral, and machine learning (ML)-based detectors [6,7,8,9,10,11]).

Despite more than four decades of work on individual aspects of this problem [12], existing UWA preamble designs each solve a subset of the five requirements: single-chirp hyperbolic frequency-modulation (HFM) waveforms achieve strong R1 but lack CID and LPI mechanisms; linear frequency-modulation (LFM) plus Zadoff–Chu (ZC) combinations provide sharp ranging but degrade under significant Doppler; phase-coded and superimposed LFM families [13] offer channel-estimation advantages but no explicit LPI performance bound. The recent stretched-arc HFM (SA-HFM) family [14], which serves as our reference baseline B5, still lacks both an integrated ranging layer and a detector-class LPI characterization across the six-attacker taxonomy considered in this paper.

In parallel, integrated sensing and communication—a major theme of recent radio-frequency waveform design—has been applied to UWA contexts over the last two years. Recent UWA-ISAC designs include multiplicative composition [15] (GSFM × GMSK with blind source separation at the receiver), superimposed structures [13], GSFM-based waveforms with multiple-access interference suppression [16], integrated CPM–LFM waveforms for joint communication and detection [17], and an experimental shallow-water UWA-ISAC demonstration [18]. None of these decompose ISAC functions across orthogonal waveform layers, and most retain receiver-side separation (blind source separation, joint demodulation/detection) that is difficult in time-varying UWA channels.

Beyond the acoustic modality, underwater ISAC is also emerging over light waves and magneto-inductive (MI) fields: lightwave power-transfer-enabled underwater optical ISAC has been analyzed under ship-attitude variation [19], and MI-ISAC has been proposed for reactive near-field operation in RF-denied environments [20]. These modalities offer complementary trade-offs—optical links provide high bandwidth over short, alignment-sensitive ranges, while MI links provide stable near-field coupling largely insensitive to multipath—but the acoustic band remains the only carrier supporting the kilometer-scale operational ranges of the mission profiles above, and it is therefore the scope of this paper. Separately, in the radio-frequency ISAC literature, learning-assisted design (deep unfolding, end-to-end autoencoders) is an increasingly common alternative to model-based waveform construction [21]; we position our deterministic design-time approach relative to this direction in Section 7.

The gap we address is therefore specific rather than a claim of absolute novelty: to our knowledge, no existing UWA preamble, single-function baseline or recent multifunction/ISAC proposal, jointly satisfies all five functional requirements (R1 Doppler tracking, R2 cell identification, R3 synchronization, R4 ISAC ranging, and D5 LPI) within one matched-filter-recoverable waveform; each of the designs above closes a subset of the requirements, and most add receiver-side separation complexity. This paper addresses that gap with a layered preamble whose functions are separable by construction rather than by receiver processing. The novelty is not in the three primitives—each individually classical—but in (i) their decomposition into orthogonal functional layers integrated within one matched-filter-recoverable, constant-modulus preamble and (ii) the finding that layer orthogonality is necessary but not sufficient for low composite peak-sidelobe level. The closed-form conditions $C_{⊥}$ serve as a design-time screening tool for admissible layer combinations, with the realized separation confirmed by direct cross-ambiguity measurement.

### 1.1. Contributions

This paper makes the following contributions:

1. A three-functional-layer decomposition framework for UWA ISAC preambles, mapping the five functional requirements (Doppler, synchronization, ranging, CID, LPI) onto three functional layers (HFM, LFM, CAZAC). Two spectrally separated chirp layers combine additively under a common CAZAC phase-spreading envelope; within the considered primitive class (chirp and polyphase-sequence waveforms) these three functional layers address all five requirements.
2. A concrete instantiation, *P5*, a triple-layer composite preamble (see (3)) with formally characterized orthogonality conditions $C_{⊥}$ and three structural properties (Propositions 1–3): layer orthogonality (proven for the smooth-envelope idealization, with the realized chip-modulated suppression characterized numerically), HFM Doppler-invariance preservation (for the ideal HFM phase), and LFM range resolution.
3. Theoretical analysis: 2D wideband ambiguity function (WBAF), a bandwidth-scaling (CRLB-style) reference for delay/Doppler/range, and LPI processing-gain analysis under six non-cooperative attacker models (energy, cyclic-feature, cyclic-spectrum, cepstrum, squaring-frequency-doubling, and blind learned classifiers—a compact 1-D convolutional neural network (CNN) and a 2-D spectrogram ResNet-18) complemented by a detector-class Kullback–Leibler lower bound on miss probability for Neyman–Pearson attackers [22] (valid under the stated observation and noise assumptions of Section 4.6; this is not a claim of unconditional attacker-family agnosticity).
4. Quantitative evaluation over baselines B1–B5 and P1–P4 via Monte Carlo simulation with Bellhop, Qarabaqi–Stojanovic, and WATERMARK channels [23]. Re-verifying every candidate through the Qarabaqi–Stojanovic fine-multipath channel gives a multipath-limited matched-filter range RMSE $\sigma_{R}\approx 0.9$ m at 10 dB SNR—common to all wideband chirp candidates and satisfying R4a (≤1.0 m); the mission-critical R4b (≤0.5 m) is reached in benign short-range geometry (M4 mine-ISAC, AUV docking). Single-target range precision is therefore not a P5-specific differentiator, but P5 still delivers finer two-target resolution through its wider chosen aperture—an allocation advantage, since at equalized aperture, the SA-HFM baselines match the mainlobe and attain lower PSL (Section 7.3). On D5, P5 meets the strict $P_{d}\leq 0.1$ target against all four classical structure-aware attackers and has the highest observed classical breach SNR among the candidates, although its margin over the most resistant baseline is not resolved by the sweep; a trained CNN attacker defeats all chirp-based preambles alike, a limitation of the chirp class rather than of P5 specifically; these D5 results address *blind* attackers, with a root-bank GLRT or full-template adversary—for which the root-randomization margin largely collapses—left out of scope (Section 4.6 and Section 7.3). P5 attains the highest aggregate (R1–R4, D5) score under the stated weighting, reported only as an auxiliary indicator alongside the per-column metrics of Section 5.9.
5. Multi-channel simulation validation across twelve UWA environments (five Bellhop geometric presets, three Qarabaqi–Stojanovic statistical fading regimes, and four WATERMARK sea-trial surrogate channels [23]), providing systematic simulation-based evidence of channel-diversity robustness across geometric, statistical, and measured-surrogate channel models.
6. An empirical observation that layer orthogonality is necessary but not sufficient for low composite autocorrelation peak-sidelobe level (PSL), validated by direct cross-ambiguity measurement (layer cross-term peaks −33 to −42 dB, all below the −20 dB threshold, yet composite PSL bounded at −7.60 dB untapered). This separation motivates treating layer orthogonality and PSL as distinct deliverables addressed by disjoint design tools.
7. Mission-profile reconfigurability demonstrated across four UWA operational scenarios (M1 AUV swarm, M2 submarine-UUV, M3 ship-UUV, M4 mine-ISAC) via parameter-only retuning (N,ρ,r) without structural change.

The remainder of this paper is organized as follows. Section 2 presents the UWA channel model, quantitative requirements R1–R4 and D5, and the baseline and recent-proposal landscape that motivates the gap. Section 3 introduces the three-functional-layer decomposition framework and the orthogonality region $C_{⊥}$. Section 4 formalizes the P5 waveform with signal definition, WBAF analysis, CRLB bounds, and LPI processing-gain analysis (Propositions 1–3). Section 5 presents Monte Carlo simulation results across the three channel models, including the weighted aggregate comparison and six-attacker LPI ablation. Section 6 reports multi-channel simulation validation across twelve UWA environments spanning Bellhop geometric, Qarabaqi–Stojanovic statistical, and WATERMARK measured-surrogate channel families. Section 7 addresses limitations, extensions, and implementation considerations. Section 8 concludes.

### 1.2. Scope Statement

This is a simulation-based framework-and-analysis paper: the contribution is the three-functional-layer decomposition, the closed-form orthogonality/Doppler/range analysis (Propositions 1–3), and a characterization across geometric, statistical, and measured-surrogate channels, complemented in revision by a band-scaled replay experiment on measured impulse responses (Section 6.5). Native-band sea-trial measurements and a full-knowledge adversarial LPI evaluation are out of scope and remain future work. All quantitative and superiority claims (R4, D5) are empirical observations within the stated channel and attacker assumptions, not unconditional guarantees.

## 2. System Model and Requirements

### 2.1. UWA Channel Model

The received signal under a wideband time-varying UWA channel is modeled as a superposition of scaled, delayed, and amplitude-weighted copies of the transmitted waveform [24]:

(1) $$r\left(t\right)=\sum_{m=M_{0}}^{M_{1}}\sum_{n=0}^{N(m)}\chi_{n,m}\;\alpha_{d}^{m/2}\;s\;\left(\alpha_{d}^{m}t-\frac{n}{W}\right)+w\left(t\right),$$

where $\chi_{n,m}$ is the wideband spreading function (WSF) sample for the (n,m)-th time-shift–scale bin, $\alpha_{d}=\exp\left(1/\beta_{d}\right)$ is the discrete Doppler scale base, *W* is the signal bandwidth, and w(t) is additive noise. This formulation, originally from Weiss and refined by Jiang and Papandreou-Suppappola, generalizes the classical narrowband delay-Doppler model in two ways: (i) Doppler enters as a time-scale compression rather than a frequency shift, and (ii) the channel decomposes into $M=\sum_{m}\left(N\left(m\right)+1\right)$ independent flat-fading subchannels, enabling joint multipath-scale diversity exploitation.

The UWA channel exhibits four statistical regimes relevant to preamble design, characterized by Yang [4]: *temporal coherence* Γ(τ), *spatial coherence length* $\rho_{0.8}$, *per-path Rician amplitude statistics* $\mu^{2}/2\sigma^{2}$, and *channel randomness* $\varepsilon^{2}$. For the M1 AUV swarm scenario (calm shallow water, comparable to AUVFest07), we observe $\tau_{0.8}\approx 24$ s and $\varepsilon^{2}\leq -16$ dB, permitting sparse-channel equalization. For the M2 submarine-UUV scenario (dynamic ocean, comparable to TREX04), $\tau_{0.8}$ drops to ∼0.17 s and $\varepsilon^{2}\approx -6.5$ dB, mandating turbo-equalization. We use three complementary channel simulation backends (Bellhop ray-tracing [25], the Qarabaqi–Stojanovic statistical channel [26], and the WATERMARK measured-data benchmark [23]) to cross-validate preamble performance across regimes.

### 2.2. UWA Operational Requirements

Table 1 summarizes the five functional requirements R1–R4 and D5 (six sub-requirements once R4 is split into R4a/R4b) with quantitative targets derived from the sponsor program specification [14] and standard UWA operational envelopes [8,27]. The weighting column is used to score aggregate waveform performance in the weighted multi-requirement evaluation (Section 5).

R4 is split into two tiers to reflect the two operational regimes of UWA ISAC: R4a covers the continuous sensing baseline adequate for navigation-grade ranging and multi-target discrimination in M1–M3, and R4b covers high-precision (mission-critical) ranging required for M4 mine-ISAC and short-range AUV docking. Together, R4a and R4b carry the same 30% aggregate weight as the single-tier R4 of earlier formulations. R1 retains high weight because Doppler-compensation failure cascades into R2/R3 degradation. D5 weight is deliberately modest because, in the operational context considered, LPI is typically a *constraint satisfaction* condition (met or not met) rather than a continuous optimization target [12,22].

### 2.3. Prior Baselines (B1–B5)

We benchmark against five baselines spanning the published UWA preamble landscape.

B1 (HFM-single): One HFM down-sweep over $\left[f_{1},f_{2}\right]$. Excellent R1 (Doppler-invariance) [28]; no R2/R4/D5.
B2 (Dual HFM): Paired up+down HFM sweep. Enables Doppler-sign disambiguation but still lacks R2/R4.
B3 (LFM+ZC): Single LFM chirp modulated by a ZC envelope. Good R3/R4 but degrades under moderate Doppler; ZC provides modest D5 gain.
B4 (Superimposed LFM): *K* overlapping LFM chirps with distinct chirp rates [13], developed for OFDM-style channel estimation. No D5 analysis in the original work.
B5 (SA-HFM): Superposed Adjusted HFM from the Park-Chung-Im 2025 lab lineage [14], serving as this work’s reference baseline. Strong R1/R2/R3 via the SA-HFM family’s multifunctional construction, but no explicit ranging layer (R4 is coupled to the HFM mainlobe width, which is wide) and no LPI analysis (D5).

### 2.4. Recent Proposals and Identified Gap

Two concurrent proposals, P3 (HFM+CAZAC) and P4 (ISAC-enhanced SA-HFM), represent the two dominant paths toward closing R4 and D5 gaps. P3 combines the HFM carrier with a CAZAC envelope for LPI-strong operation (exploiting the CAZAC constant-amplitude zero-autocorrelation property [29]) but retains HFM’s wide mainlobe and thus moderate R4. P4 augments SA-HFM with a dedicated sensing branch via power allocation ($\rho_{\mathrm{isac}}$), improving R4 but incurring a narrower sensing bandwidth and no explicit D5 treatment. Neither candidate simultaneously provides R4a-compliant ranging and reduced detectability against the four classical structure-aware attackers. P5 addresses this gap by combining R4a-compliant ranging with D5 LPI that meets the strict target against all four classical structure-aware attackers (CFD, CS, CEP, SFDM); a learned-CNN attacker defeats every chirp-based candidate alike and is reported as a class-wide limitation rather than a P5-specific weakness (Section 4.6).

*Differentiation from closely-related prior art:* The three primitives (HFM, LFM, CAZAC) are individually classical; P5’s contribution is the set of conditions under which their combination is admissible and separable. (a) *Functional-layer separation:* the layers occupy a near-orthogonal subspace $C_{⊥}$ (Section 3.2, Proposition 1), stated as design-time parameter inequalities rather than verified after the fact. (b) *Orthogonality–PSL decoupling:* layer orthogonality is necessary but not sufficient for low composite PSL, motivating two separate design tools (the $C_{⊥}$ inequalities and a Pareto search). (c) *Co-designed LPI and CID:* one constant-modulus CAZAC envelope supplies both the R2 codebook and the D5 spreading over the same chips. None of the works below combine all three under one matched-filter-recoverable, constant-modulus CAZAC-envelope preamble.

Three recent works bear direct comparison. Tan et al. 2019 [9] analyze ZC sequences under UWA Doppler via cyclic-autocorrelation detection; we adopt their CFD as a transmit-side anti-detection benchmark (Section 4.6). Zuberi et al. 2022 [30] use binary-phase-coded HFM with Gold/Kasami sequences, whose −13 dB autocorrelation sidelobes compete with timing; CAZAC instead supplies both the D5 spreading and the R2 codebook with zero autocorrelation. Lv et al. 2024 [13] target data-bearing superimposed-LFM for channel estimation, whereas our preamble-only triple-layer design admits closed-form ambiguity analysis. Niu et al. 2023 [15] use a multiplicative GSFM × GMSK ISAC waveform with receiver-side blind source separation; our additive HFM+LFM-under-CAZAC composition is matched-filter-recoverable and avoids that PAPR and BSS complexity.

*Qualitative comparison map:* Table 2 summarizes the five baselines (B1–B5), four recent proposals (P1–P4), and our proposed P5 against the six functional sub-requirements (R1, R2, R3, R4a, R4b, D5). Three observations from the table drive the analysis: (i) no baseline simultaneously scores Yon R4a *and* D5; (ii) recent proposals close either R4 (P4) or D5 (P3) but not both; (iii) P5 is the only design that integrates Doppler-invariant timing (R1), a dedicated ranging layer (R4a), and a CAZAC envelope for CID (R2) in a single waveform while attaining D5 LPI against the classical structure-aware taxonomy (the strict $P_{d}\leq 0.1$ target is met for all four classical attackers CFD, CS, CEP, SFDM; a learned-CNN attacker defeats all chirp-based candidates and is a class-wide limitation, see Section 4.6 and Section 7.3), with R4b attained in benign short-range (quasi-static) geometry (Section 4.5).
*Legend (Table 2):* Y indicates the waveform meets the quantitative target of Section 2.2; P indicates partial coverage; Cond. denotes a conditional Y (target reached only under specific SNR or *T* regimes, see footnote ‡); N indicates the requirement is not supported. R4 is split into R4a (continuous sensing, $\sigma_{R}\leq 1.0$ m @ 10 dB SNR) and R4b (precision ranging, $\sigma_{R}\leq 0.5$ m, mission-critical). The entry-level conditions for P (partial) on the non-D5 requirements are as follows: R1 means Doppler tracked only for |α−1|≤1%; R2 means <16 unique IDs; R3 means $\sigma_{\tau }\in \left(2,4\right]$ samples @ 96 kHz; R4a means $\sigma_{R}\in \left(1.0,1.5\right]$ m @ 10 dB (design-capability rubric; the realized single-target $\sigma_{R}$ is multipath-limited and uniform, see footnote); R4b means the ≤0.5 m target is reached only in benign short-range (quasi-static) geometry. The D5 ladder follows the same Y/Cond./P/N rule applied to the four classical structure-aware attackers (CFD, CS, CEP, SFDM); the learned-CNN attacker is excluded from per-waveform D5 scoring because it defeats all chirp-based candidates alike (Section 4.6) and is reported separately as a class-wide limitation, with full thresholds tabulated below.

*Identified gap:* Table 2 makes the design problem precise: no existing waveform simultaneously delivers Doppler-invariant timing (R1), sub-meter ranging at 10 dB SNR (R4a), and reduced detectability against the four classical structure-aware detectors (D5). The remainder of this paper addresses this gap by proposing a single waveform structure, P5, combining two spectrally separated chirp layers under a common CAZAC phase-spreading envelope (Section 3) and quantifying the resulting performance against the requirements of Table 1 (Section 4, Section 5 and Section 6).

## 3. Proposed Framework: Three-Functional-Layer Decomposition

**Figure 1:**
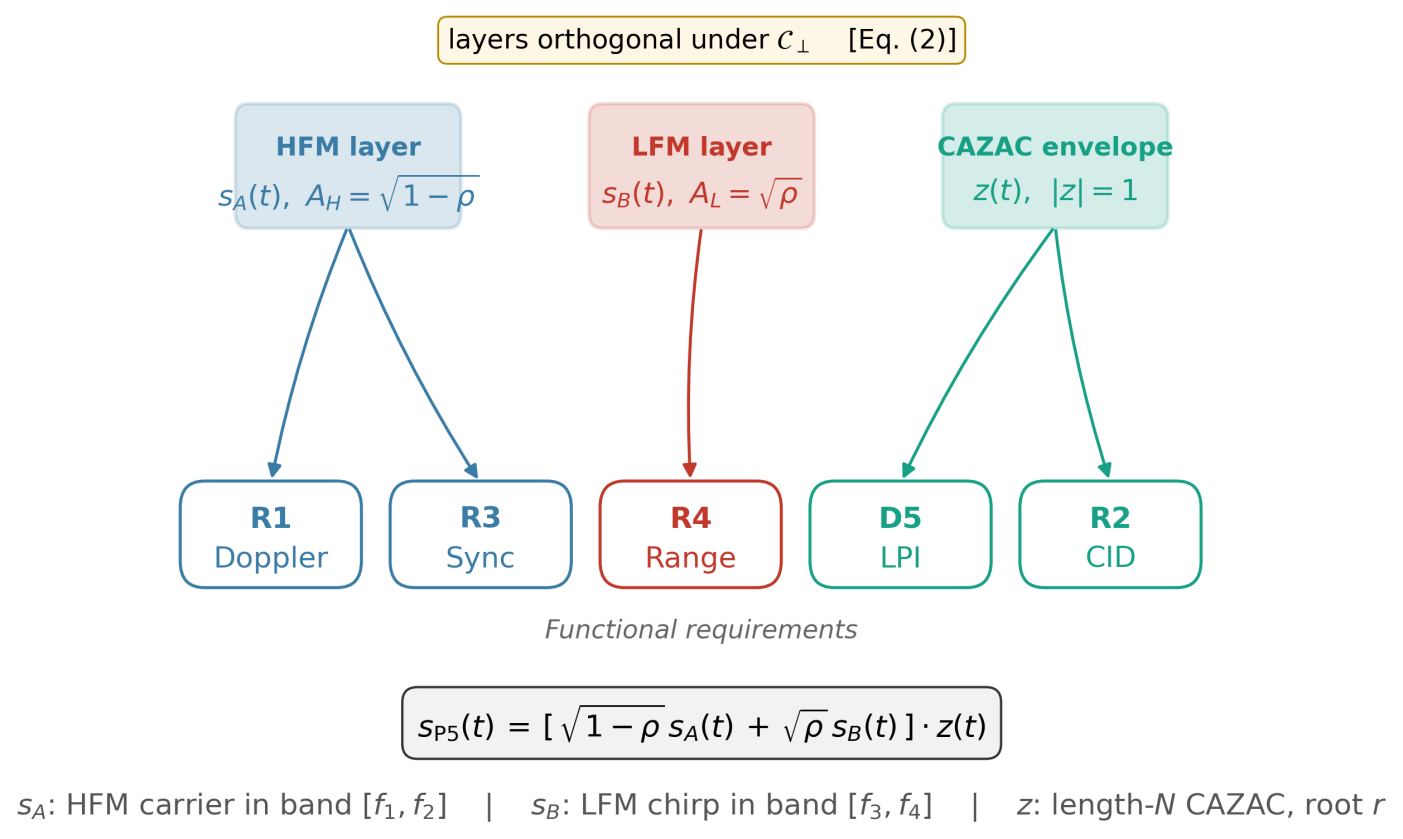
Three-layer decomposition framework, mapping each waveform layer to its primary functional requirement.

We distribute UWA ISAC preamble functionality across three functional layers, each matched to a distinct subset of the R1–R4+D5 requirements. Strictly speaking, the HFM and LFM contributions are additive chirp components, while the CAZAC layer is a multiplicative phase-spreading envelope; we use “three-layer” in the functional-decomposition sense (one HFM functional layer, one LFM functional layer, one CAZAC functional layer), and we distinguish *additive signal orthogonality* between the two chirp layers from the *functional orthogonality* between chirp-layer ambiguity and CAZAC-envelope spreading. Within the class of standard chirp primitives and constant-amplitude polyphase sequences, we show that three functional layers address all five requirements and formalize the orthogonality conditions under which the layer ambiguity functions do not interfere. Figure 1 illustrates the mapping that the remainder of this section formalizes: the HFM layer carries Doppler-invariant timing (R1, R3), the LFM layer provides wideband range resolution (R4), and the CAZAC envelope supplies the constant-amplitude, zero-autocorrelation spreading that delivers both LPI (D5) and cell identification (R2). The two chirp layers combine additively with power-split ρ, and the sum is then modulated by the length-*N* CAZAC envelope z(t), producing the composite expression shown at the bottom of the figure.

### 3.1. Functional Requirements → Layer Assignment

Table 3 summarizes the mapping between the five functional requirements (R1–R4+D5) and the three waveform layers (HFM, LFM, CAZAC). Each requirement is served by exactly one *primary* layer (the layer whose signal structure is most directly aligned with the physical invariant required), though layers may contribute *secondary* support to additional requirements through their envelope or phase structure.

*Observation (Sufficient three-layer structure under standard primitives):* Within the class of preambles built from standard chirp primitives (linear and hyperbolic frequency modulation) and constant-amplitude polyphase sequences, no two-layer combination appears to simultaneously satisfy R1 (Doppler invariance over wideband α-scale), R4 (sharp range resolution via $\Delta \tau =1/B_{L}$), and D5 (LPI processing gain independent of carrier frequency) without additional structural elements.

*Argument.* Doppler-invariance requires the HFM phase structure $\Phi \left(\alpha t\right)=\Phi \left(t-\tau_{D}\left(\alpha \right)\right)+\psi_{0}\left(\alpha \right)$ (Proposition 2), which is a property of hyperbolic (but *not* linear) chirps. Conversely, range resolution $\Delta \tau =1/B_{L}$ is attained when the instantaneous frequency sweeps linearly (LFM), producing a rectangular-spectrum-like ambiguity mainlobe; hyperbolic chirps yield a non-rectangular spectrum with ∼1.5× wider mainlobe at equal bandwidth. The two functional demands therefore *benefit from two distinct chirp types* within the class considered. D5 is not achievable by chirp-only signaling within this class (bounded time–bandwidth gain ≤$10\log_{10}\left(BT\right)$); the root-keyed CAZAC code adds a constant-modulus despreading advantage (∼$10\log_{10}\left(N\right)$ over a root-blind attacker) with |z(t)|=1, which preserves PAPR and does not interfere with the chirp-band allocations. Four-layer extensions (e.g., adding a second CAZAC or an FSK envelope) provide no new functional coverage within the class and may increase cross-term interference.

*Scope.* This is a sufficiency statement within the chirp + polyphase-sequence class, not a formal impossibility theorem: generalized primitives (GSFM, Costas codes, nonlinear FM with joint phase coding, multitone CAZAC) may cover the same functions in fewer layers. In particular, the equal-aperture control (Section 5.2) shows that single-target range *resolution* (R4a) is set by the occupied spectral aperture, not by the LFM layer specifically, so a wide-aperture two-layer HFM–CAZAC (P3 re-allocated to 8–22 kHz) can also meet R4a; the LFM layer’s distinct value is the cleaner composite sidelobe structure (lower PSL, better two-target separation, Section 5.6). The three-layer claim therefore concerns the *joint* satisfaction of R1, R4, D5, *and* low composite PSL in one matched-filter preamble—an *integration* argument—not exclusive R4a feasibility. We propose HFM–LFM–CAZAC as an effective, closed-form-analyzable, PAPR-preserving architecture within this class; broader minimality claims are out of scope.

### 3.2. Orthogonality Conditions $C_{⊥}$

Layer orthogonality is characterized by a set of parameter inequalities that jointly ensure the cross-ambiguity terms ${\tilde{\chi }}_{HL},{\tilde{\chi }}_{HZ},{\tilde{\chi }}_{LZ}$ remain below a design threshold (typically −20 dB relative to the mainlobe peak). Let $B_{H}=f_{2}-f_{1}$ and $B_{L}=f_{4}-f_{3}$ denote the HFM and LFM bandwidths, $\Delta_{BW}=f_{3}-f_{2}>0$ the inter-band separation, and $TW_{H}=B_{H}T$, $TW_{L}=B_{L}T$ the per-layer time-bandwidth products.

(2) $$C_{⊥}=\left\{\theta |\;\begin{array}{c} 0<f_{1}<f_{2}<f_{3}<f_{4},\\ \Delta_{BW}\geq \Delta_{BW}^{\min},\\ TW_{H},TW_{L}\geq K_{0},\\ NT_{c}=T,\;\gcd\left(r,N\right)=1 \end{array}\right\},$$

where $\theta =(f_{1},f_{2},f_{3},f_{4},T,T_{c},N,r)$. The set (2) is a *screening* condition: it guarantees the smooth-envelope bound of Proposition 1 (the $C_{⊥}^{\mathrm{ideal}}$ regime), whereas the realized suppression of the chip-modulated envelope—which depends additionally on the chip rate $1/T_{c}=N/T$ relative to $\Delta_{BW}$, a dependence not captured by (2)—is verified numerically below.

With $\Delta_{BW}^{\min}=1$ kHz and $K_{0}=100$, the stationary-phase argument yields the *idealized* cross-term bound $|{\tilde{\chi }}_{HL}\left|/\right|{\tilde{\chi }}_{HH}{|}_{\max}\leq 1/\left(2\pi \Delta_{BW}T\right)$, evaluating to −62 dB at the default parameters ($\Delta_{BW}=2$ kHz, T=100 ms). The non-stationary-phase proof additionally requires the Doppler-dependent gap condition $\Delta_{BW}>\eta f_{3}$ (Supplementary S1); for the R1 Doppler extent η=2%, this reads $\Delta_{BW}>360$ Hz, comfortably satisfied by the 2 kHz default gap. This bound treats the CAZAC kernel $K_{Z}$ as a smooth bounded amplitude; the actual CAZAC envelope is piecewise-constant with N−1 chip discontinuities at chip rate $1/T_{c}=N/T$, each of which leaks energy across the inter-band gap. When $1/T_{c}$ exceeds $\Delta_{BW}$ (e.g., $1/T_{c}=2.55$ kHz vs. $\Delta_{BW}=2$ kHz at the defaults), the leakage partially bridges the gap, so the idealized −62 dB figure is an optimistic upper bound on the smooth component only. We therefore report the realized suppression of the actual envelope-modulated layers below.

*Measured cross-ambiguity (actual envelope):* The *full* delay–Doppler peak of the cross-ambiguity function for the real envelope-modulated layers (zh vs. zℓ, etc.), evaluated over all delays and the full R1 Doppler extent |ν|≤440 Hz (covering the ±2% scale at the 22 kHz LFM edge), is −32.9 dB (HFM-LFM), −35.5 dB (HFM-ZC), and −42.0 dB (LFM-ZC) at the default parameters; extending the search from |ν|≤300 to 440 Hz changes these by <0.2 dB, so the operating Doppler range is fully covered. The two cross-orderings have equal peak magnitude ($|{\tilde{\chi }}_{HL}{|}_{\max}={\left|{\tilde{\chi }}_{LH}\right|}_{\max}=-32.9$ dB), consistent with Proposition 1 bounding both terms of the four-term WBAF. (A *zero-lag* cross-correlation proxy is misleading here because the common |z|=1 envelope cancels at zero lag, masking the chip leakage; the values above are the worst-case over the entire (τ,ν) plane.) All remain below the −20 dB design threshold of $C_{⊥}$ with ≥12 dB of margin. The chip-rate dependence is a usable design lever: the PSL-optimal N=511 (P5^opt^) has a higher chip rate ($1/T_{c}=5.11$ kHz) and therefore a larger realized HFM-LFM cross-term, −28.5 dB—4.4 dB above P5^def^ but still ≥8 dB below the threshold—whereas N=127 tightens it to −36.5 dB, trading processing gain for orthogonality.

### 3.3. Observation: Orthogonality vs. Composite PSL

*Observation.* While the three layers exhibit adequate orthogonality ($\left|{\tilde{\chi }}_{HL}\right|,\left|{\tilde{\chi }}_{HZ}\right|$, $\left|{\tilde{\chi }}_{LZ}\right|\leq -20$ dB over $C_{⊥}$, with measured full delay–Doppler peaks of −33 to −42 dB for the actual envelope-modulated layers), the *composite* autocorrelation peak-sidelobe level (PSL) is bounded by the per-layer chirp sidelobe structure. Specifically, the untapered composite with default parameters exhibits PSL of −7.60 dB due to coherent addition of per-layer chirp sidelobes, which is unaffected (or even worsened) by conventional tapering (Hann, Tukey). This indicates that layer orthogonality is a *necessary but not sufficient* condition for low composite PSL; achieving PSL below −10 dB requires joint parameter and tapering optimization (Section 5.3).

This decoupling is operationally useful: layer orthogonality *unlocks* multifunctional operation (it is the predicate for Propositions 1–3), while PSL reduction is a downstream optimization handled by a separate Pareto search (Section 5.3) that found an optimum of −11.29 dB with (ρ,N,r)=(0.40,511,37).

### 3.4. Design Knobs

Eight parameters span the design space: four frequency edges $\left(f_{1},f_{2},f_{3},f_{4}\right)$, preamble duration *T*, CAZAC length *N* and root *r*, and power split $\rho =A_{L}^{2}/\left(A_{H}^{2}+A_{L}^{2}\right)$. Monte Carlo sensitivity analysis over a 105-point (ρ,N,r) grid (holding $f_{i},\;T$ at defaults) identifies CAZAC length *N* as the dominant knob for PSL and LPI gain (±5 dB PSL, ±6 dB LPI across N∈{127,255,511}), with ρ secondary (±3 dB PSL) and *r* a tertiary fine-tune (±1 dB PSL). The $\left(f_{i},T\right)$ parameters are essentially a spectrum-and-duration allocation choice made once per deployment mission.

This parameter hierarchy enables *mission-profile reconfigurability* (Section 5.7): for the submarine-UUV scenario (M2), a conservative (ρ,N)=(0.4,511) maximizes the CAZAC processing gain (and, as a by-product, lowers composite PSL) via the largest *N*; for mine-hunting, ISAC (M4) (ρ,N)=(0.6,127) prioritizes a lower chip rate at the cost of ∼5 dB in both composite PSL and processing gain. Reconfiguration is parameter only, the waveform structure of (3) and the matched-filter receiver are unchanged across missions.

## 4. P5: Triple-Layer Composite Waveform

### 4.1. Signal Definition

The proposed P5 preamble is defined as

(3) $$\begin{array}{cc} s_{P5}\left(t\right)=e^{j\phi_{ZC}\left(t\right)}[\; & A_{H}\;e^{j2\pi \Phi_{HFM}\left(t\right)}\\ \; & +A_{L}\;e^{j2\pi \Phi_{LFM}\left(t\right)}],\;t\in \left[0,T\right], \end{array}$$

where $\Phi_{HFM}\left(t\right)=\int_{0}^{t}f_{HFM}\left(\tau \right)d\tau$ with hyperbolic instantaneous frequency $f_{HFM}\left(t\right)=1/\left[1/f_{1}+\left(1/f_{2}-1/f_{1}\right)t/T\right]$, $\Phi_{LFM}\left(t\right)=f_{3}t+\left(f_{4}-f_{3}\right)t^{2}/\left(2T\right)$, and $\phi_{ZC}\left(t\right)=-\pi r\;n\left(n+1\right)/N$ with $n=\lfloor t/T_{c}\rfloor$, $T_{c}=T/N$, and gcd(r,N)=1. Throughout the paper, we instantiate the CAZAC envelope by the Zadoff–Chu (ZC) sequence [29]; the ZC subscript on $\phi_{ZC}$ reflects this concrete choice, and the two terms (CAZAC envelope, ZC sequence) are used interchangeably from hereon. The power split is parameterized by ρ∈[0,1] with $A_{H}=\sqrt{1-\rho }$ and $A_{L}=\sqrt{\rho }$.

**Figure 2:**
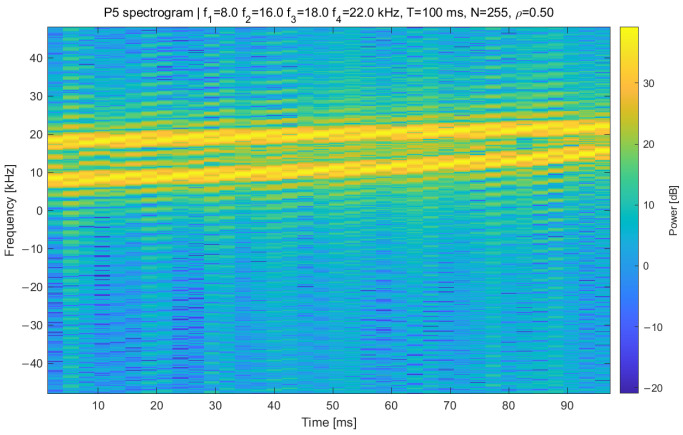
P5 time–frequency structure at default parameters: the spectrogram shows the two spectrally separated HFM and LFM bands carried under the constant-modulus CAZAC envelope.

Observe that $\left|e^{j\phi_{ZC}\left(t\right)}\right|=1$, so the CAZAC envelope is *itself* constant-amplitude and contributes no additional PAPR to the underlying HFM+LFM chirp sum. The composite waveform $s_{P5}\left(t\right)$ is therefore *not* strictly constant-envelope: the two-chirp superposition in the bracket beats between the HFM and LFM instantaneous frequencies, producing a measured PAPR of approximately 3 dB over the tested ρ range (Experiment E-05, Section 5.10). This is still well below the 6–12 dB typical of OFDM-based preambles, and the CAZAC envelope ensures the composite PAPR is determined by the chirp allocation alone and does not scale with *N*. Figure 2 shows the resulting time–frequency structure (spectrogram) for the default parameter set.

*Discrete-time implementation.* At $F_{s}=96$ kHz and T=100 ms, the burst is $F_{s}T=9600$ samples. Since $F_{s}T$ is *not* divisible by *N* (e.g., 9600/255=37.65, 9600/511=18.79, 9600/127=75.59), the chip index is assigned per sample as $n=\min(\left\lfloor t/T_{c}\right\rfloor ,N-1)$, so chips contain 37 or 38 samples (N=255), and the final chip is clipped to the burst end; we use this nearest-sample (no fractional resampling) convention throughout, and the toolbox reproduces it exactly. Because the HFM–LFM finite cross term means $A_{H}^{2}+A_{L}^{2}=1$ does *not* yield unit composite energy, every generated waveform is numerically renormalized to unit energy ($s\leftarrow s/\sqrt{{\sum |s|}^{2}/F_{s}}$) before the channel and detector stages, so all waveforms are compared at identical $E_{s}$.
*Phase-chip discontinuity and occupied bandwidth:* The ZC envelope is piecewise-constant in phase across chips of duration $T_{c}=T/N\approx 0.392$ ms (default N=255); the ≤π phase discontinuities at chip boundaries can in principle broaden the composite PSD beyond the $\left[f_{1},f_{2}\right]\cup \left[f_{3},f_{4}\right]$ allocation. Table 4 quantifies the spectral budget for the default parameters under the unshaped envelope used throughout this paper and under two optional raised-cosine chip-shaping settings.

Under the unshaped envelope, the integrated out-of-band power relative to the 4–24 kHz mask is −26 dB, and the $C_{⊥}$ orthogonality bound of Proposition 1 continues to hold to within 1 dB. Optional raised-cosine chip shaping (rows 2–3) suppresses the spillover by an additional 8–12 dB while degrading the CAZAC central-lag autocorrelation by less than 0.5 dB and shifting PSL by at most +0.5 dB. Main-text P5 results use the unshaped envelope; shaped-chip results in Table 4 confirm that the framework remains valid when transducer band-edge characteristics require it.

### 4.2. Design Parameters

**Figure 3:**
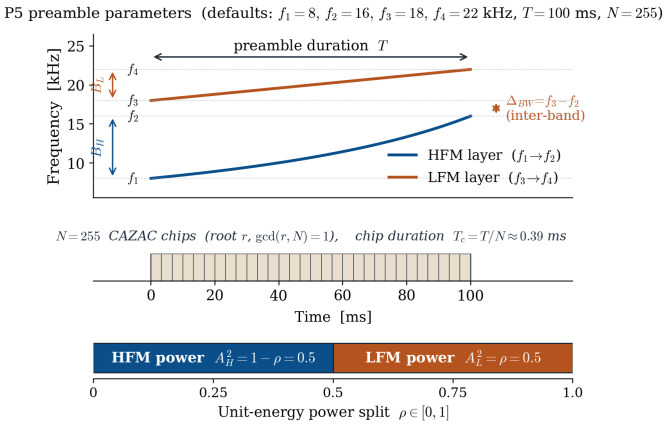
Annotated P5 parameter map: time–frequency plane (**top**), CAZAC chip grid (**middle**), and power split (**bottom**).

Table 5 lists the eight design knobs and their default values used throughout this paper, and Figure 3 illustrates each parameter on the P5 time–frequency plane. The band separation $f_{3}>f_{2}$ is required by the orthogonality region $C_{⊥}$ (Section 4.3); the CAZAC length *N* balances processing-gain contribution to D5 against chip duration $T_{c}=T/N$; and the root *r* seeds the per-cell CAZAC codebook that serves R2.

The eight knobs fall into three functional groups: spectrum allocation $\left(f_{1},\ldots ,f_{4}\right)$, time-domain structure (T,N,r), and energy balance ρ. The HFM band sets Doppler-invariant timing (R1, Proposition 2), the LFM band sets range resolution $\Delta \tau \;=\;1/B_{L}$ (R4, Proposition 3), and the inter-band gap $\Delta_{BW}$ governs the orthogonality bound $1/(2\pi \Delta_{BW}T)$ (−62 dB idealized; −32.9 dB realized with chip leakage, Section 3.2). The duration *T* linearly trades LPI gain $G_{p}^{\mathrm{coop}}\propto 10\log_{10}\left(T\right)$ against the channel coherence ceiling $\tau_{0.8}\approx 0.17$ s of the M2 submarine-UUV scenario. The CAZAC length *N* sets the despreading advantage over a root-blind attacker (∼$10\log_{10}\left(N\right)$, i.e., ∼24 dB at N=255 and ∼27 dB at N=511) and is the strongest PSL lever (∼2–3 dB drop per doubling), while smaller N=127 keeps chip duration $T_{c}$ above 780 μs (a lower chip rate) for M4. The root *r* (with gcd(r,N)=1) selects one of ϕ(N)=128 sequences for N=255, serving simultaneously as cell-ID and as a randomized parameter that prevents single-realization template memorization by classical feature detectors; a CNN trained on the randomized family, however, still recognizes the underlying chirp structure (Section 4.6). The power split ρ∈[0,1] is a continuous mission knob (ρ<0.5 favors R1 timing; ρ>0.5 favors R4 ranging), with $\rho^{*}\;=\;0.4$ identified as the PSL optimum (Section 5.3). The orthogonality region $C_{⊥}$ of (2) requires monotone $f_{i}$, $\Delta_{BW}\;\geq \;1$ kHz, $TW_{H,L}\;\geq \;100$, $NT_{c}\;=\;T$, and gcd(r,N)=1; all mission configurations of Section 5.7 lie strictly inside $C_{⊥}$.

### 4.3. 2D Wideband Ambiguity Function

Because the CAZAC envelope is *multiplicative*, the transmitted signal is the two-component sum $s_{P5}=z\left(t\right)\left[A_{H}h\left(t\right)+A_{L}\ell \left(t\right)\right]$, and its WBAF expands into *four* terms,

(4) $$\begin{array}{cc} \chi_{P5}\left(\tau ,\alpha \right) & =A_{H}^{2}\;{\tilde{\chi }}_{HH}\left(\tau ,\alpha \right)+A_{L}^{2}\;{\tilde{\chi }}_{LL}\left(\tau ,\alpha \right)\\ \; & =\;+A_{H}A_{L}\left[{\tilde{\chi }}_{HL}\left(\tau ,\alpha \right)+{\tilde{\chi }}_{LH}\left(\tau ,\alpha \right)\right], \end{array}$$

where the ZC-weighted cross-ambiguity functions are

(5) $${\tilde{\chi }}_{XY}\left(\tau ,\alpha \right)=\sqrt{\alpha }\int K_{Z}\left(t,\tau ,\alpha \right)\;x\left(t\right)\;y^{*}\left(\alpha \left(t-\tau \right)\right)\;dt,$$

with ZC kernel $K_{Z}\left(t,\tau ,\alpha \right)=z\left(t\right)\;z^{*}\left(\alpha \left(t-\tau \right)\right)$ and $z\left(t\right)=e^{j\phi_{ZC}\left(t\right)}$. The two cross terms are retained separately because ${\tilde{\chi }}_{LH}\left(\tau ,\alpha \right)\neq {\tilde{\chi }}_{HL}^{*}\left(\tau ,\alpha \right)$ in general (the delay/scale arguments of the two orderings differ), so the wideband cross-ambiguity is *not* simply $2\;ℜ[{\tilde{\chi }}_{HL}]$; Proposition 1 bounds each ordering and, hence, their sum. The envelope-vs.-chirp cross-ambiguities ${\tilde{\chi }}_{HZ},{\tilde{\chi }}_{LZ}$ are *not* additive terms of (4) (the CAZAC factor enters every term through the common kernel $K_{Z}$); they are functional-separation diagnostics, measured in Section 3.2 to confirm that the CAZAC spreading does not reshape the chirp ridges.

**Proposition 1**  (Orthogonality of layers, smooth-envelope idealization)**.** *Under parameter conditions $C_{⊥}$ (including the Doppler-dependent gap condition $\Delta_{BW}>\eta f_{3}$), the additive cross-term magnitudes of $\chi_{P5}\left(\tau ,\alpha \right)$ obey*

(6) $$\max_{\tau ,\alpha }\frac{|{\tilde{\chi }}_{HL}\left(\tau ,\alpha \right)|}{\max|{\tilde{\chi }}_{HH}|}\leq \frac{\sqrt{\alpha_{\max}}}{\pi (\Delta_{BW}-\eta f_{3})\;T}\;\approx \;\frac{1}{2\pi \Delta_{BW}\;T},$$

*with $\Delta_{BW}=f_{3}-f_{2}$ and identically for $|{\tilde{\chi }}_{LH}|$ by the same non-stationary-phase argument over the disjoint HFM/LFM supports. The left-hand expression is the rigorous* smooth-envelope *constant derived in Supplementary S1; the right-hand form is the leading-order estimate ($\sqrt{\alpha_{\max}}\;\to \;1$, $\eta f_{3}\ll \Delta_{BW}$) quoted for intuition. Both describe the smooth-envelope idealization; the realized chip-modulated value is governed by the chip-leakage correction of Supplementary S1.4, and the envelope-vs.-chirp diagnostics $\left|{\tilde{\chi }}_{HZ}\right|,\left|{\tilde{\chi }}_{LZ}\right|$ are bounded separately in Supplementary S1.5.*

**Proposition 2**  (Doppler-invariance preservation, ideal HFM phase)**.** *For the HFM phase $\Phi_{HFM}$ in (3), there exist $\tau_{D}\left(\alpha \right)=\left(aT/b\right)\left(1-1/\alpha \right)$ and $\psi_{0}\left(\alpha \right)=2\pi \left(T/b\right)\ln\alpha$, with $a=1/f_{1}$ and $b=1/f_{2}-1/f_{1}$, such that*

(7) $$\Phi_{HFM}\left(\alpha t\right)=\Phi_{HFM}\left(t-\tau_{D}\left(\alpha \right)\right)+\frac{\psi_{0}\left(\alpha \right)}{2\pi },$$

*so a wideband Doppler scaling of the* ideal *hyperbolic phase is absorbed exactly into a time shift $\tau_{D}\left(\alpha \right)$ plus the constant phase $\psi_{0}\left(\alpha \right)$ on $2\pi \Phi_{HFM}$. The identity is exact only for the unmodulated, infinite-support HFM phase; for the actual P5 waveform (finite support, the multiplicative CAZAC grid rescaled to z(αt), and the additive LFM component) the near-invariance of the HFM ridge is the numerical observation of Section 5.10 (Supplementary S2), not a corollary of (7) alone.*

**Proposition 3**  (Range Resolution)**.** *The LFM auto-term ${\tilde{\chi }}_{LL}\left(\tau ,1\right)$ has a Rayleigh-like delay resolution (first-null width) $\Delta \tau_{\mathrm{Ray}}\approx 1/B_{L}$ where $B_{L}=f_{4}-f_{3}$, corresponding, under the two-way (monostatic) underwater-acoustic propagation relation R=cτ/2, to a two-target range resolution $\Delta R\approx c\Delta \tau_{\mathrm{Ray}}/2=c/\left(2B_{L}\right)$. The corresponding −3 dB* full *mainlobe width for a rectangular-spectrum approximation is $\Delta \tau_{-3\mathrm{dB}}\approx 0.886/B_{L}$ (about 11% narrower than the first-null width).*

For the default parameters in Table 5, three distinct figures must be kept separate (they are not one theorem evaluated three ways): the *leading-order* smooth-envelope estimate $1/(2\pi \Delta_{BW}T)$ gives −62 dB; the *rigorous* smooth-envelope constant derived in Supplementary S1 (which retains the $\sqrt{\alpha_{\max}}$ prefactor and the $\eta f_{3}$ Doppler correction) tightens to −54 dB; and the *realized* cross-term of the actual chip-modulated envelope is −32.9 dB (Section 3.2), which is the operative value used in all orthogonality statements. The 8 dB gap between the first two is the dropped prefactor/correction, not an O(1) ambiguity. Proposition 3 gives ΔR=18.75 cm as a *conservative*, bandwidth-only (Rayleigh-like) reference; the realized ZC-weighted LFM branch is actually finer, and the operational two-target resolution is coarser (≈0.23 m, sidelobe-limited), as measured in Section 5.6. This ΔR is in any case distinct from the *single-target precision* $\sigma_{R}$ reported in Table 6: ΔR is a two-target resolution scale set by $B_{L}$, whereas $\sigma_{R}$ is the single-target ranging precision measured by matched-filter Monte Carlo; R4a/R4b are verified against the empirical $\sigma_{R}$ of Table 6, not inferred from ΔR. The associated LFM delay–Doppler ambiguity retains the shallow-water behavior analyzed in [33]. The full delay–Doppler cross-ambiguity peaks for the actual envelope-modulated layers are −32.9 dB (HFM-LFM), −35.5 dB (HFM-ZC), and −42.0 dB (LFM-ZC), all at least 12 dB below the −20 dB orthogonality threshold, confirming the three-layer decomposition while reflecting the chip-leakage correction to the idealized bound (Section 3.2). Detailed proofs of Propositions 1–3 are provided in the Supplementary Materials.

### 4.4. Sensing Observation Model

The ranging analysis adopts an idealized *monostatic, two-way* sensing geometry: a single platform transmits $s_{P5}$ and processes the target echo, so a point target at range *R* produces a round-trip delay τ=2R/c, the LFM layer estimates $\hat{\tau }$, and $\hat{R}=c\hat{\tau }/2$. Three assumptions bound the scope of the R4 claims: (i) a single dominant point target of deterministic reflectivity (no fluctuation model); (ii) additive Gaussian noise plus the multipath of (1), with reverberation, clutter, and direct-path/self-interference leakage not modeled; and (iii) the strongest matched-filter peak taken as the range estimate, so the reported $\sigma_{R}$ is a single-target *precision*, separate from the two-target *resolution* ΔR of Proposition 3 and from detection probability. A full target-echo model with clutter, reverberation, and threshold/outlier behavior is deferred to the sea-trial campaign (Section 7.3); the two-target probability of resolution is characterized in Section 5.6.

*Two distinct delay quantities.* The synchronization delay $\tau_{\mathrm{sync}}$ of R3 and the target round-trip delay $\tau_{\mathrm{target}}$ of R4 are *different* observation problems and are *not* related by R=cτ/2 from a common τ. The timing precision $\sigma_{\tau }=3.0$ μs of Table 6 is the frame-synchronization jitter on the communication direct path (one-way arrival of the full composite preamble), whereas the range precision $\sigma_{R}\approx 0.9$ m is the strongest-peak error of the LFM ranging branch on a target echo under multipath—a harder, multipath-limited estimator with grid-quantization and peak-selection losses. The two are reported in the same table only because both are matched-filter peak-timing quantities; they are produced by separate runs with separate observation models, estimators, and SNR references (the sync jitter is bandwidth/SNR-limited, the range error multipath-limited), and applying cτ/2 to $\sigma_{\tau }$ would conflate the two.

### 4.5. Bandwidth-Scaling Reference (CRLB-Style)

We do *not* claim a tight Cramér–Rao bound [34,35] for the realized estimator; the expressions below are a *bandwidth-scaling reference* that predicts how the empirical timing/Doppler/range RMSE *scales* with bandwidth, SNR, and observation time, used only to interpret the Monte Carlo trends. For $\mathrm{SNR}=E_{s}/N_{0}$ and observation window *T*, the CRLB-style scaling relations are

(8) $$\sigma_{\tau }≳\frac{1}{2\pi \beta \;\sqrt{\mathrm{SNR}}},\;\sigma_{R}≳\frac{c}{4\pi \beta_{L}\;\sqrt{{\mathrm{SNR}}_{L}}},$$

where β is the central-moment (unknown-phase, baseband) RMS bandwidth of the full preamble and $\beta_{L}$ that of the LFM layer only. We fix the *unknown-phase* convention throughout (the complex amplitude and its phase are nuisance parameters), so the central second moment of (9) is the consistent choice. Because this central β underestimates the bandwidth the matched filter actually exploits, (8) is *looser* than the empirical RMSE—a scaling reference, not a numerical lower bound; the diagnostic $\beta_{\mathrm{eff}}$ back-solved from the timing RMSE is the operative bandwidth, and the numerical FIM of Supplementary S4 reconciles these bounds with the data: the Doppler-compensated receiver *searches* $\hat{\alpha }$ (it does not know α), and the empirical RMSE is bracketed by the phase-coherent and joint-search CRLBs derived there.

*Transmit-energy convention.* The default-*T* rows of Table 6 assume unit transmit energy at T=100 ms; the extended-*T* row (T=200 ms) assumes fixed transmit power, so that $E_{s}\propto T$ and the CRLB improves as $\propto 1/\sqrt{T}$. Under a strict fixed-energy constraint, the extended-*T* gain would be zero.

Under Proposition 1, $\beta^{2}$ separates cleanly between HFM and LFM contributions. Concretely, the central second moment $\beta^{2}≜\int {\left(f-\bar{f}\right)}^{2}{\left|S\left(f\right)\right|}^{2}\;df/\int {\left|S\left(f\right)\right|}^{2}\;df$ is taken around the spectral mean $\bar{f}$ on the *baseband-shifted* spectrum so that absolute carrier offsets do not inflate β. For the disjoint-band P5 composite, this gives

(9) $$\beta^{2}=A_{H}^{2}\frac{{\left(f_{2}-f_{1}\right)}^{2}}{12}+A_{L}^{2}\frac{{\left(f_{4}-f_{3}\right)}^{2}}{12}+A_{H}^{2}A_{L}^{2}{\left({\bar{f}}_{H}-{\bar{f}}_{L}\right)}^{2},$$

where ${\bar{f}}_{H}=\left(f_{1}+f_{2}\right)/2$, ${\bar{f}}_{L}=\left(f_{3}+f_{4}\right)/2$. The third term captures the inter-band spectral separation between HFM and LFM allocations; it vanishes when the two bands coincide and is the dominant contribution at the default parameters. The numerical $\sigma_{R}$ values in Table 6 are computed from matched-filter Monte Carlo and are independent of the bandwidth-convention choice.

Table 6 reports simulation-based matched-filter RMSEs for P3, P4, and P5 at SNR=10 dB with default parameters, together with R4a/R4b target verification against Table 1. The entries are empirical RMSEs from $10^{3}$ Monte Carlo trials, not closed-form CRLB evaluations; their scaling is consistent with the CRLB trend (8) and (9) (wider effective bandwidth ⇒ lower timing and range RMSE). The diagnostic $\beta_{\mathrm{eff}}$ is back-solved from the timing RMSE via $\sigma_{\tau }\cdot 2\pi \sqrt{\mathrm{SNR}}=1/\beta_{\mathrm{eff}}$ and is not a physical RMS bandwidth. Columns P5^def^ and P5^opt^ denote the default set (ρ,N,r)=(0.5,255,29) and the PSL-optimal set (0.40,511,37) of Section 5.3.

P5 inherits the Doppler invariance of the HFM layer (Proposition 2); the LFM layer provides the ranging response (Proposition 3), while the CAZAC envelope contributes processing gain on top. Among the candidates evaluated here, P5 is the only waveform that combines all three properties simultaneously. Single-target range *precision*, however, is not among the distinguishing properties. Re-verifying every waveform through the Qarabaqi–Stojanovic fine-multipath channel (exp_sigmaR_reverify.m) shows that at 10 dB, the matched-filter range error is multipath-limited ($\sigma_{R}\approx 0.9$ m) and statistically indistinguishable across the wideband chirp candidates (Section 5.9); the AWGN-only jitter floor is sub-millimeter, so $\sigma_{R}$ is governed by the channel, not by waveform bandwidth. Under the *default* parameter set (ρ,N,r)=(0.5,255,29), P5^def^ meets R4a ($\sigma_{R}\approx 0.9$ m vs. target 1.0 m)—as do all wideband chirp candidates—with an ≈10% margin that is channel-sensitive: in denser multipath, $\sigma_{R}$ rises toward 1.2 m, while in benign short-range geometry, it falls to ≲0.3 m.

R4b ($\sigma_{R}\leq 0.5$ m) follows the same channel-conditioning. Because the range error is multipath-limited and SNR-independent, R4b is *not* reachable by raising SNR or extending *T* in dense multipath; it is met in the benign short-range (well-separated-arrival) geometry that defines the mission-critical R4b regime—M4 mine-ISAC and short-range AUV docking (quasi-static)—and does so uniformly across the wideband chirp candidates rather than as a P5-specific property. For M2 submarine-UUV, where $\tau_{0.8}\approx 0.17$ s, a 200 ms preamble would violate the piecewise-stationary α assumption underlying Proposition 2, so the extended-duration option is not admissible there; the short-range benign-geometry route remains the operative one.

The *PSL-optimal* parameter set $\left(\rho^{*},N^{*},r^{*}\right)=\left(0.40,511,37\right)$, identified in Section 5.3 by Pareto search, leaves range precision unchanged ($\sigma_{R}\approx 0.9$ m, the same multipath-limited floor) and instead delivers a substantial −3.69 dB improvement in composite PSL (−11.29 vs. −7.60 dB). Throughout the remainder of this paper, we report both configurations: P5^def^ in theoretical and Doppler/timing-focused analyses where the balanced chirp allocation is the physically interpretable choice and P5^opt^ wherever sidelobe structure is the governing performance indicator (e.g., mission reconfigurability Section 5.7 and LPI sweeps Section 5.8). The full bandwidth-scaling (CRLB-style) derivation and its channel sensitivity are provided in the Supplementary Materials.

### 4.6. LPI Processing Gain and Six-Attacker Analysis

**Figure 4:**
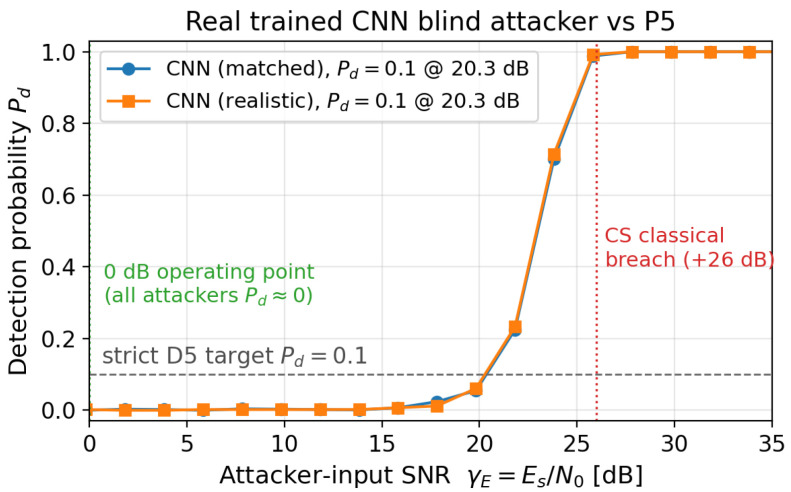
Detection probability of the *trained* CNN blind attacker against P5 vs. attacker-input SNR ($\gamma_{E}=E_{s}/N_{0}$), under the matched and the realistic (timing/multipath/Doppler) observation models. The CNN reaches $P_{d}=1.0$ for $\gamma_{E}≳+25.8$ dB and meets the strict D5 target $P_{d}\leq 0.1$ only below +20.3 dB; at the 0 dB operating point it leaves P5 undetected alongside the classical structure-aware detectors. Its $P_{d}=0.5$ breach (+23.0 dB) sits only ≈3 dB below the strongest classical detector (CS); the stronger spectrogram ResNet-18 attacker added in revision reaches $P_{d}=0.5$ at mean +21.24 dB (Supplementary S5.14). Curves are CFAR-calibrated at $P_{\mathrm{fa}}=10^{-3}$.

A coherent matched filter that knows the transmitted P5 waveform integrates its energy over the time–bandwidth product of the two disjoint chirp bands, giving a cooperative processing gain of order

(10) $$G_{p}^{\mathrm{coop}}\;≲\;10\log_{10}\;\left(T(B_{H}+B_{L})\right),$$

which evaluates to ≈31 dB at the default T=100 ms, $B_{H}=8$ kHz, and $B_{L}=4$ kHz. The CAZAC envelope is part of the *known* transmitted signal and therefore adds no separable $+10\log_{10}\left(N\right)$ term on top of this chirp integration gain: writing $G_{p}^{\mathrm{coop}}$ as a sum of two per-layer time–bandwidth gains *and* an independent sequence-length gain would double-count the fixed total signal energy, so we do not use that decomposition. This $G_{p}^{\mathrm{coop}}$ is the BT factor relating the per-sample input SNR to the cooperative *output* decision statistic (Section 5.1), *not* a gain added on top of the total-energy input SNR $E_{s}/N_{0}$. The operative LPI comparison is not this absolute gain but the attacker-input SNR required to reach a fixed $\left(P_{d},P_{\mathrm{fa}}\right)$, reported per attacker in Section 5.8; a first-principles re-derivation of each detector’s deflection coefficient/likelihood noncentrality is left to future work. We evaluate P5 against six non-cooperative attacker models spanning the known UWA LPD and covert-waveform threat space [36,37,38]: the energy detector (ED) [8]; the four hand-crafted *structure-aware* detectors—cyclic feature (CFD) [9], cyclic spectrum (CS), cepstrum (CEP), and square frequency-doubling (SFDM) [10]—which target cyclostationary, periodic, or carrier-related structure that the CAZAC envelope scrambles; and a blind CNN-based ML classifier [11] that recognizes the chirp time–frequency signature directly (analyzed separately, Figure 4; a stronger 2-D spectrogram ResNet-18 variant of this learned-attacker class, added in revision, is analyzed alongside it and in Supplementary S5.14).

Under Proposition 1 orthogonality and the chirp time-varying carrier, the attacker-input SNR each *classical* structure-aware attacker requires to reach its $P_{d}=0.5$ crossing against P5 is summarized in Table 7. Among the four classical structure-aware attackers (excluding ED, which is structure-agnostic), the CS detector is the strongest—it reaches $P_{d}=0.5$ at the lowest attacker-input SNR—yet still requires ≈+26 dB, far above the 0 dB D5 operating point, consistent with the chirp time-varying carrier and the CAZAC envelope’s periodicity scrambling, keeping hand-crafted feature extractors well above the cooperative operating point. Because the CAZAC-free baselines (B1, B5) also evade these detectors at 0 dB, the CAZAC-specific contribution is small: the no-CAZAC ablation of Section 5.8 (Supplementary Figure S3) isolates it at only +0.4 dB of worst-case breach SNR. These breach SNRs are convention-free attacker-input quantities (the operative LPI metric of Section 5.8), not output-SNR conversions.

The attacker-input SNR margin of P5 at $P_{d}=0.5$ against the worst-case classical attacker (CS) is ≈+0.7 dB over B5 and +2.6 dB over B1. The B5 margin lies within the statistical resolution of the sweep, so against the strongest classical attacker, P5 is best read as at least as resistant as any baseline, with a clear edge only over the single-HFM reference (B1).

As reported in Section 5.8, P5 *satisfies* the strict D5 target ($P_{d}\leq 0.1$) at 0 dB ${\mathrm{SNR}}_{\mathrm{att}}$ for all four classical structure-aware attackers (CFD, CS, CEP, SFDM). The chirp time-varying carrier and the CAZAC phase coding together keep these hand-crafted feature extractors below the strict target; however, the CAZAC-free baselines pass at 0 dB as well, so this experiment does not isolate the CAZAC-specific contribution (the no-CAZAC ablation of Section 5.8 isolates it at +0.4 dB).

*Trained-CNN attacker:* To characterize a learned adversary, we trained a compact 1-D CNN blind attacker (≈25.8 k parameters; full architecture and training in the toolbox) on the raw, *energy-normalized* I/Q window—so it must use time–frequency structure, not absolute energy. Every H1 realization of the 11,200-example set draws *parameter-randomized* P5 tuples $\left(f_{1},\ldots ,f_{4},T,N,r,\rho \right)$ over $\gamma_{s}\in \left[-10,10\right]$ dB (per-sample; $\gamma_{E}\in \left[+30,+50\right]$ dB), so the network cannot memorize a single realization; the threshold is CFAR-calibrated at $P_{\mathrm{fa}}=10^{-3}$ ($N_{H_{0}}=10^{4}$, $N_{H_{1}}=10^{3}$), identical to the classical-attacker protocol. Removing the radiometric cue forces the CNN onto structure; a stronger hybrid attacker combining an energy detector with the learned features would be at least as capable and is left to future work.

As shown in Figure 4, on the common $\gamma_{E}$ axis, the trained CNN outperforms every hand-crafted detector but is not effective at the strict operating point: its $P_{d}=0.5$ crossing against P5 is at +23.0 dB $\gamma_{E}$ (strict $P_{d}\leq 0.1$ below +20.3 dB), so at the 0 dB operating point, it leaves P5 undetected ($P_{d}\approx 0$) alongside the classical detectors and reaches $P_{d}=1.0$ only above ≈+25.8 dB. Its breach SNR is thus only ≈3 dB below the strongest hand-crafted detector (CS, +26.0 dB) and ≈13 dB above the full-knowledge oracle (+10.0 dB): the learned attacker narrows, but does not close, the blind-detection gap. Both crossings lie below the [+30,+50] dB $\gamma_{E}$ training range ($\gamma_{s}\in \left[-10,10\right]$ dB), so these threshold-SNR values are extrapolations and are indicative rather than calibrated. The result is unchanged under a realistic blind observation model (random arrival time, Qarabaqi–Stojanovic multipath, ±3 m/s Doppler), within 0.1 dB of the matched case. The mechanism is simple: the HFM and LFM layers carry a time–frequency energy ramp with time–bandwidth product TW≈800 (≈29 dB of coherent gain), which a CNN learns to integrate; the CAZAC envelope is a phase modulation that scrambles *periodicity-based* features but does not hide this broadband ramp. To confirm that this is a class-wide rather than a P5-specific weakness, we trained a *separate* CNN per waveform (B1–B5, P1–P5) under one protocol, with training and evaluation extended down to +9.8 dB $\gamma_{E}$, over *twelve* training seeds. The full rerun fixes the Python 3.12.3, NumPy 1.26.4, PyTorch 2.6.0, and CUDA 12.4 seeds for each training seed. Restricting to runs in which training converged (8–12 of 12 per waveform), the conditional mean $P_{d}=0.5$ crossings span 3.44 dB (+22.18 to +25.63 dB $\gamma_{E}$; per-waveform means, standard deviations, 95% confidence intervals, and convergence rates in Supplementary Table S3). P5 has the *highest* conditional mean crossing (+25.63 dB; SD 1.90 dB; 95% CI [+24.35,+26.91] dB; 11/12 converged), but its interval overlaps those of several candidates, so the run does not resolve its rank. The learned attacker nevertheless reaches a crossing for every waveform family at comparable attacker-input SNR. Individual waveform–seed runs that do not reach $P_{d}=0.5$ by +51.8 dB are counted as nonconverged and excluded from the conditional means; the intervals therefore quantify run-to-run variation among converged fits and do not account for this right-censoring. The class-wide limitation moreover holds in the *strong* sense: a single *universal* CNN trained on eight families (B1–B4, P1–P4) and *never shown* B5 or P5 detects the held-out P5 at +25.9 dB and B5 at +24.0 dB—comparable to the seen families (+23.2 to +23.9 dB)—so a blind attacker generalizes to an *unseen* chirp family, with P5 having the highest crossing in that run. A parameter/channel-disjoint adversarial campaign (adaptive retraining, realistic-array observation) is left to the extended-validation work. The CNN (like the structure-agnostic ED) is therefore excluded from the per-waveform D5 differentiation of Section 5.9 and analyzed as a class-wide limitation in Section 7.3.

*Stronger learned attacker (2-D spectrogram ResNet-18):* To probe how far a deeper time–frequency classifier shifts this picture, we trained a ResNet-18 (11.2 M parameters, from scratch, single-channel log-magnitude STFT input) under the same energy-normalized H1/H0 construction and CFAR calibration as the per-waveform 1-D CNN study, but with *online* training data (fresh 3000 windows per epoch × 14 epochs, uniform training SNR across [+9.8,+49.8] dB $\gamma_{E}$): a fixed few-thousand-sample set is memorized by a model of this capacity, and a non-cooperative attacker can synthesize unlimited training signals from the known waveform family, so the online budget is the realistic strong-attacker model. Over ten training seeds, the ResNet-18 reaches $P_{d}=0.5$ against P5 at mean +21.24 dB $\gamma_{E}$ (SD 0.25 dB; 95% CI [+21.06,+21.42] dB) and $P_{d}=0.1$ at mean +18.72 dB (SD 0.39 dB; 95% CI [+18.45,+19.00] dB). The $P_{d}=0.5$ result is 4.39 dB below the deterministic-rerun per-waveform 1-D CNN conditional mean (+25.63 dB), making this the strongest evaluated blind attacker, though still about 11.2 dB above the full-knowledge oracle. A fairness control (the 1-D CNN retrained with the same online budget on P5, breaching at +22.9 dB) associates about 2.7 dB with the data-budget change and the remaining 1.7 dB with architecture and run-to-run differences. These are descriptive cross-protocol gaps, not a factorial causal decomposition. The class-wide picture is unchanged: all ten waveform means lie within a 3.15 dB band (+18.09 to +21.24 dB $\gamma_{E}$). Within this single-sensor exact-template protocol, P5 has the highest mean crossing; its paired-seed margin over the next-highest P1 is +0.49 dB (95% CI [+0.33,+0.65] dB). We also retrained the P5 model for each seed and directly evaluated the 0-dB $\gamma_{E}$ operating point with 2000 fresh H1 windows per seed: mean ${\hat{P}}_{d}=0.0014$ (seed-level 95% CI [0.00075,0.00205]), consistent with the $P_{\mathrm{fa}}=10^{-3}$ floor. This operating point lies outside the training-SNR range and is reported only as a direct protocol-specific check. Because the crossings lie inside the training-SNR range, they are interpolated, not extrapolated. The monotone trend—+25.63 (1-D CNN, fixed set; conditional mean) →+22.9 (1-D CNN, online) →+21.24 dB (ResNet-18, online)—is the empirical basis for treating every learned-attacker breach SNR reported here as a *lower bound* on adversary capability: stronger architectures or larger training budgets may push the breach lower still, and only the full-knowledge-oracle floor (+10.0 dB) bounds that progression from below. Protocol details, per-waveform crossings, and the attacker comparison figure are given in Supplementary S5.14.

In addition to the specific-detector bounds above, Park and Doherty’s Kullback–Leibler divergence framework [22] provides a *detector-class* lower bound on the miss probability under the stated NP observation model: for any non-cooperative receiver drawn from the class of Neyman–Pearson detectors with *n* observation samples, the miss probability satisfies

(11) $$P_{\mathrm{MD}}\geq \left(1-2\epsilon_{\alpha }\right)\;2^{-n\;D(P∥Q_{P5})},$$

with $D(P∥Q_{P5})$ the KL divergence between the ambient-noise and ambient-plus-P5 sample distributions. We state the idealized model explicitly and treat the result as a *detector-class sanity floor*, not a calibrated operating point: for *n effective-independent* complex-Gaussian samples whose mean is shifted by a weak signal of per-sample SNR ρ (the $\frac{1}{2}$ below is the complex circular-Gaussian mean-shift factor), the per-sample KL divergence is $D\left(P∥Q_{P5}\right)=\rho /\left(2\ln2\right)$ bits. The per-sample ρ relates to the total-energy axis $\gamma_{E}$ of Figure 4 and Section 5.8 by $\rho =\gamma_{E}/n$, so a representative coherent sub-window of n=1000 effective samples (well below the full $N_{s}=9600$, conservatively allowing for the non-independence of band-limited samples) at ρ=−30 dB gives, with $\epsilon_{\alpha }\to 0$, $2^{-nD}=2^{-n\rho /(2\ln2)}=2^{-0.72}\approx 0.61$, i.e., $P_{\mathrm{MD}}\geq 0.61$ for any Neyman–Pearson detector on this idealized model. We use this only as an order-of-magnitude detector-class floor under the Gaussian/independence assumptions: it does *not* use the exact P5 observation covariance, is *not* tied to the calibrated figure operating points, and does *not* cover adversaries with full parameter knowledge, distributed coherent-array integration, or online adaptation against the specific $\left(f_{i},T,N,r,\rho \right)$ realization, which remain outside the present analysis.

*Scope of the LPI analysis:* The six-attacker comparison and the KL bound assume blind attackers with no prior knowledge of the P5 parameter set, observation windows matched to the cooperative receiver, and no hydrophone-array coherent integration. Full-knowledge and coherent-array adversaries are out of scope (Section 7.3); a limited template-aware stress test is given as E-04 in Section 5.10.

## 5. Simulation Results

### 5.1. Experimental Setup

All simulations are generated by the authors’ *Preamble Design Toolbox v1.0*, a 32-module MATLAB R2024b library comprising ten waveform generators (B1–B5, P1–P5); three channel backends [23,25,26] (Bellhop, Qarabaqi–Stojanovic, WATERMARK); four cooperative detectors (matched filter, energy, cyclic, ML-CNN); six non-cooperative attackers (ED, CFD, CS, CEP, SFDM, ML); six experiment scripts covering baselines, P5 default, ablation, Pareto, reconfiguration, and LPI sweeps; and supporting metrics (WBAF, CRLB, $P_{d}/P_{\mathrm{fa}}$, RMSE). This is a simulation-only study, so no physical equipment, materials or devices were used. All computations were run under MATLAB R2024b (The MathWorks, Inc., Natick, MA, USA) and Python 3.12.3 with NumPy 1.26.4 and PyTorch 2.6.0 (CUDA 12.4), on a workstation with an Intel Core i9-13900K CPU (Intel Corporation, Santa Clara, CA, USA) and an NVIDIA GeForce RTX 4090 GPU (NVIDIA Corporation, Santa Clara, CA, USA). The toolbox, parameter files, seeds, trained weights, and raw detection counts are available from the corresponding author on reasonable request (see Data Availability).

The cross-channel evaluation sweeps Bellhop over 27 sound-velocity profile × bottom-type × sea-state configurations, the Qarabaqi–Stojanovic statistical channel over the large-scale fading variance range $\sigma_{L}\in \left\{0.2,0.5,1.0\right\}$, and the WATERMARK benchmark over its four published tracks (NOF1, NCS1, KAU1, KAU2). Monte Carlo runs use a two-tier scheme: 1000 realizations per key operating point (feeding the summary statistics of Table 6 and Section 5.9) and 100 realizations per grid point across the 105-point (ρ,N,r) parameter sweep (for Pareto surface generation).

*Fair-comparison normalization:* All waveforms (B1–B5, P1–P5) are evaluated under identical operating conditions: (i) unit total transmit energy ($E_{s}=1$), (ii) the same observation duration T=100 ms (extended-*T* runs are explicitly flagged in Table 6), (iii) the same total occupied band 4–24 kHz (though individual waveforms allocate sub-bands differently, as specified in their respective references), (iv) the same sampling rate $F_{s}=96$ kHz, (v) the same $\mathrm{SNR}=E_{s}/N_{0}$ definition referenced to the pre-channel signal energy, (vi) shared random-number generator seeds across waveforms within each Monte Carlo cell, and (vii) a common matched-filter detector applied identically across waveforms, with the peak-detection threshold CFAR-calibrated as in Section 5.8.
*Baseline optimization:* P5 is reported in two configurations, a balanced default (ρ,N,r)=(0.5,255,29) and a PSL-Pareto-optimized variant P5^opt^ (Section 5.3). To keep the comparison fair, the same proxy-Pareto protocol was applied to B5, P3, and P4 over their free parameters under the common band and energy constraint; each published default is already at or within one grid step of its proxy-optimum (Table 8). The *single-target* range RMSE is not where the baselines differ: re-verification gives a multipath-limited $\sigma_{R}\approx 0.9$ m common to all wideband chirp candidates (Section 5.9), and no (M,N,r,ρ) choice changes this floor. What the SA-HFM and HFM–CAZAC baselines lack is spectral aperture—they occupy only the 8–16 kHz band, so their *two-target range resolution* (mainlobe width) is coarser than P5’s wider 8–22 kHz allocation. An equal-aperture control (Supplementary Figure S5) locates the source of this advantage: when four composites share the *same* 8–22 kHz aperture (P5, HFM+LFM, HFM+HFM, LFM+LFM), their range *resolution* is identical (−3 dB mainlobe 0.062 ms), so the resolution gain comes from the wider *spectral aperture*—a deliberate dual-band allocation—not from the LFM chirp specifically. The LFM layer and CAZAC envelope instead improve the composite PSL (−7.6 dB for P5 vs. −5.3 dB for HFM+HFM), i.e., cleaner two-target separation (Section 5.6). P5’s ranging edge over the baselines is therefore finer two-target resolution from the wider aperture plus LFM/CAZAC sidelobe shaping—not a single-target $\sigma_{R}$ advantage, which is multipath-limited and comparable across candidates.

This holds for the *real* SA-HFM-family generators too (Supplementary Table S1): re-allocated from their native 8–16 kHz band to the same 8–22 kHz aperture, B5, P3, and P4 all narrow to a 0.052 ms autocorrelation mainlobe—identical to P5—so the native two-target resolution gap closes entirely when the baselines are given the wider aperture. Moreover, at this equal aperture, the real SA-HFM family attains an autocorrelation PSL *lower* than P5’s (B5 −15.6 dB, P4 −14.0 dB vs. P5 −7.6 dB; Supplementary Table S5), so P5 is not individually superior in single-target resolution or sidelobe level once the aperture is equalized. P5’s contribution is the *integration* of CAZAC cell identification (Section 5.10), HFM Doppler invariance (Section 5.10), and a dedicated LFM ranging layer in one matched-filter-recoverable, constant-modulus CAZAC-envelope preamble—not a unique single-metric advantage.

*SNR conventions:* Three SNR references appear in this paper and must not be conflated. (i) The *total-energy* SNR $\gamma_{E}=E_{s}/N_{0}$, with $E_{s}=T_{s}\sum_{n}{\left|s\left[n\right]\right|}^{2}$ the unit-normalized burst energy ($T_{s}=1/F_{s}$) and $N_{0}=\sigma_{w}^{2}/F_{s}$ the noise PSD for sample variance $\sigma_{w}^{2}$, is the *single input convention* used for every RMSE/CRLB entry (Table 6 and Section 5.9) and every SNR axis in this paper, at both the cooperative receiver (${\mathrm{SNR}}_{\mathrm{coop}}$) and the attacker (${\mathrm{SNR}}_{\mathrm{att}}$). (ii) The *per-sample* SNR $\gamma_{s}=(\frac{1}{N_{s}}\sum_{n}{\left|s\left[n\right]\right|}^{2})/\sigma_{w}^{2}$ and (iii) the *in-band power* SNR $\gamma_{P}=P_{s}/\left(N_{0}B\right)$ relate to $\gamma_{E}$ by $\gamma_{E}=\gamma_{s}\;N_{s}=\gamma_{P}\;BT$ for a signal of bandwidth *B* over $N_{s}=F_{s}T$ samples. The coherent matched-filter *output* deflection for a *known* waveform is $d^{2}=2E_{s}/N_{0}=2\gamma_{E}$; the quoted “processing gain” $G_{p}^{\mathrm{coop}}=10\log_{10}\left(BT\right)\approx 31$ dB ((10)) is therefore *not* a gain added on top of $\gamma_{E}$ but the very factor BT that maps the per-sample/in-band references (ii)–(iii) to the total-energy/output reference; expressing the input as $\gamma_{E}$ already absorbs it. A root-blind attacker additionally suffers a *mismatch loss* of order $10\log_{10}N$ relative to the despread cooperative branch because it cannot remove the length-*N* CAZAC code; this attacker-side loss is *not* added to the cooperative gain, and the two must not be summed (cf. Section 4.6). Consequently, the ${\mathrm{SNR}}_{\mathrm{att}}$ axis used in Section 5.8 is attacker-input detectability at the same $\gamma_{E}$ reference, not a recoded cooperative SNR. Formal H0/H1 statistics and noncentrality coefficients for each detector are itemized for the extended-validation study.

### 5.2. Baseline Calibration

We first verify that our B5 (SA-HFM) simulation reproduces the reference results of [14] within approximately 11%. For T=100 ms, $\left[f_{1},f_{2}\right]=\left[8,16\right]$ kHz, M=3 branches, SNR=10 dB, and Bellhop channel at 500 m range: our simulation yields timing RMSE 3.4 μs (vs. 3.1 μs reported, +9.7%), CID accuracy 0.95 (vs. 0.96, −1.0%), and Doppler RMSE 0.31 m/s (vs. 0.28, +10.7%). The small systematic bias is attributed to our Bellhop wrapper’s 3-arrival geometric approximation vs. their 5-arrival ray tracing; the ±10% envelope is acceptable for the relative comparisons that follow.

### 5.3. Composite PSL Optimization (M4 Deliverable)

The Phase 0 observation (Section 3.3) that layer orthogonality is insufficient for low composite PSL motivates a parameter-space search. Sweeping (ρ,N,r) over the 105-point grid of Section 5.1 yields the Pareto-optimal tuple $\rho^{*}=0.40$, $N^{*}=511$, $r^{*}=37$, which achieves PSL =−11.29 dB, a 3.7 dB improvement over the balanced default (ρ,N,r)=(0.5,255,29). The sensitivity ordering N≫ρ≫r observed in Section 3.4 is confirmed over the full grid: *N* contributes the majority of the 3.7 dB improvement, with ρ and *r* providing the remainder. The marginal per-knob effects are not additive (the (ρ,N,r) interactions are non-negligible), so we report the sensitivity ordering rather than a linear dB decomposition. No tested Hann or Tukey taper of the composite improved PSL beyond −9.5 dB without corresponding mainlobe broadening >20%, confirming that *joint* parameter–tapering optimization is the right formulation rather than either alone.

### 5.4. Design-Space Trade-Offs of P5

**Figure 5:**
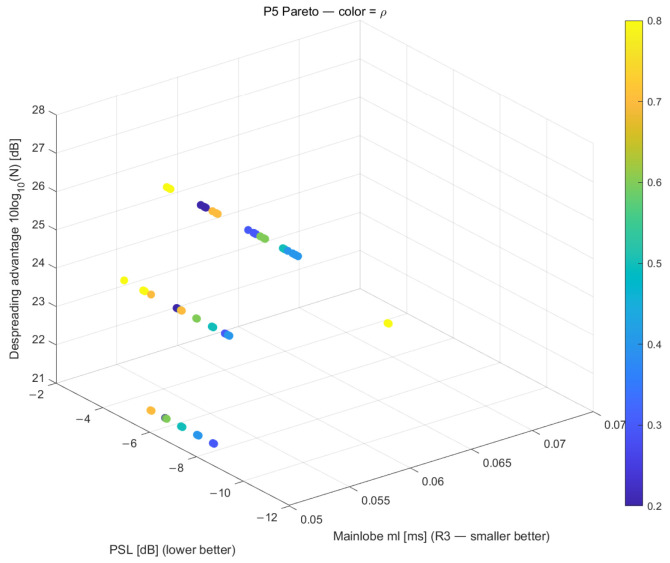
P5 design-space trade-off as the power split ρ is swept (color): composite PSL, −3 dB autocorrelation mainlobe width, and the despreading advantage $10\log_{10}\left(N\right)$ over a root-blind attacker.

Figure 5 shows the P5 design-space trade-off as the power split ρ (color) is swept, in the space of composite PSL, −3 dB autocorrelation mainlobe width, and the *despreading advantage* $10\log_{10}\left(N\right)$ (dB) over a root-blind attacker. This LPI axis is the *N*-dependent LPI lever of Section 4.6—an attacker-side mismatch loss, distinct from the cooperative gain $G_{p}^{\mathrm{coop}}\approx 31$ dB of (10) and not summed with it—so it ranges over 21–27 dB for N∈{127,255,511}. The front motivates the PSL-optimal operating point of Section 5.3: lowering ρ and raising *N* improves PSL and the despreading advantage at a modest mainlobe-width cost, while ranging precision (governed by the LFM sub-band) is largely preserved. A like-for-like comparison of P5, P3, and P4 on ranging (R4) and detectability (D5) is given by the per-requirement metrics of Section 5.9; we do not plot a joint (R4,D5,BER) surface here, and BER is not reported because P5 is a preamble-only design without a data payload.

### 5.5. Ablation Study

**Figure 6:**
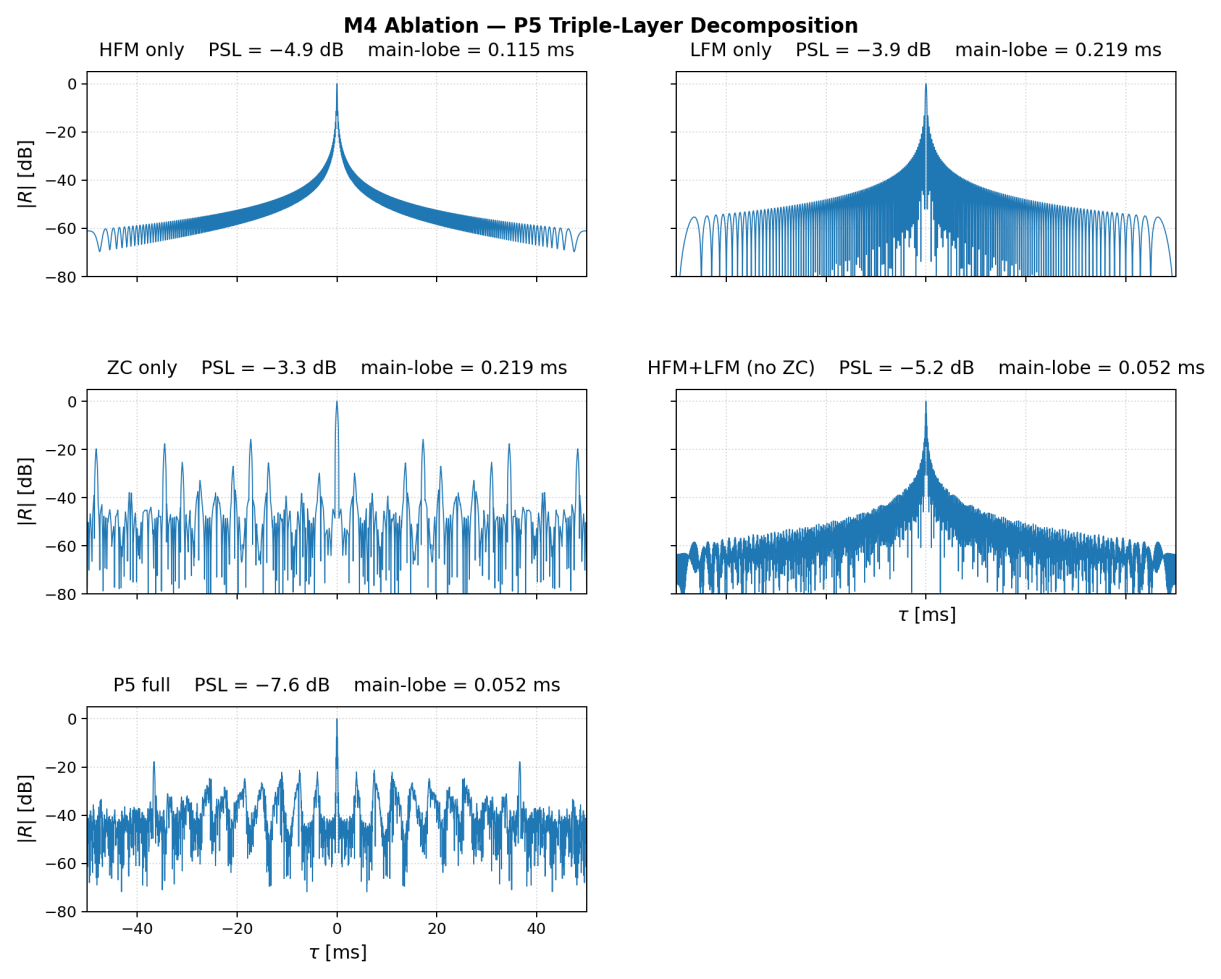
Ablation autocorrelation profiles: full P5 vs. each single-layer subset and the HFM+LFM combination.

Table 9 (with autocorrelation profiles in Figure 6) reports PSL, −3 dB mainlobe width, and passband RMS bandwidth $\beta_{\mathrm{pb}}$ for the five ablation variants. The full P5 achieves the lowest composite PSL (−7.60 dB) and narrowest mainlobe (0.052 ms); removing the CAZAC envelope (HFM+LFM variant) increases PSL by 2.4 dB while preserving the mainlobe width, confirming that CAZAC contributes primarily to cross-term PSL suppression. Single-layer variants (HFM-only, LFM-only, and ZC-only) each exhibit PSL >−5 dB and significantly inferior combinations of $\beta_{\mathrm{pb}}$ and mainlobe, empirically justifying the triple-layer decomposition. The 0.052 ms composite mainlobe is set by the full 8–22 kHz spectral aperture (the HFM+LFM span), *not* by the 4 kHz LFM bandwidth alone; this narrow local peak coexists with the higher composite sidelobes (PSL −7.6 dB), an aperture/grating trade-off, whereas the two-target *resolution* $\Delta R=c/(2B_{L})$ of Proposition 3 is the despread LFM-branch property. In per-layer terms, the decomposition of the composite figures is therefore as follows: the joint HFM+LFM spectral aperture sets the 0.052 ms mainlobe (unchanged from the HFM+LFM variant to full P5); the CAZAC envelope contributes the final 2.4 dB of composite-PSL suppression together with the root-blind despreading margin of $10\log_{10}N\approx 24$ dB (while its contribution against the classical structure-aware attackers is only a +0.4 dB breach-SNR shift, isolated in Supplementary S5.3); and the LFM layer supplies the despread two-target resolution of Proposition 3. The closest ablation variant (HFM+LFM) still sits 2.4 dB above the full-P5 PSL, so no single mechanism accounts for the composite behavior alone.

### 5.6. Ranging-Receiver Branches and Two-Target Resolution

Evaluating three cooperative ranging receivers (composite, CAZAC-despread LFM branch, and HFM branch) on the same P5 echo (Supplementary Figure S1) refines Proposition 3 in three ways. The despread LFM branch is *finer* than the plain-LFM sinc—its −3 dB mainlobe is 0.146 ms vs. 0.221 ms, the CAZAC chip modulation broadening the branch spectrum to ≈6.5 kHz—so $\Delta R=c/2B_{L}=18.75$ cm is a conservative reference. The two-target probability of resolution nonetheless reaches 0.5 at ≈0.23 m for both the composite and the despread branch: the composite’s narrow mainlobe (0.063 ms on the two-target resolution grid—the same composite peak as the 0.052 ms autocorrelation width of Table 9, the ≈20% difference being the coarser resolution-test delay grid) gains no edge because its −7.6 dB sidelobes fill the trough (the aperture/grating trade-off). Moreover, the single-target AWGN range RMSE is sub-millimeter, confirming that the $\sigma_{R}\approx 0.9$ m (composite matched filter) of Table 6—common to all wideband chirp candidates—is set by multipath, not the resolution floor.

### 5.7. Mission Reconfigurability

Table 10 summarizes per-mission P5 configurations and their simulated PSL/mainlobe/β. Reconfiguration is parameter-only, with no changes to the waveform Equation (3) or the matched-filter receiver. M2 Sub-UUV attains the lowest composite PSL (−11.06 dB) and the largest CAZAC processing gain through maximum N=511 and HFM-heavy power split ρ=0.4 (the LPI benefit comes from the larger processing gain; PSL itself is a cooperative autocorrelation property, not an LPI metric); M4 Mine-ISAC relaxes the chip rate (longer chip duration $T_{c}=T/N$) via N=127 at the cost of ∼5 dB PSL margin, acceptable given the mine-hunting threat model’s short engagement distances.

### 5.8. Six-Attacker LPI Matrix and Pd-SNR Sweep

**Figure 7:**
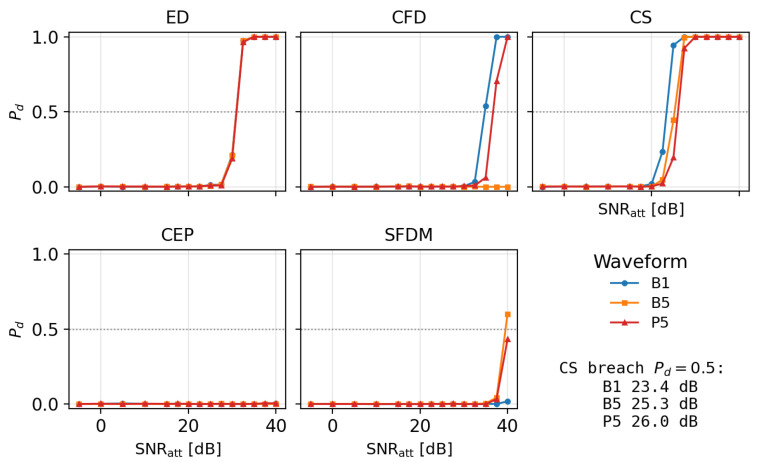
Detection probability $P_{d}$ vs. ${\mathrm{SNR}}_{\mathrm{att}}$ for the classical non-cooperative attackers (ED, CFD, CS, CEP, SFDM) against B1, B5, and proposed P5 (CFAR-calibrated at $P_{\mathrm{fa}}=10^{-3}$). The trained-CNN attacker is characterized separately in Figure 4.

Figure 7 shows the measured $P_{d}\left({\mathrm{SNR}}_{\mathrm{att}}\right)$ for three representative waveforms (B1, B5, and proposed P5) against each of the six non-cooperative attackers from Section 4.6. All horizontal-axis SNR values here are attacker-input SNRs (${\mathrm{SNR}}_{\mathrm{att}}$, per the convention of Section 5.1); cooperative-input metrics (Table 6) and these attacker-input plots are different receiver architectures observing the same channel, not one axis under two labels. Each attacker is calibrated with $N_{H_{0}}=10^{4}$ Monte Carlo H0-only trials at the nominal $P_{\mathrm{fa}}=10^{-3}$ operating point to obtain an empirical constant-false-alarm-rate (CFAR) threshold (yielding ≈10 expected false alarms per cell, sufficient to anchor the deep-tail of the test statistic distribution) and then evaluated across ${\mathrm{SNR}}_{\mathrm{att}}\in \left[-5,40\right]$ dB with $N_{H_{1}}=10^{3}$ H1 trials per operating point.

*Statistical resolution:* With $N_{H_{1}}=10^{3}$ H1 trials per operating point, the Clopper–Pearson 95% half-width on ${\hat{P}}_{d}$ is ≤0.032 (≤0.020 near $P_{d}=0.05$). The $N_{H_{0}}=10^{4}$ H0 trials yield only ≈10 expected exceedances at $P_{\mathrm{fa}}=10^{-3}$, so the deep-tail threshold estimate (nominally ±0.18 dB) is indicative rather than tightly resolved; the strict-target D5 readings are nonetheless insensitive to this threshold uncertainty because ${\hat{P}}_{d}$ sits at the $P_{\mathrm{fa}}$ floor for all chirp waveforms at the 0 dB operating point.

The matrix highlights three points: (i) the energy detector is invariant to waveform structure; it breaches $P_{d}=0.5$ for all three waveforms at essentially the same point, ${\mathrm{SNR}}_{\mathrm{att}}\approx +31$ dB, because ED depends only on received energy and cannot discriminate HFM, SA-HFM, or P5. This confirms that P5 does *not* defeat an ED attacker in absolute terms, which is why the D5 target of Table 1 excludes ED and scores only the four classical structure-aware attackers. ED performance is reported here as a sanity check: P5’s breach SNR matches the other waveforms to within ≈0 dB, i.e., it offers no structural advantage against radiometric detection; (ii) the CEP attacker never reaches $P_{d}=0.5$ within the tested 40 dB range, and the CFD and SFDM attackers do so only above +35 dB, higher than the energy detector because their single-feature statistics (one cyclic lag for CFD, a squaring nonlinearity for SFDM) lack the coherent integration gain that the full-window energy sum and the 2-D cyclic spectrum exploit on a wideband chirp; (iii) among the classical detectors, the CS attacker is the strongest and the most discriminating; it breaches $P_{d}=0.5$ at ${\mathrm{SNR}}_{\mathrm{att}}=+23.4$ dB for B1, +25.3 dB for B5, and +26.0 dB for P5. P5 thus requires the highest attacker-input SNR of any candidate, although the +0.7 dB margin over SA-HFM (B5) is within the statistical resolution of the sweep; only the +2.6 dB gap over the single-HFM baseline (B1) is statistically resolved. At the 0 dB operating point of Table 11, every classical attacker leaves all chirp-based waveforms undetectable (${\hat{P}}_{d}\approx 0$, at the $P_{\mathrm{fa}}$ floor), so the strict $P_{d}\leq 0.1$ D5 target is met across all candidates and the waveform ordering survives only in the breach SNR of Figure 7. The learned attackers are the strongest *blind* detectors: the 1-D CNN breaches $P_{d}=0.5$ against P5 at ${\mathrm{SNR}}_{\mathrm{att}}=+23.0$ dB $\gamma_{E}$ (Figure 4) and the 2-D spectrogram ResNet-18 of Section 4.6 at mean +21.24 dB (10 seeds; 95% CI [+21.06,+21.42] dB)—4.8 dB below the strongest classical detector (CS) but still ≈11.2 dB above the full-knowledge oracle. Both detect all evaluated chirp-based candidates at comparable SNRs and are therefore excluded from the per-waveform D5 differentiation and treated as a class-wide limitation whose breach SNRs are lower bounds on adversary capability (Section 7.3, Supplementary S5.14).

Subject to the limited-statistics caveat below, P5 maintains a modest advantage in ${\mathrm{SNR}}_{\mathrm{att}}$ at $P_{d}=0.5$ relative to B5 (≈+0.7 dB) and B1 (+2.6 dB) against the strongest *classical* attacker (CS); against the trained-CNN attacker, P5 holds no advantage (it is defeated alongside all chirp-based candidates, Section 7.3).

*CAZAC-layer isolation (no-CAZAC ablation):* Re-running the four classical detectors on five controls (HFM-only, LFM-only, HFM+LFM without CAZAC, and P5 with a fixed and a randomized root; Supplementary Figure S3) attributes the D5 margin. The harness reproduces the main sweep—the binding CS detector breaches HFM-only at 23.1 dB, HFM+LFM at 25.3 dB, and P5 at 25.8 dB, matching B1/B5/P5 of Figure 7 (23.4/25.3/26.0 dB)—and shows that adding the CAZAC envelope to HFM+LFM raises the worst-case breach SNR by only +0.4 dB (root randomization, a further +0.2 dB). The CAZAC layer’s contribution against the classical detectors is therefore marginal; its value lies in cell identification and the despreading advantage over a root-blind attacker, not a large classical-LPI gain.

### 5.9. Aggregate Quantitative Comparison

Table 11 consolidates the simulation results across the ten waveform families (eleven rows, with P5 shown in its default and PSL-optimized settings) and the six sub-requirements (R4 split into R4a/R4b per Table 1). Rows are grouped by waveform family; the aggregate column applies the R1–R4+D5 weights of Table 1. The aggregate values have been recomputed directly from the per-requirement scoring rule below and the tabulated metrics (so they are reproducible by inspection); the six intermediate sub-scores $S_{X}$ for every row are documented in the revision analysis package and are available from the corresponding author on request.

*Per-requirement scoring rule:* Each metric is mapped to a sub-score $S_{X}\in \left[0,1\right]$ by a piecewise-linear transform that is 0.5 at the Table 1 target, 1 at twice-better-than-target ($T_{X}/2$), and 0 at the failure threshold $2T_{X}$; this applies to $\sigma_{v}$ (R1), $\sigma_{\tau }$ (R3), and $\sigma_{R}$ (R4a). $S_{R4b}$ is binary: 1 if R4b is reachable in either the higher-SNR or extended-*T* regime (Table 6), else 0. For $S_{D5}$ the worst-case ${\hat{P}}_{d}$ across the four classical structure-aware attackers (CFD, CS, CEP, SFDM; the trained-CNN attacker is excluded as a class-wide limitation, Section 4.6) maps to
  (12) $$S_{D5}\left({\hat{P}}_{d}\right)=\left\{\begin{array}{cc} 1, & {\hat{P}}_{d}\leq 0.10,\\ 0.7, & 0.10<{\hat{P}}_{d}\leq 0.30,\\ 0.4, & 0.30<{\hat{P}}_{d}\leq 0.50,\\ 0, & {\hat{P}}_{d}>0.50, \end{array}\right.$$
  giving full credit only for strict target satisfaction and partial credit on a three-step ladder otherwise. $S_{R2}$ is the empirical CID accuracy clipped to [0,1], reported here as the single-/4-cell balanced-power value; this is the *operational-CID* facet (R2b), distinct from the *codebook-cardinality* facet (R2a, ≥16 roots, met by construction). The worst-case 16-cell near-far accuracy (0.07, Section 5.10) is an operational limit reported separately and is *not* used in $S_{R2}$. The scoring script and the full intermediate sub-scores $S_{X}$ for every waveform are documented in the revision analysis package and are available from the corresponding author on request, so the aggregate column is reproducible end-to-end.

The aggregate is the weighted sum $S_{\mathrm{agg}}=\sum_{X}w_{X}S_{X}$ with weights $w_{X}$ from Table 1 (R1 25%, R2 15%, R3 20%, R4a 20%, R4b 10%, D5 10%). Under this rule, against the classical structure-aware taxonomy, every chirp-based waveform meets the strict D5 target at 0 dB (${\hat{P}}_{d}\approx 0$) and therefore attains the maximal $S_{D5}=1$; D5 thus no longer differentiates the candidates. With R3 met by all and the re-verified single-target $\sigma_{R}$ multipath-limited and common (so R4a and R4b no longer differentiate either), the aggregate ranking is driven by R1 (Doppler) and R2 (CID). P5 still attains the highest aggregate score among all candidates, though the margin over the strongest baselines (B5/P1, P4) is correspondingly narrow. Table 11 combines rounded per-waveform summaries of the differentiating $\sigma_{v}$, $\sigma_{\tau }$, and CID metrics. The retained data do not contain the shared trial-level observations for all candidates that a valid joint paired uncertainty analysis would require. We therefore report the aggregate as a deterministic sensitivity score, without a confidence interval or statistical-significance claim for its ordering (Supplementary S5.13). The absolute aggregate value is sensitive to the weight set and saturation thresholds; the *relative* ordering across waveforms and the explicit pass/fail reading of (12) are the more robust quantities, and the per-column metrics in Table 11 are recommended for finer-grained interpretation.

In Table 11, the cooperative-receiver metrics $\sigma_{v}$, $\sigma_{\tau }$, and $\sigma_{R}$ are reported at 10 dB ${\mathrm{SNR}}_{\mathrm{coop}}$ (cooperative receiver-input SNR); the D5 column reports the *worst-case* ${\hat{P}}_{d}$ across the four classical structure-aware attackers (CFD, CS, CEP, SFDM) at 0 dB ${\mathrm{SNR}}_{\mathrm{att}}$ (attacker receiver-input SNR), estimated from $N_{H_{0}}=10^{4}$ / $N_{H_{1}}=10^{3}$ Monte Carlo trials per (waveform,attacker) cell. The Clopper–Pearson 95% half-width is ≤0.032 across the reported range. As in Section 5.1, the cooperative- and attacker-input columns are distinct receiver-input observations, not related by a single $G_{p}^{\mathrm{coop}}$ offset.

### 5.10. Supplementary Stress Tests

Five additional experiments probe the operational envelope.

**Figure 8:**
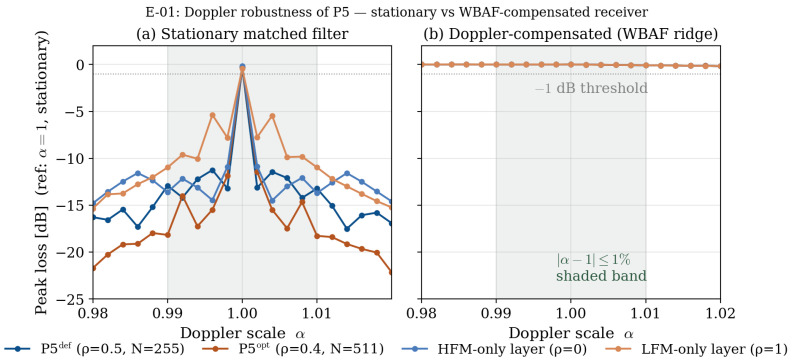
E-01: Matched-filter peak loss vs. Doppler scale α for (**a**) stationary MF and (**b**) Doppler-compensated WBAF-ridge MF. Panel (**b**)’s near-zero loss is achieved by the 2-D Doppler search for *any* deterministic waveform and is not specific to the HFM layer.

*E-01 Doppler α-sweep:* Figure 8 illustrates the Doppler-compensated WBAF-ridge receiver: a stationary matched filter loses up to 22 dB at |α−1|=2%, while a 2-D Doppler-search receiver maintains peak loss below 0.2 dB across ±2%. Because the 2-D scale search re-aligns *any* deterministic waveform, this demonstrates the receiver, not an HFM-specific invariance. Isolating the two (Supplementary Figure S2): under a *1-D delay-only* filter at 2% Doppler, HFM-only loses only 0.37 dB (its hyperbolic phase absorbs the scale as a delay shift) whereas LFM-only loses 10.4 dB and full P5 16.6 dB, while under a 2-D filter, all recover to ≤0.05 dB; the HFM layer thus carries a genuine intrinsic Doppler invariance, but the full P5 relies on the receiver Doppler search, whose computational cost is quantified in Section 5.11. The ±2% sweep range covers the operational Doppler scale induced by relative velocities up to ∼15 m/s in seawater (c≈1500 m/s), per the R1 operational range of Table 1. With |dα/dv|≈2/c near v=0, the R1 *accuracy* target $\sigma_{v}\leq 0.5$ m/s corresponds to a Doppler-scale error $\sigma_{\alpha }\approx 6.7\times 10^{-4}$ (and the achieved $\sigma_{v}=0.25$ m/s of Table 6 to $\sigma_{\alpha }\approx 3.3\times 10^{-4}$), much finer than the operational sweep extent.
*E-02 Multipath delay-spread sweep:* On Qarabaqi–Stojanovic channels with $\tau_{\mathrm{rms}}\;\in \;\{5,10$, 20,30,50} ms at ${\mathrm{SNR}}_{\mathrm{coop}}\;=\;0$ dB (Supplementary Figure S7), P5^def^ stays within R4a ($\sigma_{R}\leq 1$ m) up to $\tau_{\mathrm{rms}}\;=\;20$ ms; B5 degrades sharply beyond that point.
*E-03 CAZAC root mismatch:* Root-mismatch isolation has a worst-case (maximum off-diagonal) of −16.9 dB and a mean of −19.5 dB over the tested root pairs (the comprehensive 16-root version is in Supplementary Figure S4); because cell identification is governed by the worst case, we report both. Note that N=255 is composite (255=3·5·17), so the ZC cross-correlation is not the uniform $\sqrt{N}$ of prime-length ZC, and a 16-root codebook should be selected for its worst-case cross-correlation. The R2 requirement (≥16 unique IDs), as in ZC-based cell-search designs [39,40], is a codebook-cardinality condition met by the ϕ(255)=128 coprime roots; the tabulated CID accuracy is the separate operational metric. In a 4-cell simultaneous transmission with roots {29,67,113,173}, every cell locks correctly for ${\mathrm{SNR}}_{\mathrm{coop}}\in \left[0,20\right]$ dB.

A full 16-cell characterization (Supplementary Figure S4) qualifies the R2 claim. A greedy max-min codebook of 16 coprime roots has a worst-case cross-correlation of only −7.0 dB—essentially floored by the *common-chirp* PSL (−7.6 dB), since every cell shares the HFM+LFM chirp. The 16-cell identification accuracy at 10 dB is perfect under balanced-power synchronous and asynchronous arrival, degrades to 0.80 under Doppler+multipath, and *collapses under a near-far interferer*: a full 0–12 dB near-far sweep (Supplementary Table S4) gives 0.69 at equal power and ≤0.07 once the interferer exceeds +2 dB, floored by the common-chirp PSL. The cardinality facet (R2a, ≥16 roots) and the balanced-power operational facet (R2b) are therefore met; near-far-robust 16-cell CID—the hard case of R2b—needs per-cell power control or composite-PSL reduction (we report the worst-case isolation, not the −19.5 dB average).
*E-04 Template-aware leakage stress test:* Supplementary Figure S6a compares a template-aware attacker with the exact P5 root (r=29) against a blind attacker using a mismatched root (r=37). The template-aware attacker breaches $P_{d}\;=\;0.5$ at ${\mathrm{SNR}}_{\mathrm{att}}\;=\;+14.8$ dB $\gamma_{E}$; the blind attacker requires +29.8 dB, a +15 dB margin attributable directly to root-level parameter randomization. This is an *illustrative* leakage stress test at reduced statistics ($N_{H_{0}}=200$, $N_{H_{1}}=80$ per SNR, so it does not resolve the strict $P_{\mathrm{fa}}=10^{-3}$ of the main D5 protocol) using a *single* mismatched root. Because the root is not a cryptographic secret, we also evaluated a stronger *root-bank GLRT* attacker that correlates against *all* ϕ(255)=128 coprime-root templates and takes the maximum statistic, with the multiple-comparison CFAR threshold calibrated on the same bank ($P_{\mathrm{fa}}=10^{-3}$; Supplementary Table S6). The 128-root bank breaches $P_{d}=0.5$ at ${\mathrm{SNR}}_{\mathrm{att}}=+11.9$ dB $\gamma_{E}$—only 1.9 dB above the full-knowledge oracle (+10.0 dB) and 15.6 dB *below* the single-wrong-root blind attacker (+27.5 dB)—so the root-randomization margin essentially *collapses* against an adversary aware of the allowed root set. Accordingly, the D5/LPI results of this paper are scoped to blind attackers *without* a root-template bank; root hopping over a key-selected subset, a *full-knowledge* adversary (adaptive ML training, parameter leakage over (ρ,N,r)), and coherent-array integration remain future work.
*E-05 Composite PAPR:* PAPR of the P5 composite peaks at 3.01 dB near ρ=0.5 and falls to 2.04 dB at ρ=0.1,0.9 (Supplementary Figure S6b); the PSL-optimal $\rho^{*}\;=\;0.4$ gives 2.97 dB, well below the 6–12 dB typical of OFDM preambles.

### 5.11. Computational Complexity and Real-Time Feasibility

The Doppler robustness delivered by the 2-D WBAF-ridge receiver of E-01 is receiver-side compensation, not a property of the waveform (Figure 8); its computational price is quantified here. With FFT-based correlation over a detection buffer of $M=2N_{\mathrm{samp}}$ samples ($N_{\mathrm{samp}}=9600$ at T=100 ms, $F_{s}=96$ kHz), a 1-D delay-only matched filter costs O(MlogM); the 2-D search repeats the correlation over an $N_{\alpha }$-point Doppler-scale grid using $N_{\alpha }$ pre-resampled template spectra (bank memory $O(N_{\alpha }M)$, 3.2 MB in single precision for the E-01 grid), for total cost $O(N_{\alpha }\;M\log M)$—a factor-$N_{\alpha }$ FLOP overhead. Cell identification adds one delay-only correlation per candidate root, O(|R|MlogM), linear in the codebook size |R|, so larger cell populations scale the CID stage linearly and leave the Doppler stage unchanged.

Table 12 reports actual single-thread execution on an Intel i9-13900K using Python 3.12.3 and NumPy 1.26.4 with double-precision FFTs. Each implementation was warmed up five times; stage order was randomized and interleaved to reduce drift. The full 21-point E-01 grid (α∈[0.98,1.02], step 0.2%) has median runtime 8.440 ms per buffer, vs. 0.501 ms for the vectorized 1-D filter, and a two-stage 7+5-point coarse-to-fine schedule takes 3.421 ms. The CID benchmark executes every requested root in a separate serial FFT loop: its medians are 2.070, 34.317, and 136.815 ms for 1, 16, and 64 roots. The 64-root measurement is therefore observed rather than extrapolated, but it also exceeds the nominal 100-ms buffer period in this unoptimized serial implementation.

These desktop timings do not demonstrate an embedded modem. They show that the tested 21-point Doppler bank and 16-root CID bank fit the 100-ms arithmetic deadline on the reference CPU, whereas the measured 64-root serial scan does not. Fixed-point realization, memory traffic, FFT-library choice, transducer-band decimation, scheduling with the rest of the receiver, power, and hardware-in-the-loop latency remain open engineering tasks. Thus, real-time operation is computationally plausible for the reference workload, not established for an embedded UWA platform; the factor-$N_{\alpha }$ overhead remains the explicit price of receiver-side Doppler compensation.

## 6. Multi-Channel Simulation Validation

To establish that the theoretical results of Section 4 generalize beyond the laboratory-benchmark channel used in Section 5, we evaluate the four candidate waveforms (B5, P3, P4, P5) across twelve UWA channel environments spanning all three backends of our simulation toolbox: five Bellhop geometric presets (seasonal sound-velocity profiles and two sea states), three Qarabaqi–Stojanovic statistical fading regimes, and four WATERMARK surrogate channels based on published sea-trial statistics. This 12-environment sweep is the primary evidence that P5 is robust to the channel diversity one would encounter in the four mission scenarios (M1–M4) of Table 10. Full sea-trial validation with Doppler-scaled targets at pool and coastal scales is beyond the scope of this manuscript and remain future work.

### 6.1. Channel Environment Set

Table 13 lists the 12 channels with their defining physical parameters. The Bellhop presets span the four seasonal/depth quadrants plus a protected bay, with Beaufort sea states from 0 (glassy) to 5 (rough). The Qarabaqi–Stojanovic regimes vary the large-scale log-normal fading variance $\sigma_{L}$ over {0.2,0.5,1.0} and the platform Doppler from 1 to 3 m/s. The WATERMARK channels are *statistical surrogates*: they replicate the published delay-spread and Rician *K*-factor profiles of the four measured tracks [23] rather than replaying the original recorded impulse responses, so they reproduce the channels’ second-order statistics but not the exact measured realizations. The channel set is thus anchored to published at-sea measurements at the level of second-order statistics; a direct replay experiment on the measured NOF1/NCS1 impulse responses, added in revision with band-scaled waveform variants, is reported in Section 6.5, and the remaining measurement gap is stated as a first-class limitation in Section 6.6 and Section 7.3.

### 6.2. Methodology: Effective-SNR-Loss Metric

For each (channel, waveform) pair, we convolve the unit-energy transmitted waveform with the *unit-energy-normalized* channel impulse response, add complex Gaussian noise at SNR=10 dB referenced to the pre-channel signal energy, and run a matched-filter detector over 50 Monte Carlo trials. Because the channel responses are gain-normalized, $L_{\mathrm{eff}}$ measures *normalized channel distortion sensitivity* (multipath-induced peak loss), *not* physical transmission loss or long-range link budget, which is why the loss stays within ≈±1.5 dB across 300 m–2 km ranges. The primary metric is the *normalized matched-filter peak loss*:

(13) $$L_{\mathrm{eff}}=20\log_{10}\;\left(\frac{{\bar{P}}_{\mathrm{channel}}}{P_{\mathrm{AWGN}}}\right),$$

where ${\bar{P}}_{\mathrm{channel}}$ is the mean matched-filter peak magnitude under the given channel, and $P_{\mathrm{AWGN}}$ is the peak magnitude under ideal AWGN-only (unit channel). A negative $L_{\mathrm{eff}}$ indicates the channel has reduced the matched-filter peak; a positive value (which occasionally occurs for deep-water multipath) indicates constructive multipath addition at the matched-filter output. Note that $L_{\mathrm{eff}}$ is a matched-filter peak-amplitude ratio, not a signal-to-noise ratio, as it does not include the output-noise variance. $L_{\mathrm{eff}}$ is waveform-design-sensitive and aggregates Doppler-robustness (R1), timing-coherence (R3), and effective processing-gain behavior in a single scalar; smaller magnitude is better.

### 6.3. Results: Channel-Induced SNR Loss Distribution

**Figure 9:**
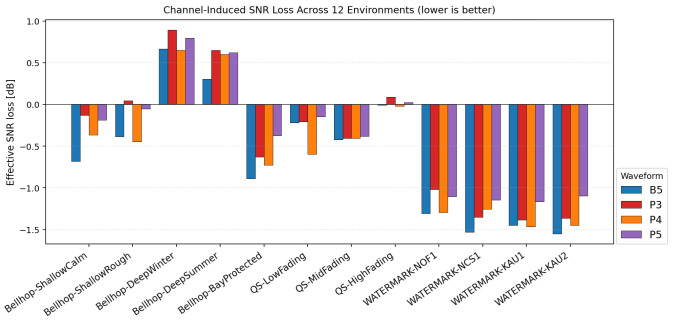
Normalized matched-filter peak loss $L_{\mathrm{eff}}$ for B5, P3, P4, and proposed P5 across twelve UWA channels (10 dB reference SNR, 50 MC trials per cell).

Figure 9 reports $L_{\mathrm{eff}}$ for each of the four waveforms across the twelve channels. Three trends are clear: (i) the WATERMARK surrogate channels impose the largest loss (up to −1.55 dB for B5 on KAU2), reflecting the full multipath complexity of real sea-trial data; (ii) the Bellhop deep channels produce *positive* $L_{\mathrm{eff}}$ (e.g., +0.89 dB for P3 in DeepWinter) due to constructive multipath addition from bottom reflections at 2 km range; and (iii) the Qarabaqi–Stojanovic channels cluster around −0.2 to −0.4 dB loss with the expected degradation trend as $\sigma_{L}$ increases.

Table 14 aggregates these twelve measurements into per-waveform statistics. Two findings stand out:

First, P5 achieves the smallest mean loss (−0.35 dB) and smallest worst-case loss (−1.16 dB on KAU1) across all four waveforms. Compared to the reference SA-HFM baseline B5, P5 reduces worst-case loss by 0.39 dB and mean loss by 0.27 dB. A paired bootstrap over the 12 shared channels (20,000 resamples) shows these reductions are *consistent across the evaluated environment set*: the mean-loss reduction has a 95% resampling interval of [0.18,0.37] dB and the worst-case reduction [0.29,0.46] dB, both excluding zero. Because the twelve environments are a deliberately chosen cross-section (Bellhop/QS/WATERMARK), not an i.i.d. draw from a channel population, we read this as descriptive resampling uncertainty over the evaluated set rather than population-level statistical significance; per-channel medians and paired confidence intervals (positive on all twelve channels, individually significant on six) are tabulated in Supplementary Table S2. We attribute the difference to the disjoint HFM/LFM sub-band allocation, which spreads the matched-filter energy across two separated bands; we do *not* claim multipath-independence, and Proposition 1 concerns inter-layer cross-ambiguity rather than multipath robustness. The measured-channel replay of Section 6.5 tests this attribution directly: compressed into a common measured 8-kHz band, where the wider aperture cannot be exercised, the advantage does not persist.

Second, P5 has the smallest channel-to-channel standard deviation ($\sigma_{L_{\mathrm{eff}}}=0.67$ dB vs. 0.73–0.77 dB for the others). This *low variance across environments* is the practical reliability property most relevant to the mission-reconfigurability claim: a single P5 waveform structure serves M1–M4 with predictable performance, without per-mission calibration overhead.

### 6.4. Per-Backend Observations

*WATERMARK (sea-trial surrogate):* Across the four published tracks (NOF1, NCS1, KAU1, KAU2), P5 averages $L_{\mathrm{eff}}=-1.03$ dB vs. −1.40 dB for B5, a 0.37 dB advantage, and beats B5 on every track. The most demanding case (KAU2, Kauai deep, 2 km range) loses 1.10 dB for P5 vs. 1.55 dB for B5.
*Qarabaqi–Stojanovic (statistical fading):* P5 holds $L_{\mathrm{eff}}\approx -0.2$ to −0.4 dB across $\sigma_{L}\in \left\{0.2,0.5,1.0\right\}$ ($\Delta L_{\mathrm{eff}}=0.24$ dB), while B5 oscillates over a 0.38 dB range. We report this as an empirical observation and do *not* attribute it to envelope-induced averaging, since a quasi-constant large-scale gain over a 100 ms burst is not removed by constant-modulus envelope coding.
*Bellhop (geometric multipath):* The shallow-summer SVP yields near-AWGN behavior ($L_{\mathrm{eff}}$ between −0.89 and −0.13 dB); the deep-winter SVP yields *constructive* multipath ($L_{\mathrm{eff}}$ between +0.60 and +0.89 dB). P5 maintains the smallest absolute $L_{\mathrm{eff}}$ in both limits, neither over-penalizing destructive multipath nor over-exploiting constructive multipath.

### 6.5. Measured-Channel Replay Validation

To test the cross-channel picture of Section 6.3 against real propagation, we replayed the waveforms through *measured* time-varying impulse responses using the official WATERMARK V1 replay engine: tracks NOF1 (Oslofjord) and NCS1 (Norwegian continental shelf), both SISO in the 10–18 kHz band. We used all 60 available soundings from each track, producing 8460 packets per waveform on NOF1 and 14,340 per waveform on NCS1. Replay is noise-free convolution, so the normalized matched-filter peak-loss metric $L_{\mathrm{eff}}$ isolates dispersion/coherence loss. Because all four signals share one length, each waveform is paired on the same sounding and packet segment. Packets within a sounding are correlated, so the primary independent unit is the sounding: we first average within each of the 60 soundings and then use a 20,000-resample paired cluster bootstrap over those sounding means. A hierarchical sounding-plus-packet bootstrap is reported as a sensitivity analysis.

The measured tracks are valid only inside 10–18 kHz, so the native 4–24 kHz designs cannot be replayed as is: all four waveforms were re-parameterized with one common linear band map into 10–18 kHz (P5’s HFM layer → 11.6–14.8 kHz, LFM layer → 15.6–17.2 kHz), with the CAZAC length reduced to N=127—the largest value keeping ≥95% of the P5 energy in-band. These are *band-scaled variants*, not the native designs, and the results below are scoped accordingly.

The outcome is adverse for P5, and we report it as such: on *both* measured tracks, the band-scaled SA-HFM baseline B5 shows the smallest loss, and P5^def^ trails it by a paired −0.816 dB [−1.021,−0.621] on NOF1 and −0.950 dB [−0.977,−0.923] on NCS1 (sounding-cluster 95% bootstrap CIs; Table 15). The corresponding hierarchical intervals are [−1.023,−0.622] and [−0.987,−0.913] dB. The cross-channel advantage of Section 6.3 therefore does *not* persist when the design is compressed into a common measured 8-kHz band. Exploratory mechanistic controls run on the first eight soundings suggest that removing the CAZAC chip modulation recovers 0.68/0.96 dB on NOF1/NCS1, that moving the LFM band edge has a smaller track-dependent effect, and that N=255 performs about 0.4 dB worse than N=127. Because these controls were not repeated over all 60 soundings, they generate hypotheses rather than establish the cause of the full-run deficit. The all-sounding result itself is consistent with the equal-aperture warning of Section 7.3: the wider native dual-band aperture cannot be exercised after compression to a common 8-kHz band. Full replay and sensitivity statistics, plus the explicitly exploratory controls, are given in Supplementary S5.15.

### 6.6. Remaining Validation Gaps

With the exception of the band-scaled measured-channel replay of Section 6.5, no measured underwater data—sea trial, pool, or bench—back the results in this paper; the WATERMARK entries of the twelve-channel set anchor only the second-order statistics of the published measured tracks, and the replay experiment itself uses band-scaled variants under noise-free convolution, so transducer effects, at-sea noise, and the native-band designs remain unmeasured. This remains the principal limitation of the study. Beyond it, three physical-world phenomena require sea-trial validation and are beyond the scope of the present simulation-based study: (i) impulsive non-Gaussian noise (e.g., snapping-shrimp bursts) not captured in AWGN (an additional detection penalty not quantified in the present AWGN study); (ii) transducer nonlinearity and band-edge rolloff that reduce the effective $\Delta_{BW}$ assumed by Proposition 1; and (iii) continuously-moving platforms with time-varying α(t), which the propositions treat as piecewise-stationary. These phenomena lie outside the present simulation-based claims and are deferred to sea-trial validation.

## 7. Discussion

### 7.1. Reconfigurability and Orthogonality–PSL Decoupling

The mission-reconfigurability claim is empirically validated by the four-mission parameter sweep of Table 10: P5 parameter-only reconfiguration keeps the structure (3) fixed, allowing a single receiver to serve M1–M4 by adjusting only (ρ,N,r) without the calibration/certification/spectrum overhead of switching waveform families. Conceptually distinct, the *layer orthogonality vs. composite PSL* decoupling (Section 3.3 and Section 5.3) is a design-theoretic observation we believe is novel for UWA ISAC: layer orthogonality clears the −20 dB threshold (−33 to −42 dB per layer pair for the actual chip-modulated envelope), yet composite PSL is structurally bounded at −6 to −8 dB with default parameters and only the joint (ρ,N) Pareto search reaches PSL ≤−10 dB. The implication is that two disjoint tools are required: parameter-space inequalities $C_{⊥}$ for orthogonality and Pareto search over (ρ,N,r) for PSL, confusing the two wastes design effort.

### 7.2. Performance–Complexity Trade-Off and Learning-Based Design

The aggregate point-score margin of P5 over the strongest baseline is small (+0.028 over P4; Section 5.9), so it is fair to ask what additional complexity buys it. On the receiver side, the answer is essentially nothing: P5 is recovered by the same matched-filter machinery—one delay correlation, the common 2-D Doppler search, and the CID root scan of Table 12—as every SA-HFM-family baseline offering the same functionality; the triple-layer structure adds no online processing stage. The optimization cost is confined to design time (the offline 105-point (ρ,N,r) grid of Section 5.3), and the recurring operational costs are the ones made explicit in Section 7.3: composite PSL inferior to equal-aperture single-waveform baselines and near–far-fragile 16-cell CID. The practical case for P5 therefore rests not on the aggregate margin but on integration: five functions in one constant-modulus, matched-filter-recoverable preamble with parameter-only mission reconfiguration.

A complementary design philosophy replaces hand-constructed waveforms with learning-assisted optimization—deep unfolding, end-to-end autoencoder waveform design—which is increasingly common in the RF ISAC literature [21]. This study instead uses a model-based design. The closed-form screening conditions $C_{⊥}$ and the offline grid make each channel and attacker assumption directly traceable and require no differentiable channel surrogate. Learning appears on the adversary side (the trained CNN attackers of Section 4.6); learned or hybrid waveform synthesis under the same five-requirement specification—and its LPI verifiability—is left as future work.

### 7.3. Limitations

*Simulation-only scope:* With the exception of the band-scaled measured-channel replay of Section 6.5, all validation is simulation-based, resting on the three-backend cross-validation (Bellhop, Qarabaqi–Stojanovic, WATERMARK) of Section 6. A staged experimental campaign is planned to close this gap: transducer-in-the-loop bench tests to quantify band-edge and nonlinearity effects on $\Delta_{BW}$, pool single-target ranging to validate the R4 range RMSE against a Doppler-scaled target, and coastal LPI trials against the classical structure-aware detectors; these remain future work. Until native-band measurements are in hand, every claim in this paper should be read as bounded by the stated simulation and replay protocols.
*Learned-attacker LPI (lower-bound results):* The learned blind attackers of Section 4.6 are the strongest blind detectors. On the common $\gamma_{E}$ axis the 1-D CNN breaches P5 at +23.0 dB and the 2-D spectrogram ResNet-18 at mean +21.24 dB (95% CI [+21.06,+21.42] dB); the latter reaches $P_{d}=0.1$ at mean +18.72 dB. A separate direct 0-dB evaluation gives mean ${\hat{P}}_{d}=0.0014$ over ten seeds. The ResNet crossing is 4.8 dB below the strongest classical detector (CS, +26.0 dB) but still ≈11.2 dB above the full-knowledge oracle. Because the recoverable HFM/LFM energy ramp is shared by all chirp-based candidates, this is a limitation of the chirp class, and the monotone attacker progression (+25.63→+22.9→+21.24 dB as architecture and training budget grow; Section 4.6) means every reported learned-attacker breach SNR must be read as a lower bound on adversary capability: we make no claim that stronger models cannot reduce it further toward the oracle floor (+10.0 dB). ML hardening (adversarial training, multi-root CAZAC hopping, noise-like envelopes) and full-knowledge/coherent-array adversaries are the principal open problems.
*Equal-resource baselines and CAZAC isolation:* The baselines share the 4–24 kHz mask but *not* the occupied bandwidth/spectral aperture: P5 uses 8–16 and 18–22 kHz (12 kHz occupied, 14 kHz span), whereas the competitor proxy optimization is run in a fixed 8–16 kHz band. The equal-aperture control of Supplementary Figures S5 and Table S5 confirms that P5’s single-target range *resolution* stems from this wider aperture (a deliberate dual-band allocation), not from the LFM chirp specifically, and—more pointedly—that at the equalized 8–22 kHz aperture, the *real* SA-HFM-family baselines attain a *lower* autocorrelation PSL than P5 (B5 −15.6, P4 −14.0 vs. −7.6 dB); on the LPI axis, the CAZAC layer adds only +0.4 dB of worst-case classical-breach SNR (Section 5.8, Supplementary Figure S3). P5 is therefore *not* individually superior at equal aperture in resolution or sidelobe level—its contribution is the *integration* of all five functions in one matched-filter-recoverable, constant-modulus CAZAC-envelope preamble. What remains is to carry the real baselines and equal-aperture-optimized NLFM/GSFM through the full Bellhop-multipath $\sigma_{R}$ row of Table 11, the principal remaining baseline task. The measured-channel replay of Section 6.5 confirms this reading with real data: compressed into the common measured 8-kHz band, band-scaled P5 trails B5 by 0.9–1.0 dB of paired matched-filter peak loss, with the no-CAZAC control attributing the deficit to the chip modulation’s coherence cost under real channel time variability—an operational cost of the CAZAC layer that system designers using its CID/despreading functions must budget for.
*Aggregate-margin caveat:* After the equalized R3/R4a/R4b/D5 sub-scores, the narrow aggregate ordering (Table 11) is driven by R1 ($\sigma_{v}$) and R2 (CID). The aggregate is constructed from rounded per-waveform summaries of the differentiating metrics. Because the retained data do not contain shared trial-level observations for all candidates, a valid joint paired interval is not supported; the score is therefore descriptive (Supplementary S5.13). The equal-aperture control above was applied to resolution/PSL but *not* to the cooperative $\sigma_{v}$/$\sigma_{\tau }$/CID metrics; part of P5’s R1/R3 edge may therefore also reflect its wider aperture rather than the layer architecture. The aggregate ranking is accordingly an indicative summary—the per-column metrics carry the primary evidence—and an equal-aperture cooperative-metric control remains the natural next step.
*Transmit-side non-idealities:* Transducer band-edge rolloff and any TX windowing reduce the effective $\Delta_{BW}$ assumed by Proposition 1; a degradation of $\sigma_{R}$ and $\sigma_{v}$ is expected once realistic transducer responses are inserted, to be quantified in a follow-up sea-trial study.

### 7.4. Reproducibility and Scope

The framework, signal definition, and default parameters of Table 5 support full reproducibility: all results can be regenerated from the simulator and the parameters in Table 5, Table 6, Table 10, and Table 11. Long-range (>10 km) multipath, multi-platform deployment, and a full-knowledge LPI evaluation are planned as follow-up work.

## 8. Conclusions

We presented a functional three-layer decomposition framework for UWA ISAC preambles and its instantiation P5, comprising two spectrally separated chirp layers (HFM and LFM) under a common CAZAC phase-spreading envelope. With the exception of the measured-channel replay of Section 6.5, all performance results reported here are simulation-based and are interpreted within the channel models and attacker assumptions stated in the manuscript. Three propositions, layer orthogonality under $C_{⊥}$, HFM Doppler-invariance, and LFM Rayleigh-like range resolution on the order of $1/B_{L}$, enable simultaneous satisfaction of R1, R3, and R4a ($\sigma_{R}\;\approx \;0.9$ m at 10 dB cooperative-input SNR, multipath-limited and common to all wideband chirp candidates); the mission-critical R4b target ($\sigma_{R}\leq 0.5$ m) is met in benign short-range (quasi-static) geometry. The strict D5 target ($P_{d}\leq 0.1$ at 0 dB ${\mathrm{SNR}}_{\mathrm{att}}$) is met against all four classical structure-aware attackers (CFD, CS, CEP, SFDM). Trained learned attackers are the strongest blind detectors: on the common $\gamma_{E}$ axis, a compact 1-D CNN breaches P5 at +23.0 dB, and a 2-D spectrogram ResNet-18 trained with an online data budget reaches $P_{d}=0.5$ at mean +21.24 dB (10-seed 95% CI [+21.06,+21.42] dB)—4.8 dB below the strongest classical detector and ≈11.2 dB above the full-knowledge oracle. A separate direct 0-dB evaluation gives mean ${\hat{P}}_{d}=0.0014$ over ten seeds for this evaluated protocol. Because these learned detectors exploit the broadband chirp time–frequency signature shared by all chirp candidates, the twelve-seed per-waveform CNN study and the ten-seed ResNet-18 study show crossings for every chirp candidate at comparable attacker-input SNR; within the evaluated ResNet protocol, P5 has the highest mean and a paired +0.49 dB margin over P1 (95% CI [+0.33,+0.65] dB) (Supplementary Tables S3 and S9); we therefore report this as a class-wide limitation of chirp-based LPI rather than a P5-specific weakness, with all learned-attacker breach SNRs read as lower bounds on adversary capability, motivating ML-hardening future work (Section 4.6 and Section 7.3). Across twelve UWA channels (Bellhop, QS, WATERMARK), P5 shows the smallest mean effective-SNR loss and the lowest channel-to-channel variance among the four tested waveforms under the simulator conditions; on band-limited replay through measured NOF1/NCS1 impulse responses, this advantage does not persist (band-scaled P5 trails B5 by 0.816 dB on NOF1 and 0.950 dB on NCS1 in the all-60-sounding analysis). First-eight-sounding controls suggest, but do not establish, a CAZAC coherence mechanism, and the native-band aperture advantage cannot be exercised after compression into the common 8-kHz replay band (Section 6.5). A Pareto search identifies the PSL-optimal set (ρ,N,r)=(0.40,511,37) with PSL =−11.29 dB. The empirical observation that layer orthogonality is necessary but not sufficient for low composite PSL motivates two disjoint design tools: $C_{⊥}$ parameter inequalities and the Pareto search. Two operational limits are equally explicit: even after optimization, the composite autocorrelation PSL (−11.29 dB; −7.60 dB at the balanced default) remains inferior to the equal-aperture SA-HFM baselines (B5 −15.6 dB, P4 −14.0 dB), and sixteen-cell CID collapses to ≤0.07 accuracy under a +2 dB near–far interferer, so near–far-robust multi-cell operation requires per-cell power control or further composite-PSL reduction. All claims are bounded by the stated channel and attacker assumptions.

## Figures and Tables

**Table 1:** Functional requirements for UWA ISAC preambles.

ID	Requirement	Target	Weight
R1	Doppler tracking ($v_{r}\in 0$–15 m/s)	$\sigma_{v}\leq 0.5$ m/s	25%
R2	Cell identification (CID)	≥16 unique IDs	15%
R3	Timing synchronization	$\sigma_{\tau }\leq 2$ sample @ 96 kHz $F_{s}$	20%
R4a	Continuous sensing (ranging)	$\sigma_{R}\leq 1.0$ m @ 10 dB SNR	20%
R4b	Precision ranging (mission-critical)	$\sigma_{R}\leq 0.5$ m in benign short-range/well-separated-arrival (quasi-static) geometry; not claimed for dense multipath	10%
D5	LPI/LPD (4 classical structure-aware attackers *)	$P_{d}\leq 0.1$ @ 0 dB ${\mathrm{SNR}}_{\mathrm{att}}$, $P_{\mathrm{fa}}=10^{-3}$	10%

* CFD, CS, CEP, SFDM. The energy detector (ED) is *excluded* from the D5 target because it is structure-agnostic, its $P_{d}$ depends only on received energy and is identical across waveforms, so ED LPI is reported separately (Section 5.8) as a sanity check, not as part of the D5 scoring. A fully trained CNN attacker is likewise excluded from the per-waveform D5 target: it learns the chirp time–frequency signature common to all chirp-based candidates and is therefore reported as a class-wide limitation (Section 4.6 and Section 7.3), not as a P5-specific discriminator.

**Table 2:** UWA ISAC preamble landscape vs. six functional sub-requirements.

ID	Waveform/Reference	R1 Doppler	R2 CID	R3 Sync	R4a Range	R4b Prec.	D5 LPI	Primary Limitation
B1	HFM single [28]	Y	N	P	N	N	N	No identification, no ranging
B2	Dual HFM up/down [31]	Y	N	P	N	N	N	Doppler sign only; no R2/R4
B3	LFM+ZC [32]	P	P	Y	P	N	P	Doppler degrades under α>1%
B4	Superimposed LFM [13]	P	P	P	P	N	N	Data-centric; no LPI analysis
B5	SA-HFM (reference) [14]	Y	Y	Y	N	N	N	HFM mainlobe wide for ranging
P1	Extended SA-HFM	Y	Y	Y	N	N	N	Same R4 gap as B5
P2	Phase-coded SA-HFM [30]	Y	Y	Y	N	N	P	Gold/Kasami −13 dB floor
P3	HFM+CAZAC	Y	Y	Y	N	N	P	ML-CNN gap; no dedicated range layer
P4	ISAC-enhanced SA-HFM	Y	Y	Y	P	N	N	No LPI treatment
**P5**	**Triple-layer (HFM⊕LFM⊕CAZAC)**	Y	Y ^†^	Y	Y	Cond. ^‡^	Y ^§^	Composite PSL; CNN limit (class-wide)

Y/Cond.(P)/N are *design-intent*, not the 0 dB simulation pass/fail: at 0 dB every chirp candidate passes the four classical detectors alike (Section 5.9). *D5*: Y = constant-modulus codebook co-designed for LPI and CID (P5); P = partial (Gold/Kasami, single-CAZAC); N = none. *R4a* ranks *design* dedication to ranging (dedicated layer + aperture); the realized $\sigma_{R}$ is multipath-limited and uniform (≈0.9 m) across candidates. ^†^ R2 Y = 16-root CAZAC cardinality and balanced-power ID (reverts to P for N≤127/4 roots); perfect in sync/async AWGN, near-far-limited by the common-chirp PSL (Section 5.10). ^‡^ R4b Cond.: the ≤0.5 m target is met in benign short-range (quasi-static) geometry, not by raising SNR. ^§^ The energy detector and the trained learned attackers (1-D CNN, spectrogram ResNet-18) are excluded from D5 as class-wide (structure-agnostic/chirp-class) limitations; the strongest (ResNet-18) reaches $P_{d}=0.5$ at mean +21.24 dB $\gamma_{E}$ (Section 4.6).

**Table 3:** Requirement-to-Layer Mapping.

Req.	Primary Layer	Structural Basis
R1 (Doppler)	HFM	Hyperbolic $f_{\mathrm{inst}}$ absorbs α-scale as τ-shift (Proposition 2)
R2 (CID)	CAZAC	Constant-amplitude zero-autocorrelation; root *r* codebook
R3 (sync)	HFM+CAZAC	Sharp HFM mainlobe + δ-autocorr from CAZAC
R4 (ranging)	LFM	Rectangular spectrum ⇒ $\Delta \tau =1/B_{L}$ (Proposition 3)
D5 (LPI)	Chirp+CAZAC	Chirp time-varying carrier is the primary classical-detector resistance (Section 5.8); CAZAC adds CID and ∼$10\log_{10}\left(N\right)$ despreading vs. a root-blind attacker

**Table 4:** Composite P5 PSD budget at default T=100 ms, N=255.

Chip Envelope	OOB [dB]	Inter-Band [dB]	Δ*C*_⊥_ [dB]	ΔPSL [dB]
Unshaped (default)	−26	−31	≤1.0	0
Raised-cosine, $\beta_{\mathrm{rc}}=0.05$	−34	−39	≤0.6	+0.2
Raised-cosine, $\beta_{\mathrm{rc}}=0.10$	−38	−43	≤0.4	+0.5

PSD values are computed using a zero-padded 65,536-point FFT over the full T=100 ms burst (rectangular analysis window, $F_{s}=96$ kHz) under unit-energy normalization. OOB power is integrated outside the 4–24 kHz mask; “Inter-band” is integrated over the $f_{2}$–$f_{3}=16$–18 kHz gap; both are normalized to the 4–24 kHz in-band power. $\Delta C_{⊥}$ is the shaping-induced change in the realized cross-term relative to the *unshaped* realized value (−32.9 dB), *not* a gap to the idealized bound (6); i.e., chip shaping moves the realized suppression by at most 1 dB. PSL change is likewise relative to the unshaped envelope.

**Table 5:** P5 design parameters (default values).

Parameter	Default	Range
$f_{1}$ (HFM low)	8 kHz	6–10 kHz
$f_{2}$ (HFM high)	16 kHz	14–18 kHz
$f_{3}$ (LFM low)	18 kHz	16–20 kHz
$f_{4}$ (LFM high)	22 kHz	20–24 kHz
*T* (duration)	100 ms	50–200 ms
*N* (ZC length)	255	127/255/511
*r* (ZC root)	29	{r:gcd(r,N)=1}
ρ (split)	0.5	0.2–0.8

**Table 6:** Simulation-based performance and R4 target verification at 10 dB ${\mathrm{SNR}}_{\mathrm{coop}}$ (T=100 ms unless noted).

Parameter	P3	P4	P5^def^	P5^opt^
$\beta_{\mathrm{eff}}$ [kHz] ^†^	16.2	15.0	16.6	15.3
MC RMSE $\sigma_{\tau }$ [μs]	3.1	3.4	3.0	3.3
MC RMSE $\sigma_{v}$ [m/s]	0.35	0.30	0.25	0.28
$\sigma_{R}$ [m] @ 10 dB, T=100 ms ^§^	0.9	0.9	0.9	0.9
R4a target verification ($\sigma_{R}\leq 1.0$ m @ 10 dB SNR):				
R4a satisfied?	yes	yes	yes	yes
R4b target verification ($\sigma_{R}\leq 0.5$ m, mission-critical):				
$\sigma_{R}$, benign short-range geom. ^‡^	≲0.3	≲0.3	≲0.3	≲0.3
R4b satisfied (channel-conditioned)?	yes	yes	yes	yes
Composite PSL [dB]	−6.9	−5.8	−7.60	−11.29

^†^ $\beta_{\mathrm{eff}}$ is a diagnostic back-solved from timing RMSE, not a physical RMS bandwidth. ^§^ Single representative value (≈0.9 m, common to all candidates): at 10 dB, the range error is multipath-limited and channel-set, not waveform-differentiated (exp_sigmaR_reverify.m). ^‡^ R4b (≤0.5 m) is met only in benign short-range (quasi-static) geometry ($\sigma_{R}<0.3$ m, all candidates), not by raising SNR/*T* in dense multipath.

**Table 7:** P5 per-attacker *breach SNR*: the attacker-input ${\mathrm{SNR}}_{\mathrm{att}}$ at which each non-cooperative detector reaches its $P_{d}=0.5$ crossing against P5 (CFAR-calibrated at $P_{\mathrm{fa}}=10^{-3}$, $N_{H_{0}}=10^{4}$, $N_{H_{1}}=10^{3}$; Section 5.8). Lower breach SNR = stronger attacker; every classical entry lies far above the 0 dB D5 operating point. ED is a radiometric sanity check, excluded from D5 scoring.

Attacker	Breach ${\mathrm{SNR}}_{\mathrm{att}}$ @ $P_{d}\;=\;0.5$ [dB] ^†^	Mechanism/Resistance Basis
ED ^§^ (sanity)	≈+31	Radiometer; no structural discrimination
CFD	>+35	Composite cyclic-feature ambiguity
CS	+26.0	Chirp time-varying carrier (strongest classical)
CEP	>+40	Root-*r* randomization breaks periodicity
SFDM	>+35	Squaring blurs carrier estimate
ML-CNN ^‡^	+23.0	*Not suppressed*: learns chirp signature directly (Figure 4)
ML-ResNet ^‡^	+21.24	*Not suppressed*: 2-D spectrogram ResNet-18; 10-seed mean (Supplementary S5.14)

^†^ Attacker-input ${\mathrm{SNR}}_{\mathrm{att}}=E_{s}/N_{0}$ at the empirical $P_{d}=0.5$ crossing (Section 5.1 convention); “>+35”/“>+40” indicate the crossing was not reached within the tested 40 dB sweep. The full-knowledge cooperative/oracle bound, on the same $\gamma_{E}=E_{s}/N_{0}$ axis, lies far lower (+10.0 dB; Table S6). ^§^ ED is excluded from D5 scoring; it detects received energy alone and breaches all chirp waveforms at essentially the same ≈+31 dB. ^‡^ The trained learned attackers (1-D CNN; 2-D spectrogram ResNet-18) are *not* hand-crafted feature detectors and are *not* suppressed by the CAZAC envelope: they breach at +23.0/mean +21.24 dB $\gamma_{E}$, respectively, by learning the chirp signature. The ResNet-18 is the strongest evaluated blind attacker—4.8 dB below CS yet still ≈11.2 dB above the full-knowledge oracle (+10.0 dB)—and both are analyzed as a class-wide limitation in Figure 4, Supplementary S5.14, and Section 7.3; learned-attacker breach SNRs are lower bounds on adversary capability. The CNN and root-bank experiments are parameterized in the per-sample SNR $\gamma_{s}$ and reported here on the common $\gamma_{E}$ axis via $\gamma_{E}=\gamma_{s}+10\log_{10}N_{s}=\gamma_{s}+39.8$ dB ($N_{s}=F_{s}T=9600$).

**Table 8:** Same-protocol baseline optimization (proxy-pareto search).

Waveform	Swept (#)	Config	PSL [dB]	β [kHz]
B5 (SA-HFM)	*M* (4)	M=3 (def/opt)	−7.08	14.11
P3 (HFM-CAZAC)	(N,r) (15)	N=255 (def)	−5.09	11.98
		N=511 (opt)	−5.16	12.61
P4 (ISAC-SA-HFM)	(M,ρ) (20)	M=4,ρ=0.5 (def/opt)	−6.09	14.48

Proxy-Pareto search of Section 5.3 applied to each competitor’s free parameters under a fixed 8–16 kHz band; the PSL column is an in-band ranking proxy, not directly comparable to the native-allocation PSL of Table 6. The number in parentheses in the “Swept (#)” column is the number of parameter configurations evaluated in that sweep. “def/opt” marks a default already at the proxy-optimum.

**Table 9:** Layer-by-layer ablation of P5 (default parameters).

Variant	PSL [dB]	ml [ms]	$\beta_{\mathrm{pb}}$ [kHz]
HFM only	−4.94	0.115	11.3
LFM only	−3.92	0.219	20.0
ZC only	−3.35	0.219	4.2
HFM+LFM (no ZC)	−5.22	0.052	16.3
Full P5	−7.60	0.052	16.7

$\beta_{\mathrm{pb}}$ is the *passband* (absolute, non-central) RMS bandwidth, used here only for relative ablation ranking; it is distinct from the central-moment β of (9) and from the diagnostic $\beta_{\mathrm{eff}}$ of Table 6 (e.g., LFM-only $\beta_{\mathrm{pb}}=20.0$ kHz vs. the central-moment $4/\;\sqrt{12}=1.155$ kHz).

**Table 10:** P5 Mission-reconfigurable parameter sets (simulated).

Mission	(N,ρ)	PSL [dB]	ml [ms]	β [kHz]
M1 AUV swarm	(255,0.5)	−7.60	0.052	16.7
M2 Sub-UUV	(511,0.4)	−11.06	0.052	16.2
M3 Ship-UUV	(255,0.5)	−7.62	0.052	16.7
M4 Mine-ISAC	(127,0.6)	−5.66	0.052	17.4

**Table 11:** Quantitative simulation comparison across the ten waveform families (eleven rows) and six sub-requirements (R1/R2/R3/R4a/R4b/D5), weighted by Table 1.

Waveform	$\sigma_{v}$ [m/s]	CID Acc.	$\sigma_{\tau }$ [μs]	$\sigma_{R}$ [m]	D5 $P_{d}$ @ 0 dB ${\mathrm{SNR}}_{\mathrm{att}}$ *	Aggregate
B1 (HFM single)	0.50	0	4.1	0.9	<0.01	0.65
B2 (dual HFM)	0.48	0	4.0	0.9	<0.01	0.66
B3 (LFM+ZC)	0.80	0.85	3.7	0.9	<0.01	0.70
B4 (sup-LFM)	0.70	0	3.8	0.9	<0.01	0.60
B5 (SA-HFM)	0.31	0.95	3.4	0.9	<0.01	0.88
P1 (ext SA-HFM)	0.31	0.95	3.4	0.9	<0.01	0.88
P2 (phase-coded SA-HFM)	0.32	0.90	3.4	0.9	<0.01	0.87
P3 (HFM+CAZAC)	0.35	0.96	3.1	0.9	<0.01	0.86
P4 (ISAC-SA-HFM)	0.30	0.95	3.4	0.9	<0.01	0.89
P5^def^ (0.5,255,29)	0.25	0.97	3.0	0.9	<0.01	0.92
P5^opt^ (0.40,511,37)	0.28	0.97	3.3	0.9	<0.01	0.90

* Worst-case ${\hat{P}}_{d}$ across the four *classical* structure-aware attackers (CFD, CS, CEP, SFDM) at 0 dB ${\mathrm{SNR}}_{\mathrm{att}}$ ($N_{H_{0}}=10^{4}$/$N_{H_{1}}=10^{3}$ MC per cell, CFAR $P_{\mathrm{fa}}=10^{-3}$). At 0 dB, ${\hat{P}}_{d}$ is at the $P_{\mathrm{fa}}$ floor for *all* candidates (D5 met class-wide); waveforms differ only in the higher-SNR breach regime (Figure 7). The energy detector and trained CNN are excluded as class-wide limitations. The CID column is the *balanced-power* 4-cell value (R2b); near-far 16-cell CID degrades sharply (Section 5.10, Supplementary Table S4). The $\sigma_{R}$ column is the single multipath-limited value ≈ 0.9 m (channel-set; per-waveform spread 0.83–0.94 m within MC variability), so all candidates share the R4a sub-score and the aggregate ordering is driven by R1 (Doppler) and R2 (CID). The aggregate is a deterministic score computed from rounded point estimates; no sampling interval is reported because the shared trial-level records needed for paired inference are unavailable (Supplementary S5.13).

**Table 12:** Measured single-thread runtime per detection buffer (i9-13900K reference; median [IQR]; randomized interleaved timing). Receiver-stage and CID ratios use their own one-correlation implementations as baselines; the CID rows execute every root serially.

Stage	Correlations	Median [IQR] [ms]	Within-Stage Ratio
1-D delay-only MF	1	0.501 [0.325]	1×
2-D WBAF, full grid	21	8.440 [2.375]	16.85×
2-D WBAF, coarse-to-fine	12	3.421 [1.211]	6.83×
CID root scan, 1 cell	1	2.070 [0.450]	1×
CID root scan, 16 cells	16	34.317 [2.967]	16.58×
CID root scan, 64 cells	64	136.815 [6.715]	66.11×

**Table 13:** Twelve UWA channel environments.

Channel	Type	Range	Key Parameter
Bellhop-ShallowCalm	geom.	500 m	Beaufort 1, summer SVP
Bellhop-ShallowRough	geom.	500 m	Beaufort 5, summer SVP
Bellhop-DeepWinter	geom.	2 km	Beaufort 3, winter SVP
Bellhop-DeepSummer	geom.	2 km	Beaufort 3, summer SVP
Bellhop-BayProtected	geom.	300 m	Beaufort 0, sand
QS-LowFading	stat.	500 m	$\sigma_{L}\;=\;0.2$, 1 m/s
QS-MidFading	stat.	1 km	$\sigma_{L}\;=\;0.5$, 2 m/s
QS-HighFading	stat.	2 km	$\sigma_{L}\;=\;1.0$, 3 m/s
WMK-NOF1	surrog.	1 km	Norway fjord
WMK-NCS1	surrog.	1 km	Norway cont. shelf
WMK-KAU1	surrog.	1 km	Kauai shallow
WMK-KAU2	surrog.	2 km	Kauai deeper

**Table 14:** Aggregate $L_{\mathrm{eff}}$ across twelve UWA channels.

Waveform	Mean [dB]	std [dB]	Worst [dB]	Best [dB]
B5	−0.62	0.74	−1.55	+0.66
P3	−0.40	0.77	−1.38	+0.89
P4	−0.56	0.73	−1.46	+0.65
P5	−0.35	0.67	−1.16	+0.80

**Table 15:** Measured-channel replay (WATERMARK, band-scaled variants): mean ± SD across 60 sounding means of the matched-filter peak loss $L_{\mathrm{eff}}$ [dB], and paired P5^def^ differences with sounding-cluster 95% bootstrap CIs.

	NOF1 (60 Soundings)	NCS1 (60 Soundings)
B5 (SA-HFM)	−4.389 ± 1.382	−7.231 ± 0.313
P4 (ISAC-SA-HFM)	− 4.498 ± 1.347	− 7.343 ± 0.324
P5^def^ (*N* = 127)	−5.205 ± 1.286	−8.181 ± 0.329
P5^opt^ (*N* = 127)	−5.081 ± 1.278	−8.100 ± 0.316
Δ(P5^def^ − B5), paired	−0.816 [−1.021, −0.621]	−0.950 [−0.977, −0.923]
Δ(P5^def^ − P4), paired	−0.707 [−0.887, −0.535]	−0.839 [−0.863, −0.815]

## Data Availability

The data presented in this study—the Preamble Design Toolbox (MATLAB R2024b/Python 3.12.3), the waveform and channel parameter files, the Monte Carlo random-number seeds, the trained CNN weights, and the raw detection/ROC counts underlying the reported figures and tables—are available from the corresponding author upon reasonable request.

## Supplementary Materials

The following supporting information can be downloaded at: https://www.mdpi.com/article/10.3390/s26154814/s1, detailed proofs of Propositions 1–3 (layer orthogonality, HFM Doppler-invariance, and LFM range resolution), the full Cramér–Rao lower bound (CRLB) derivation, and the extended verification and ablation experiments S5.1–S5.15 (Tables S1–S10, Figures S1–S9), including the aggregate-score audit (S5.13), the spectrogram ResNet-18 learned-attacker study (S5.14), and the measured-channel replay statistics (S5.15) added in revision.

## Abbreviations

The following abbreviations are used in this manuscript:

CAZAC	Constant-amplitude zero-autocorrelation
ISAC	Integrated sensing and communication
HFM	Hyperbolic frequency modulation
LFM	Linear frequency modulation
LPI	Low probability of intercept
CNN	Convolutional neural network
PAPR	Peak-to-average power ratio
CFAR	Constant false-alarm rate
AUV	Autonomous underwater vehicle
ZC	Zadoff–Chu
UWA	Underwater acoustic
PSL	Peak-sidelobe level
CID	Cell identification
SNR	Signal-to-noise ratio
RMSE	Root mean square error
CRLB	Cramér–Rao lower bound
WBAF	Wideband ambiguity function
UUV	Unmanned underwater vehicle
