# Supplementary material for: A Triple-Layer HFM–LFM–CAZAC Preamble Framework for Underwater Acoustic Integrated Sensing and Communication
Source: Sensors (Basel). 2026 Jul 29;26(15):4814. doi: 10.3390/s26154814 (PMC13469405; doi:10.3390/s26154814)
Supplement: Supplementary file 1 [file sensors-26-04814-s001.zip › sensors-4432869-supplementary.pdf]

Supplementary Materials

A Triple-Layer HFM–LFM–CAZAC Preamble Framework for Underwater Acoustic Integrated Sensing and Communication

Seunggyu Kim, Saeyong Park, and Taeho Im

This document provides the proofs of Propositions 1–3 and the full Cramér–Rao lower bound (CRLB) derivation referenced in the main manuscript. Notation follows the main manuscript: Φ_{HFM} and Φ_{LFM} are the HFM and LFM phases of the composite preamble s_{P5}, $z(t) = e^{j\phi_{ZC}(t)}$ is the constant-modulus CAZAC envelope ($|z(t)| = 1$), $\mathcal{C}_\perp$ is the orthogonality region, $\Delta_{BW} = f_3 - f_2$ is the inter-band gap, $B_H = f_2 - f_1$ and $B_L = f_4 - f_3$ are the HFM and LFM bandwidths, and $\widetilde{\chi}_{XY}(\tau, \alpha)$ denotes the ZC-weighted cross-ambiguity functions.

S1. Proof of Proposition 1 (Orthogonality of Layers)

We establish, via non-stationary-phase (integration-by-parts) analysis over the disjoint frequency supports of the HFM and LFM layers, the cross-term bound

$$\frac{\max_{\tau,\alpha} |\widetilde{\chi}_{HL}(\tau, \alpha)|}{\max |\widetilde{\chi}_{HH}|} \le \frac{\sqrt{\alpha_{\max}}}{\pi(\Delta_{BW} - \eta f_3)T} \approx \frac{1}{2\pi\Delta_{BW}T},$$

the right-hand form being the leading-order estimate ($\sqrt{\alpha_{\max}} \to 1$, $\eta f_3 \ll \Delta_{BW}$); the rigorous constant is derived in S1.4.

S1.1 Setup

Let $h(t) = e^{j2\pi\Phi_{HFM}(t)}$ with instantaneous frequency $f_{HFM}(t) = 1/[1/f_1 + (1/f_2 - 1/f_1)t/T] \in [f_1, f_2]$ and $\ell(t) = e^{j2\pi\Phi_{LFM}(t)}$ with $f_{LFM}(t) = f_3 + (f_4 - f_3)t/T \in [f_3, f_4]$. Under $\mathcal{C}_\perp$, $f_3 > f_2$, so the two instantaneous-frequency tracks never coincide: $f_{LFM}(t) - f_{HFM}(t) \ge \Delta_{BW} > 0$ for all $t \in [0, T]$.

The ZC-weighted cross-ambiguity function is

$$\widetilde{\chi}_{HL}(\tau, \alpha) = \sqrt{\alpha} \int_0^{T/\alpha} K_Z(t, \tau, \alpha)\, h(t)\, \ell^*(\alpha(t - \tau))\, dt, \tag{S1}$$

where $K_Z(t, \tau, \alpha) = z(t)\, z^*(\alpha(t - \tau))$ satisfies $|K_Z| = 1$ pointwise because $|z(t)| = 1$ (the CAZAC envelope has constant amplitude).

S1.2 Phase Derivative Lower Bound

Writing the integrand of (S1) as $K_Z \cdot e^{j\phi(t)}$ with chirp phase $\phi(t) = 2\pi[\Phi_{HFM}(t) - \Phi_{LFM}(\alpha(t - \tau))]$, the derivative is $\dot{\phi}(t) = 2\pi[f_{HFM}(t) - \alpha f_{LFM}(\alpha(t - \tau))]$. For the UWA Doppler regime $\alpha \in [1-\eta, 1+\eta]$,

$$|\dot{\phi}(t)| \ge 2\pi\big[(1-\eta)f_3 - f_2\big] = 2\pi\big(\Delta_{BW} - \eta f_3\big), \tag{S2}$$

which is strictly positive, ruling out a stationary point, provided the Doppler-dependent condition $\Delta_{BW} > \eta f_3$ holds; we therefore add this condition to $\mathcal{C}_\perp$. (At the full R1 Doppler extent $\eta = 2 \times 10^{-2}$ and $f_3 = 18$ kHz the threshold is $\eta f_3 = 360$ Hz, comfortably below $\Delta_{BW} = 2$ kHz.)

S1.3 Non-Stationary-Phase Bound

By the first-derivative (van der Corput) test: if $\dot{\phi}$ is *monotonic* with $|\dot{\phi}| \geq \mu > 0$ on an interval and g has bounded variation $V(g)$ with $\|g\|_\infty \leq M$, then $|\int g\, e^{j\phi}\, dt| \leq \mu^{-1}\big(2M + V(g)\big)$, the $2M$ collecting the two endpoint terms and $V(g)$ the interior variation. Under $\mathcal{C}_\perp$ the phase derivative $\dot{\phi} = 2\pi[f_{HFM}(t) - \alpha f_{LFM}(\alpha(t-\tau))]$ satisfies $|\dot{\phi}| \geq \mu = 2\pi(\Delta_{BW} - \eta f_3) > 0$ by S1.2 (no stationary point), but it is *not* globally monotonic on $[0,T]$: its derivative $\ddot{\phi} = 2\pi[\dot{f}_{HFM}(t) - \alpha^2 \dot{f}_{LFM}]$ pits the monotonically increasing $\dot{f}_{HFM}(t)$ against the constant $\alpha^2 B_L/T$, so $\ddot{\phi}$ changes sign at most once and $\dot{\phi}$ has at most one interior extremum. We therefore split $[0,T]$ into the (at most two) subintervals on which $\dot{\phi}$ is monotonic, apply the test on each, and sum; this inflates the constant by a factor ≤ 2, absorbed into the $O(1)$ prefactor below. In the *smooth-envelope idealization* the envelope is taken in its slowly-varying (uncoded) limit, so $g = \sqrt{\alpha} K_Z$ has negligible variation ($V(g) \approx 0$) and the bound reduces to $2M/\mu$. This is an idealization, *not* a property of the realized unit-modulus chip code, whose total variation grows as $O(N)$ with the chip count despite $|K_Z| = 1$ and is precisely the chip-leakage term quantified below. Applying the bound to (S1) with $g = \sqrt{\alpha} K_Z$ ($M = \sqrt{\alpha_{\max}}$) and $\mu = 2\pi(\Delta_{BW} - \eta f_3)$,

$$|\widetilde{\chi}_{HL}(\tau, \alpha)| \leq \frac{\sqrt{\alpha_{\max}}}{\pi(\Delta_{BW} - \eta f_3)}. \tag{S3}$$

S1.4 Normalization and Result

For unit-amplitude chirp segments on $[0,T]$ the autocorrelation peak satisfies $\max|\widetilde{\chi}_{HH}| = T$. Dividing (S3) by $\max|\widetilde{\chi}_{HH}|$ gives the *smooth-envelope ideal lemma*

$$\frac{|\widetilde{\chi}_{HL}|}{\max|\widetilde{\chi}_{HH}|} \leq \frac{\sqrt{\alpha_{\max}}}{\pi(\Delta_{BW} - \eta f_3)\, T}. \tag{S4}$$

At default parameters ($\Delta_{BW} = 2$ kHz, $\eta f_3 = 360$ Hz, $T = 0.1$ s) this evaluates to about -54 dB (the leading-term estimate $1/(2\pi\Delta_{BW}T)$ quoted in the main manuscript gives -62 dB; the two differ only by the $O(1)$ prefactor). This lemma bounds only the smooth-amplitude idealization; the realized waveform is governed by the chip-discontinuity correction below.

Chip-discontinuity correction. The integration-by-parts step of S1.3 requires the amplitude $g = \sqrt{\alpha} K_Z$ to be of bounded variation; the actual CAZAC kernel $K_Z(t) = z(t) z^*(\alpha(t-\tau))$ is piecewise-constant with $N-1$ phase jumps at the chip boundaries $\{kT_c\}$. Each jump contributes an un-cancelled boundary term, and the associated spectral leakage has width $\sim 1/T_c = N/T$. When $1/T_c \gtrsim \Delta_{BW}$ this leakage spans the inter-band gap, so (S4) holds only for the smooth component and *understates* the realized cross-term. Including the chip-edge contributions, the bound degrades to

$$\frac{|\widetilde{\chi}_{HL}|}{\max|\widetilde{\chi}_{HH}|} \lesssim \frac{1}{2\pi\Delta_{BW}T} + \frac{c_0\,(N/T)}{B_H + B_L} \cdot \frac{1}{\Delta_{BW}T}, \tag{S5}$$

with c_0 an envelope-shape constant calibrated against the numerics ($c_0 \approx 20$ at the defaults), so (S5) is an *empirical* design relation rather than an a priori bound, and the operative figure is the directly measured cross-ambiguity. At the defaults ($1/T_c = 2.55$ kHz $> \Delta_{BW} = 2$ kHz) the second term dominates; a direct numerical evaluation of the full delay–Doppler cross-ambiguity of the actual envelope-modulated layers gives -32.9 dB (HFM-LFM), confirming that the realized suppression, while ~ 22–29 dB above the idealized smooth-envelope estimate (-55 to -62 dB), still clears the -20 dB orthogonality threshold by ≥ 12 dB (consistent with the $\mathcal{C}_\perp$ discussion in the main manuscript).

S1.5 Extension to HFM-ZC and LFM-ZC

Because the CAZAC factor is *multiplicative*, $\widetilde{\chi}_{HZ}$ and $\widetilde{\chi}_{LZ}$ are *not* additive cross-terms of χ_{P5} (which contains only the HH, LL, HL, and LH terms of the main text); they are envelope-vs-chirp diagnostics, measured to confirm that the CAZAC spreading does not reshape the chirp ridges. With that understood, the same lemma applied chip-by-chip on the CAZAC partition $\{[kT_c,(k+1)T_c]\}_{k=0}^{N-1}$, combined with $\sqrt{N}$ cancellation from the CAZAC zero-autocorrelation phases, yields $|\widetilde{\chi}_{HZ}|/\max|\widetilde{\chi}_{HH}| \lesssim \sqrt{N}/(\pi B_H T)$ and analogously for $\widetilde{\chi}_{LZ}$ with $B_H \to B_L$. For defaults these evaluate to −44 dB and −38 dB respectively. Direct numerical evaluation of the full delay–Doppler cross-ambiguity for the actual envelope-modulated layers gives −35.5 dB ($\widetilde{\chi}_{HZ}$) and −42.0 dB ($\widetilde{\chi}_{LZ}$); the HFM-ZC term is slightly above its chip-by-chip estimate (the same chip-leakage effect of (S5)), while both stay $\geq$ 15 dB below the −20 dB orthogonality threshold. ■

S2. Proof of Proposition 2 (Doppler-Invariance Preservation)

The HFM phase is $\Phi_{HFM}(t) = \int_0^t f_{HFM}(\tau)\,d\tau$ with $f_{HFM}(\tau) = 1/(a + b\tau/T)$, where $a = 1/f_1$ and $b = 1/f_2 - 1/f_1$. Integrating,

$$\Phi_{HFM}(t) = \frac{T}{b}\left[\ln(a + bt/T) - \ln a\right]. \tag{S6}$$

A Doppler scale α acts as $t \mapsto \alpha t$. Substituting and seeking a time shift τ_D and constant phase ψ_0 such that $\Phi_{HFM}(\alpha t) = \Phi_{HFM}(t - \tau_D) + \psi_0/(2\pi)$, we match the affine argument of the logarithm in (S6): $a + b\alpha t/T = \kappa\,[a + b(t - \tau_D)/T]$. Equating the t-coefficient gives $\kappa = \alpha$, and equating the constant term gives $a = \alpha(a - b\tau_D/T)$, hence

$$\tau_D(\alpha) = \frac{aT}{b}\left(1 - \tfrac{1}{\alpha}\right), \qquad \psi_0(\alpha) = 2\pi\,\frac{T}{b}\,\ln\alpha. \tag{S7}$$

Therefore $\Phi_{HFM}(\alpha t) = \Phi_{HFM}(t - \tau_D(\alpha)) + (T/b)\ln\alpha$, equivalently a constant phase $\psi_0(\alpha) = 2\pi(T/b)\ln\alpha$ added to $2\pi\Phi_{HFM}$: a wideband Doppler scaling of the *ideal* hyperbolic phase is absorbed exactly into a delay plus a constant phase, whereas linear (LFM) chirps admit no such identity. The identity is exact only for the infinite-support, unmodulated HFM phase. The finite rectangular support $[0, T]$, the multiplicative CAZAC envelope $z(t)$ (whose chip grid is itself rescaled to $z(\alpha t)$), and the additive LFM component are *not* covered by it; for the actual P5 waveform the near-invariance of the HFM ridge is therefore a numerical observation (Figure 8 of the main manuscript), not a corollary of this identity alone. ■

S3. Proof of Proposition 3 (Range Resolution)

Proposition 3 concerns the *despread LFM branch* of the cooperative ranging receiver, which knows the CAZAC root and correlates against the envelope-modulated LFM template $z\,\ell$ (equivalently, multiplies the input by z^* to remove the envelope before the LFM matched filter). The ZC-weighted auto-term at zero Doppler is $\widetilde{\chi}_{LL}(\tau, 1) = \int z(t)z^*(t-\tau)\,\ell(t)\ell^*(t-\tau)\,dt$. The plain-LFM stationary-phase approximation gives $|\widetilde{\chi}_{LL}(\tau, 1)| \propto |\mathrm{sinc}(B_L\tau)|$ with first null at $\Delta\tau_{\mathrm{Ray}} = 1/B_L$. This is, however, only a *conservative* (bandwidth-only) reference: the CAZAC factor $z(t)z^*(t-\tau)$ is *not* negligible over the LFM mainlobe (τ/T_c reaches ≈ 0.64 at the first null), and the chip modulation (chip rate $1/T_c = 2.55$ kHz) broadens the despread branch spectrum from $B_L \approx 4$ to ≈ 6.5 kHz, so the realized $\widetilde{\chi}_{LL}$ is in fact *narrower* than the plain-LFM sinc—a measured −3 dB mainlobe of ≈ 0.146 ms vs. $0.886/B_L = 0.221$ ms (main-text Section 5.6). This despread LFM-branch resolution is distinct from the *full-composite* autocorrelation, whose

still-narrower mainlobe is set by the entire f_1–f_4 spectral aperture (main-text ablation). By the two-way propagation relation $R = c\tau/2$, the corresponding (conservative) two-target range resolution is

$$\Delta R \approx \frac{c\,\Delta\tau_{\text{Ray}}}{2} = \frac{c}{2B_L}. \tag{S8}$$

The -3 dB full mainlobe width of a sinc is $\Delta\tau_{-3\text{dB}} \approx 0.886/B_L$, about 11% narrower than the first-null width. For the default $B_L = 4$ kHz, (S8) gives $\Delta R = 18.75$ cm. This is the two-target *resolution floor* and is distinct from the single-target ranging *precision* σ_R bounded in S4. ■

S4. Cramér–Rao Lower Bound Derivation

For the complex baseband P5 signal in additive white Gaussian noise with two-sided PSD $N_0/2$, the Fisher Information Matrix (FIM) [1] is formed for the *independent* parameters (τ, α) with the complex amplitude as a nuisance parameter, $\mathbf{I}_{ij} = (2/N_0)\,\Re\big[\int(\partial_i s)^*(\partial_j s)\,dt\big]$. The range is the *derived* quantity $R = c\tau/2$ under a monostatic two-way model, so its bound follows by the transformation $\text{var}(\hat{R}) = (c/2)^2\,\text{var}(\hat{\tau})$; we do *not* treat τ and R as jointly independent parameters, which would render the FIM singular.

Scope of the CRLB derivation. The relations below are obtained under the idealized assumptions of (i) known waveform with *unknown* complex amplitude (its magnitude and phase treated as nuisance parameters, consistently with the central/unknown-phase bandwidth convention used throughout), (ii) AWGN-only channel (no multipath, no fading), and (iii) unbiased estimation. They are therefore *scaling references* that an arbitrary efficient estimator could approach asymptotically; they are not directly comparable to the matched-filter Monte Carlo RMSEs reported in the main manuscript, which are produced by a single-pass composite matched filter operating on a Bellhop multipath channel. We retain the ideal CRLB as an interpretive reference for the *scaling* (with bandwidth, SNR, and observation time) of the empirical RMSE rather than as a numerical target.

S4.1 Bandwidth Conventions

Two distinct "bandwidth" quantities appear in the CRLB literature for chirp signals, and conflating them is a frequent source of order-of-magnitude error. *(i) Baseband / central-second-moment RMS bandwidth.* For a chirp whose energy is uniformly distributed on $[f_a, f_b]$ (rectangular spectrum approximation), the central second moment about the spectral mean $\bar{f} = (f_a + f_b)/2$ is $\beta^2_{\text{bb},X} = (f_b - f_a)^2/12$. This is the quantity that appears in delay/range CRLB expressions written for baseband envelopes, and is invariant to absolute carrier frequency. *(ii) Passband-absolute second moment.* $\beta^2_{\text{pb},X} = (f_a^2 + f_a f_b + f_b^2)/3$ is the non-central second moment about $f = 0$. It includes the absolute carrier offset and overstates the achievable timing precision for narrowband passband waveforms; it should *not* be substituted into the baseband CRLB form. For the default LFM band $[f_3, f_4] = [18, 22]$ kHz these conventions give $\beta_{\text{bb},L} = 4/\sqrt{12} \approx 1.155$ kHz and $\beta_{\text{pb},L} \approx 20$ kHz respectively, a factor of ~ 17 apart. With *known* carrier phase the delay information formally involves the non-central (passband) second moment, whereas the central-moment form used in eq. (9) of the main manuscript corresponds to the unknown-phase case with phase removed as a nuisance parameter; the two differ by more than an order of magnitude. We therefore do not use the analytical β as a numerical target: the diagnostic β_{eff} of the main manuscript is *back-solved* from the empirical timing RMSE, and the ideal CRLB is retained only as a loose scaling reference.

S4.2 Timing and Range

Standard Parseval analysis gives the (ideal-AWGN) timing CRLB

$$\mathrm{var}(\hat{\tau}) \geq \frac{1}{(2\pi)^2 \beta^2_{\mathrm{bb},P5}\,\mathrm{SNR}},$$

where the band-disjoint condition $f_2 < f_3$ allows the composite baseband RMS bandwidth to be written as $\beta^2_{\mathrm{bb},P5} = A_H^2\,\beta^2_{\mathrm{bb},H} + A_L^2\,\beta^2_{\mathrm{bb},L} + A_H^2 A_L^2 (\bar{f}_H - \bar{f}_L)^2$, where the last term captures the inter-band offset between the HFM and LFM allocations and is the dominant contribution at the default parameters. For the LFM-only ranging bound on a baseband-equivalent channel,

$$\mathrm{var}(\hat{R}) \geq \frac{c^2}{4(2\pi)^2 A_L^2 \beta^2_{\mathrm{bb},L}\,\mathrm{SNR}}.$$

With $c = 1500$ m/s, $A_L^2 = 0.5$, $\beta_{\mathrm{bb},L} \approx 1.155$ kHz, and SNR $= 10$ dB (linear ratio 10), this ideal bound evaluates to $\sigma_R^{\mathrm{CRLB,ideal}} \approx 4.6$ cm. The empirical matched-filter RMSE $\sigma_R^{\mathrm{MC}} \approx 0.9$ m reported in the main manuscript is approximately $20\times$ above this ideal CRLB; the gap reflects (a) the single-pass composite matched filter rather than an LFM-only sufficient statistic, (b) multipath leaking energy out of the main correlation peak—the *dominant* term: the AWGN-only jitter floor is sub-millimetre, so σ_R is multipath-limited and common across all wideband chirp candidates, (c) grid-quantization and interpolation losses in the peak-search stage, and (d) the absence of a joint phase/Doppler tracking loop. We therefore present the ideal CRLB only as a scaling reference and use the empirical RMSE for all R4a/R4b verification statements. (Applying instead the central-moment β of eq. (9) to the timing CRLB would give $\sigma_\tau \approx 11$ μs, larger than the empirical 3.0 μs, confirming that the matched filter exploits the full passband rather than the central-moment bandwidth and that eq. (9) is not the operative bandwidth.)

S4.3 Doppler and Off-Diagonal Coupling

The Doppler-scale FIM element evaluates to $I_{\alpha\alpha} \approx (2\pi)^2 T^3 \beta^2_{\mathrm{bb},P5}/(3N_0)$, giving an order-of-magnitude ideal bound $\sigma_\alpha \gtrsim \mathcal{O}(10^{-4})$ at 10 dB SNR; the empirical Doppler RMSE $\sigma_v = 0.25$ m/s reported in the main manuscript is the matched-filter Monte Carlo result and is the value used to verify R1. A numerical FIM computed directly from the sampled P5 waveform for $\boldsymbol{\theta} = [\Re a, \Im a, \tau, \alpha]$ (the complex channel gain as a nuisance) clarifies how the ideal bounds relate to the empirical RMSE. The raw condition number is unit-dependent ($\kappa(\mathbf{I}) \approx 8.0\times 10^{10}$ with τ in seconds); we therefore report the scale-invariant condition number of the FIM *correlation* matrix, $\kappa_{\mathrm{corr}} \approx 84$, which still exposes a strong delay–Doppler coupling ($|\rho_{\tau\alpha}| \approx 0.88$) that Proposition 1 does *not* bound (it constrains only the inter-layer HFM–LFM cross-terms, whereas the LFM layer has intrinsic delay–Doppler coupling). Three timing bounds follow, all removing the amplitude nuisance by Schur complement: (i) the *joint* (τ, α) bound $\sigma_\tau^{\mathrm{joint}} \approx 12.2$ μs ($\sigma_\alpha \approx 1.0 \times 10^{-4}$, $\sigma_v \approx 0.075$ m/s); (ii) the α-known timing bound with amplitude *and carrier phase* as nuisance, $\sigma_\tau^{\alpha\text{-known}} \approx 10.0$ μs; and (iii) the fully phase-coherent (amplitude and phase known) bound $\sigma_\tau^{\mathrm{coh}} = 1/\sqrt{I_{\tau\tau}} \approx 2.84$ μs. The empirical sync RMSE (3.0 μs) matches (iii), *not* (i)–(ii): the cooperative receiver is coherent—it estimates and compensates the channel gain from the same high-TBP preamble, so the magnitude-peak timing is effectively gain-invariant and approaches the phase-coherent (passband-bandwidth) bound, whereas (i)–(ii) are the looser unknown-phase bounds governed by the smaller central-moment bandwidth of Eq. (9) in the main text. The receiver moreover *searches* $\hat{\alpha}$ (it does not know α); for the direct-path sync at $\alpha \approx 1$ the residual (τ, α) coupling is negligible. We present (i)–(iii) as scaling references that *bracket* the empirical RMSE rather than tight operational bounds, consistent with the bandwidth-scaling reading of § 4.5; R4a/R4b are verified by the empirical RMSE throughout. The per-attacker detector-statistic re-derivation referenced in the main text remains deferred.

S5. Verification and Ablation Experiments

This section collects the verification and ablation experiments referenced from Section 5 of the main manuscript. All use the default P5 parameters unless noted; the scripts and random seeds are included in the revision analysis package and are available from the corresponding author on request.

S5.1 Ranging-receiver branches and two-target resolution

Three cooperative ranging receivers are evaluated on the *same* P5 target echo (AWGN, 10 dB, monostatic single point target): the composite matched filter (template $z(A_H h + A_L \ell)$), the despread LFM branch (template $z\ell$, the ZC-weighted auto-term $\widetilde{\chi}_{LL}$), and the HFM branch (template zh); Figure S1 shows their autocorrelation profiles and two-target probability of resolution. (i) The composite mainlobe is narrow (-3 dB width 0.063 ms) but its PSL is high (-7.60 dB); the despread LFM branch is wider (0.146 ms) with cleaner sidelobes (-11.7 dB). (ii) The realized $\widetilde{\chi}_{LL}$ is *narrower* than the plain-LFM Rayleigh value $0.886/B_L = 0.221$ ms, because the CAZAC chip modulation (chip rate $1/T_c = 2.55$ kHz) broadens the despread branch spectrum to ≈ 6.5 kHz; the closed-form $\Delta R = c/2B_L = 18.75$ cm is therefore a conservative reference. (iii) The two-target probability of resolution reaches 0.5 at ≈ 0.23 m for *both* receivers: the composite's narrow mainlobe does not resolve closer targets because its -7.6 dB sidelobes fill the trough (the aperture/grating trade-off). In AWGN the single-target range RMSE is submillimetre at 10 dB, confirming that the $\sigma_R \approx 0.9$ m of the main text is set by multipath, not the resolution floor (and is therefore multipath-limited and common across all wideband chirp candidates).

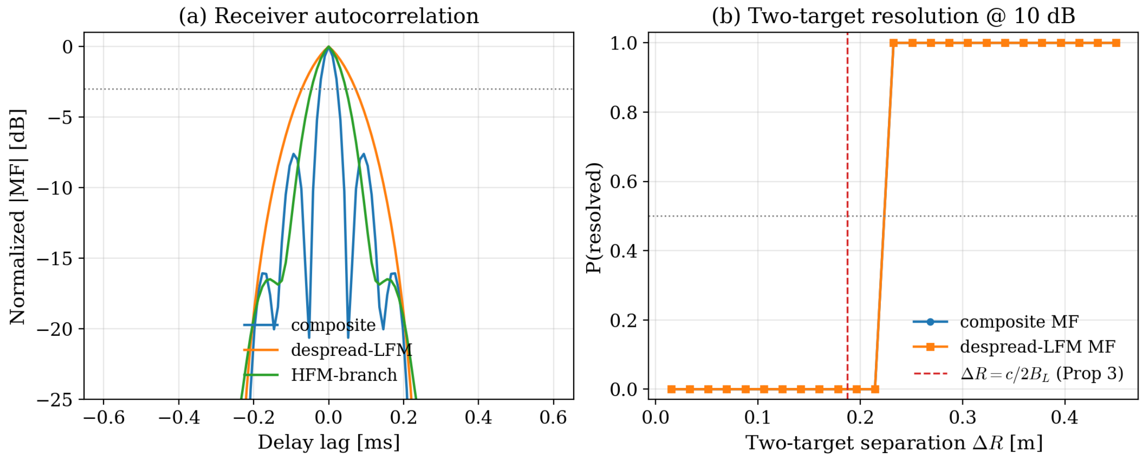

Figure S1: Proposition 3 receiver branches (AWGN, 10 dB): (a) composite, despread-LFM, and HFM-branch autocorrelation; (b) two-target probability of resolution (dashed: plain-LFM $\Delta R = c/2B_L$).

S5.2 Doppler-invariance ablation

To isolate the HFM-intrinsic Doppler invariance from the receiver search gain, HFM-only, LFM-only, HFM+LFM, and full P5 are evaluated under both a 1-D delay-only and a 2-D Doppler-compensated filter (Figure S2). Under the *1-D* filter at 2% Doppler, HFM-only loses only 0.37 dB—its hyperbolic phase absorbs the scale as a delay shift—whereas LFM-only loses 10.4 dB, HFM+LFM 6.5 dB, and full P5 16.6 dB (the multiplicative CAZAC grid $z(\alpha t)$ and the LFM component are not Doppler-invariant). Under the *2-D* filter all four recover to ≤ 0.05 dB.

The HFM layer thus carries a genuine intrinsic Doppler invariance, but the full P5 relies on the receiver Doppler search; Figure 8 of the main text measures the receiver, not an HFM-specific property.

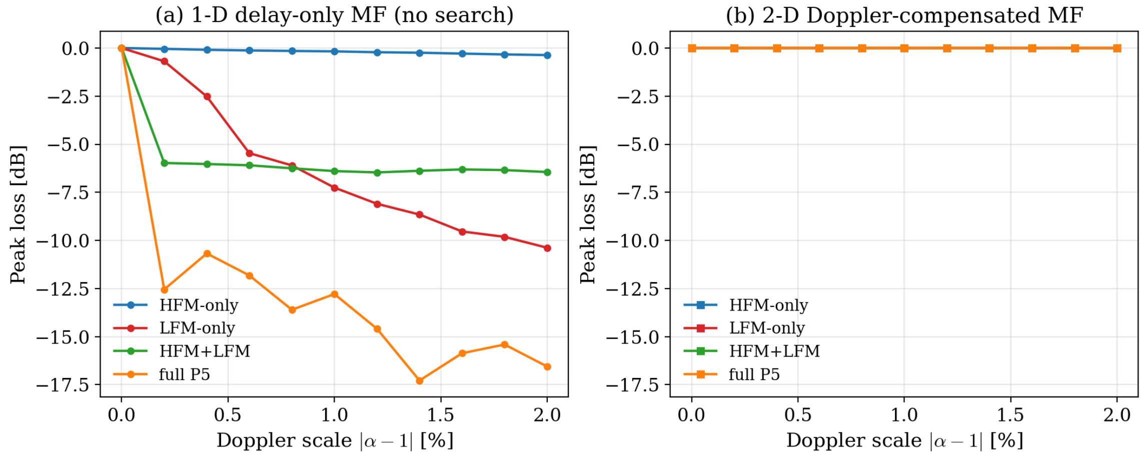

Figure S2: Doppler-invariance ablation: matched-filter peak loss vs. Doppler scale under (a) a 1-D delay-only filter and (b) a 2-D Doppler-compensated filter. Only HFM-only is invariant under (a).

S5.3 CAZAC-layer LPI isolation

The four classical structure-aware detectors (CFD/CS/CEP/SFDM, CFAR-calibrated at $P_{\text{fa}} = 10^{-3}$, total-energy convention) are run on five controls—HFM-only, LFM-only, HFM+LFM (no CAZAC), P5 with a fixed root, and P5 with a per-trial randomized root (Figure S3). The harness reproduces the main sweep: the binding CS detector breaches HFM-only at 23.1 dB, HFM+LFM at 25.3 dB, and P5 at 25.8 dB (matching B1/B5/P5 of the main text, 23.4/25.3/26.0 dB). Adding the CAZAC envelope to HFM+LFM raises the worst-case breach SNR by only +0.4 dB, and root randomization by a further +0.2 dB; CEP and SFDM never breach below 40 dB, CFD only above 34 dB. The CAZAC layer's contribution against the classical detectors is therefore marginal—its value lies in cell identification and the despreading advantage over a root-blind attacker.

S5.4 Sixteen-cell cell identification

A greedy max-min codebook of 16 coprime roots (Figure S4) has a worst-case (max off-diagonal) cross-correlation of only −7.0 dB—far above the 4-cell −16.9 dB and essentially floored by the common-chirp PSL (−7.6 dB), since every cell shares the HFM+LFM chirp. The 16-cell identification accuracy at 10 dB is perfect under balanced-power synchronous (1.00) and asynchronous (1.00) arrival, degrades to 0.80 under joint Doppler+multipath, and collapses under a near-far interferer (the stronger cell wins the template bank): 0.07 at the +6 dB single-scenario point of Figure S4, with the full 0–12 dB sweep of Table S4 showing the collapse already complete at +2 dB (accuracy $\leq$ 0.07). R2 is therefore met as a codebook-cardinality and balanced-power requirement, but near-far-robust 16-cell CID needs per-cell power control or composite-PSL reduction.

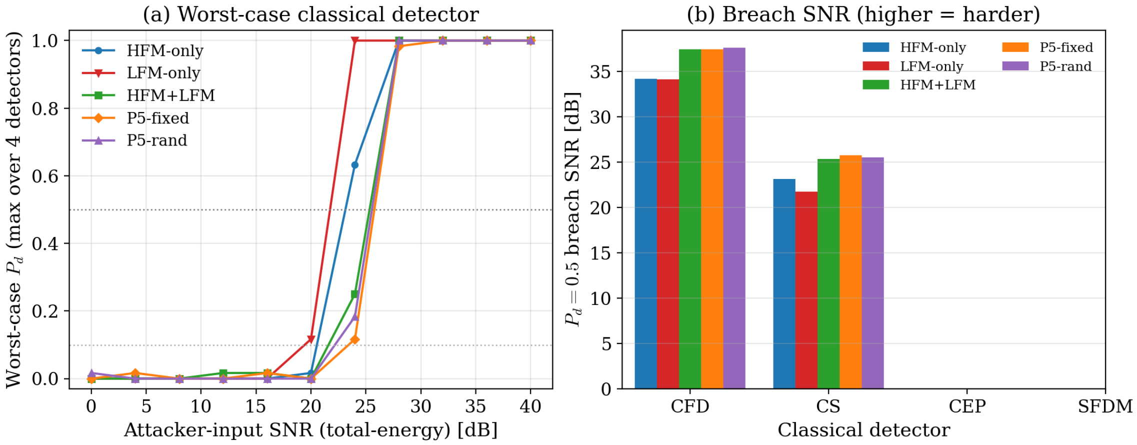

Figure S3: CAZAC-layer LPI ablation (total-energy SNR, $P_{\mathrm{fa}} = 10^{-3}$): (a) worst-case P_d over the four classical detectors; (b) $P_d = 0.5$ breach SNR. Adding CAZAC to HFM+LFM shifts the worst-case breach by only +0.4 dB.

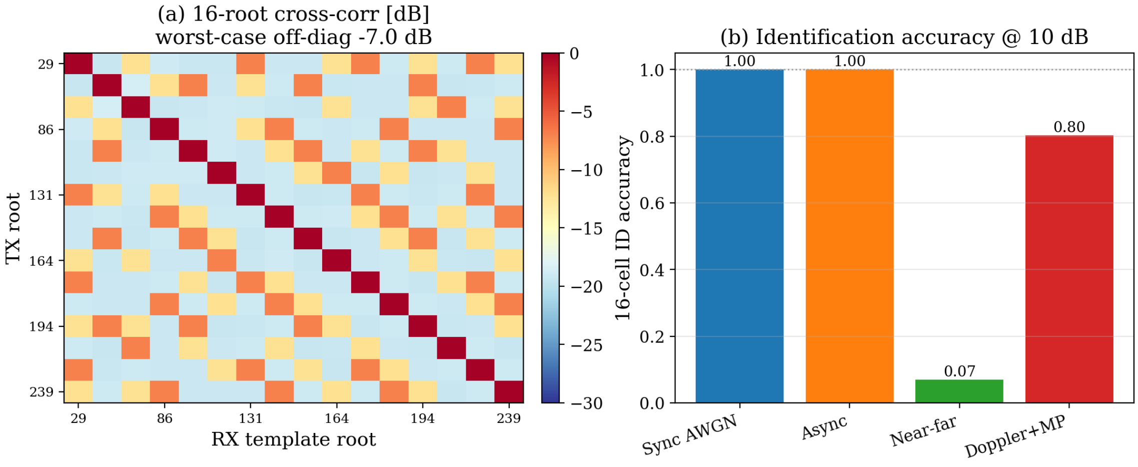

Figure S4: 16-cell CID (10 dB): (a) 16-root cross-correlation matrix (worst-case off-diagonal −7.0 dB); (b) identification accuracy, collapsing to 0.07 under a +6 dB near-far interferer; the full sweep of Table S4 places the collapse threshold at +2 dB.

S5.5 Equal-aperture baseline control

Four composites on the *same* 8–22 kHz aperture (P5, HFM+LFM, HFM+HFM, LFM+LFM) have an identical −3 dB autocorrelation mainlobe (0.062 ms; Figure S5a), so single-target range resolution is set by the spectral aperture, not the chirp type; the LFM layer and CAZAC envelope instead lower the composite PSL (P5 −7.6 vs. HFM+HFM −5.3 dB; Figure S5b), aiding two-target separation. The *real* SA-HFM-family generators confirm this (Table S1): re-allocated from their native 8–16 kHz band to the 8–22 kHz aperture, B5, P3, and P4 all narrow to a 0.052 ms mainlobe—identical to P5—so the native two-target resolution gap closes entirely at equal aperture.

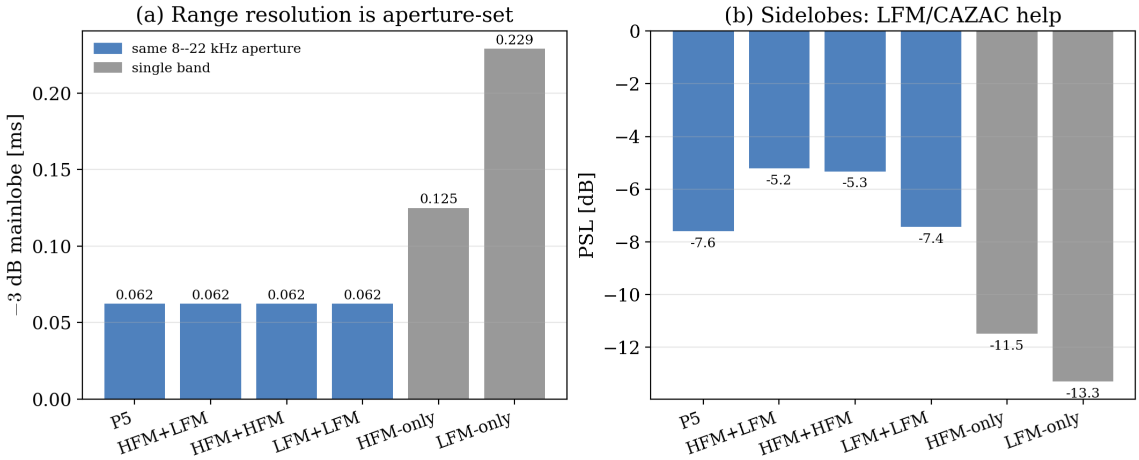

Figure S5: Equal-aperture control: (a) four composites on the same 8–22 kHz aperture share a 0.062 ms mainlobe; (b) the LFM/CAZAC lower the composite PSL.

Table S1: Real baselines: autocorrelation −3 dB mainlobe (range resolution) at native vs. P5's aperture.

	Native (8–16 kHz)		Wide (8–22 kHz)	
Waveform	ml [ms]	ΔR [m]	ml [ms]	ΔR [m]
B1 (HFM)	0.115	0.086	0.073	0.055
B5 (SA-HFM)	0.094	0.070	**0.052**	**0.039**
P3 (HFM–CAZAC)	0.094	0.070	**0.052**	**0.039**
P4 (ISAC-SA-HFM)	0.073	0.055	**0.052**	**0.039**
P5 (native dual-band)	—		**0.052**	**0.039**

S5.6 Template-aware leakage and composite PAPR

Figure S6a contrasts a template-aware attacker holding the exact P5 root (r=29) with a blind attacker using a mismatched root (r=37): the template-aware attacker breaches P_d=0.5 at SNR_{att}=+14.8 dB γ_E versus +29.8 dB for the blind attacker, a +15 dB margin attributable to root randomization (this illustrative stress test uses a reduced N_{H_0}=200, N_{H_1}=80 protocol; the full root-bank/GLRT attacker is reported in S5.11). Figure S6b reports the composite PAPR, which peaks at 3.01 dB near ρ=0.5 and falls to 2.04 dB at the band edges, well below the 6–12 dB typical of OFDM preambles.

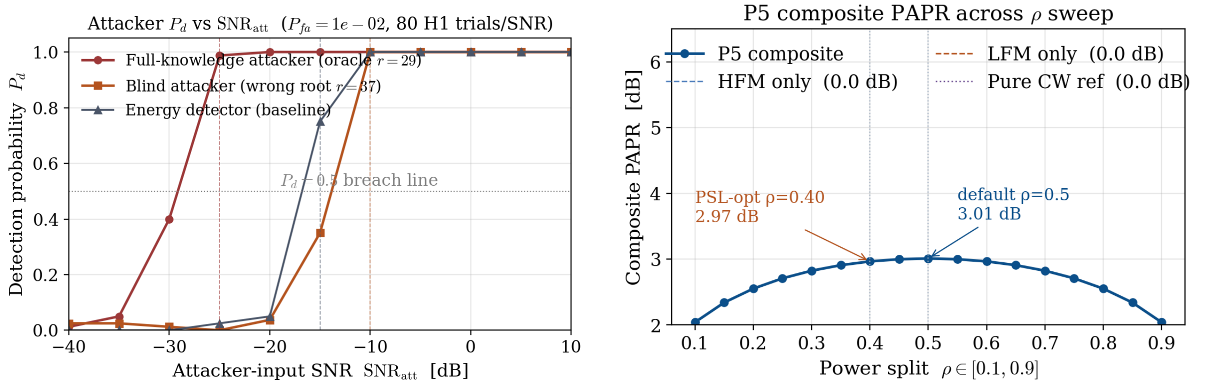

(a) (b)

Figure S6: (a) Attacker P_d vs. $\mathrm{SNR}_{\mathrm{att}}$, full-knowledge ($r$=29) vs. blind ($r$=37). (b) P5 composite PAPR vs. power split ρ.

S5.7 Per-channel L_{eff} distribution

Table S2 resolves the twelve-channel aggregate of the main text (Section 6.3) into per-channel statistics, reporting the *median* L_{eff} (robust to the occasional peak-swap outlier) for B5 and P5 and the per-channel *paired* P5 − B5 advantage with its trial-level 95% confidence interval (50 shared-seed trials). The P5 advantage is positive on *all twelve* channels (median per-channel advantage +0.30 dB), but the per-channel CI excludes zero on only 6/12 environments (three Bellhop and three WATERMARK channels with the strongest multipath), confirming that the effect is *consistent in sign across the evaluated set* while individually significant only where multipath is strong—consistent with the descriptive (not population-inferential) reading adopted in the main text. The WATERMARK rows are statistics-matched surrogates, not measured impulse responses.

Table S2: Per-channel L_{eff} [dB]: medians and the paired P5 − B5 advantage (+ = P5 lower loss).

Channel	B5 med.	P5 med.	P5−B5 mean [95% CI]
Bellhop-ShallowCalm	−0.71	−0.31	+0.48 [+0.13, +0.83]
Bellhop-ShallowRough	−0.48	−0.23	+0.32 [−0.05, +0.70]
Bellhop-DeepWinter	+0.66	+0.73	+0.14 [−0.03, +0.30]
Bellhop-DeepSummer	+0.21	+0.54	+0.31 [+0.11, +0.52]
Bellhop-BayProtected	−1.01	−0.62	+0.49 [+0.13, +0.85]
QS-LowFading	−0.28	−0.28	+0.08 [−0.16, +0.31]
QS-MidFading	−0.49	−0.59	+0.04 [−0.16, +0.23]
QS-HighFading	−0.27	−0.20	+0.05 [−0.30, +0.40]
WMK-NOF1 (surrog.)	−1.19	−1.11	+0.21 [−0.05, +0.48]
WMK-NCS1 (surrog.)	−1.68	−1.15	+0.40 [+0.15, +0.65]
WMK-KAU1 (surrog.)	−1.59	−1.21	+0.29 [+0.01, +0.57]
WMK-KAU2 (surrog.)	−1.70	−1.20	+0.46 [+0.21, +0.71]

S5.8 Multi-seed per-waveform CNN crossings

Table S3 reports the twelve-seed per-waveform trained-CNN $P_d = 0.5$ crossings (main-text § 4.6), restricted to the seeds in which training converged (reached $P_d = 0.5$ by +51.8 dB γ_E). The full rerun fixes the Python, NumPy, PyTorch, and CUDA seeds. Conditional means span 3.44 dB across the ten waveforms (+22.18 to +25.63 dB γ_E). P5 has the *highest* conditional mean, but its rank against several candidates is not resolved by the overlapping uncertainty intervals. The per-waveform convergence rate quantifies the training-seed sensitivity: waveform–seed runs

that fail to converge within the modest budget are excluded from the conditional means, so the reported intervals do not incorporate this right-censoring.

Table S3: Per-waveform trained-CNN $P_d = 0.5$ crossing [dB], deterministic 12-seed rerun, conditional on convergence. Entries are conditional mean [95% t interval]. Total-energy γ_E; the per-sample training grid maps to γ_E via $+10\log_{10} N_s = +39.8$ dB.

wf	conv.	mean [95% CI]	wf	conv.	mean [95% CI]
B1	9/12	+22.46 [+21.69, +23.23]	P1	9/12	+23.46 [+22.17, +24.74]
B2	11/12	+22.18 [+21.77, +22.59]	P2	12/12	+23.19 [+22.77, +23.61]
B3	11/12	+22.82 [+22.45, +23.19]	P3	11/12	+23.26 [+22.09, +24.44]
B4	8/12	+23.74 [+22.63, +24.86]	P4	11/12	+22.97 [+22.63, +23.31]
B5	10/12	+23.04 [+22.51, +23.57]	**P5**	11/12	+**25.63** [+24.35, +26.91]

S5.9 Sixteen-cell near–far sweep

Table S4 sweeps the near–far interferer ratio for the greedy 16-root codebook (main-text § 5.10, E-03; 100 trials/cell, asynchronous arrival, 10 dB SNR). With an equal-power co-cell interferer (0 dB) the identification accuracy is 0.69; a $\geq +2$ dB interferer collapses it to ≤ 0.07—essentially the common-chirp PSL floor (-7.6 dB), since every cell shares the HFM+LFM chirp. This confirms that the operational CID facet (R2b) is met only near balanced power and that near–far-robust 16-cell CID requires per-cell power control or composite-PSL reduction.

Table S4: 16-cell CID accuracy vs. near–far interferer ratio.

Near–far [dB]	0	2	4	6	8	10	12
ID accuracy	0.69	0.07	0.06	0.05	0.06	0.07	0.07

S5.10 Equal-aperture sidelobe level of the real baselines

Complementing the mainlobe closure of Table S1, Table S5 reports the autocorrelation PSL and -3 dB mainlobe of the *real* B5/P3/P4 generators at their native (8–16 kHz) versus the wide (8–22 kHz) aperture. At the wide aperture all three match P5's 0.052 ms mainlobe *and* the SA-HFM family attains a *lower* composite PSL than P5 (B5 -15.6 dB, P4 -14.0 dB vs. P5 -7.6 dB). P5 is therefore not individually superior in single-target resolution or sidelobe level once the aperture is equalized; its contribution is the *integration* of R1 Doppler invariance, R4 ranging, R2 cell identification, and D5 anti-detection in one matched-filter-recoverable, constant-modulus-envelope preamble, not a single-metric optimum.

Table S5: Real baseline autocorrelation PSL and -3 dB mainlobe at native vs. wide (8–22 kHz) aperture.

	native 8–16		wide 8–22	
Waveform	PSL [dB]	ml [ms]	PSL [dB]	ml [ms]
B5 (SA-HFM)	−9.95	0.094	**−15.57**	0.052
P3 (HFM–CAZAC)	−6.76	0.094	−8.00	0.052
P4 (ISAC-SA-HFM)	−9.20	0.073	−14.01	0.052
P5 (native dual-band)	—		−7.60	0.052

S5.11 Root-bank / GLRT attacker

Table S6 reports the breach $\mathrm{SNR}_{\mathrm{att}}$ ($P_d = 0.5$) of four attacker models against P5 (default root $r = 29$): the full-knowledge oracle (exact template), the 128-root GLRT bank (maximum statistic over *all* $\phi(255) = 128$ coprime-root templates, multiple-comparison CFAR on the same bank), the single-wrong-root blind attacker, and the energy detector ($N_{H_0} = 600$, $N_{H_1} = 200$, $P_{\mathrm{fa}} = 10^{-3}$). The bank nearly matches the oracle (within 1.9 dB) and is 15.6 dB stronger than the single-wrong-root attacker, confirming that root randomization gives little protection once the adversary searches the (public) root set—hence the main-text D5/LPI results are scoped to blind attackers without a root-template bank.

Table S6: Attacker breach $\mathrm{SNR}_{\mathrm{att}}$ at $P_d = 0.5$ against P5 (total-energy γ_E; per-sample grid mapped via $+10\log_{10} N_s = +39.8$ dB).

Attacker model	Breach $\mathrm{SNR}_{\mathrm{att}}$ [dB]
Full-knowledge (exact template, oracle)	+10.0
**128-root GLRT bank**	**+11.9**
Energy detector	+24.4
Single wrong-root (blind)	+27.5

S5.12 Range-error outlier statistics

Table S7 reports range-error outlier statistics for $\mathrm{P5}^{\mathrm{def}}$ at 10 dB (1000 trials/point); the same catastrophic peak-swap mechanism underlies the elevated mean RMSE of the 0 dB delay-spread sweep (Figure S7, main-text E-02, § 5.10). The median and the 90/95/99th-percentile range errors stay at the floor (≈ 0); the RMSE is inflated *entirely* by a small catastrophic peak-swap rate (0.2–0.8%; $|\hat{R} - R| > 0.3$ m, the matched filter locking onto a secondary arrival). Typical-case ranging is therefore robust, and the gross-error probability $P(|\hat{R} - R| > 1\,\mathrm{m}) \leq 0.8\%$ quantifies the reliability tail that the mean RMSE alone obscures.

Table S7: $\mathrm{P5}^{\mathrm{def}}$ range-error outlier statistics vs. delay spread (10 dB, 1000 trials/pt).

τ_{rms} [ms]	median [m]	RMSE [m]	peak-swap rate	$P(\lvert\cdot\rvert > 1\,\mathrm{m})$
5	0.00	0.00	0.000	0.000
10	0.00	0.45	0.008	0.008
20	0.00	0.00	0.000	0.000
30	0.00	1.13	0.002	0.002
50	0.00	1.53	0.002	0.002

S5.13 Aggregate-score interpretation

The aggregate column of main-text Table 11 is reproducible as a deterministic application of the stated scoring rule to the displayed point estimates. We also examined whether joint sampling uncertainty could be estimated for this aggregate. The differentiating σ_v, σ_τ, and CID inputs are available as rounded per-waveform summaries, but the retained data do not include shared trial-level error or correctness observations for all eleven rows. A valid joint paired interval is therefore not supported.

Table S8 reports the deterministic point scores without a significance claim for their ordering. The interpretation is descriptive and weight-dependent; the per-column metrics in main-text Table 11 remain the primary evidence. The scoring script and machine-readable summaries are available from the corresponding author on request.

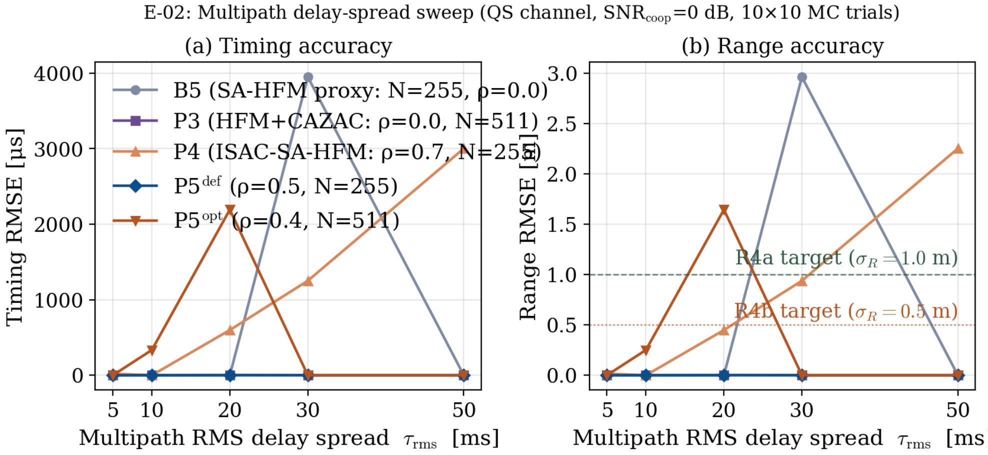

Figure S7: E-02: timing (a) and range (b) RMSE vs. delay spread τ_{rms} on a Qarabaqi–Stojanovic channel ($\text{SNR}_{\text{coop}} = 0$ dB). The elevated mean RMSE at large τ_{rms} is driven by the rare peak-swap outliers quantified in Table S7, not a smooth degradation.

Table S8: Aggregate score summary. Values are deterministic point scores; no joint paired interval is reported because shared trial-level observations are unavailable for all candidates.

Waveform	Point score
B1 (HFM single)	0.6450
B2 (dual HFM)	0.6550
B3 (LFM+ZC)	0.6975
B4 (sup-LFM)	0.5950
B5 (SA-HFM)	0.8825
P1 (ext SA-HFM)	0.8825
P2 (phase-coded SA-HFM)	0.8700
P3 (HFM+CAZAC)	0.8640
P4 (ISAC-SA-HFM)	0.8875
P5^{def}	0.9155
P5^{opt}	0.9005

S5.14 Stronger learned attacker: 2-D spectrogram ResNet-18

To address the concern that a compact 1-D CNN under-represents the learned-attacker threat, a ResNet-18 (11,170,753 parameters, trained from scratch, single-channel input, 1-logit head) was trained on log-magnitude STFT spectrograms (256×147; $n_{\text{perseg}} = 256$, hop 64, per-image standardized) of the same energy-normalized windows used by the per-waveform 1-D CNN study (S5.8): H1 = waveform with random arrival shift and global phase + AWGN, H0 = AWGN, CFAR threshold at $P_{\text{fa}} = 10^{-3}$ on an $N_{H_0} = 8000$ score pool, $N_{H_1} = 400$ per SNR point (a reduced-statistics protocol relative to the main D5 sweep's $10^4/10^3$; crossings are interpolated on a 2 dB grid).

Protocol asymmetry (deliberate). The 1-D CNN reference is trained on its native fixed 4000-example set; the ResNet-18 is trained *online* (fresh 3000 windows per epoch $\times$ 14 epochs, training SNR uniform over $[+9.8, +49.8]$ dB γ_E), because an 11M-parameter model memorizes a fixed set of that size (a matched-protocol ResNet run generalizes *worse* than the 1-D CNN and is reported in the archived JSON only as a negative control). The online budget models a non-cooperative attacker that can synthesize unlimited training signals from the known waveform family. A fairness control retrains the 1-D CNN with the identical online budget on P5: it breaches at +22.9 dB γ_E. Relative to the deterministic-rerun fixed-set 1-D CNN conditional mean, the 10-seed ResNet mean is 4.39 dB lower; the online 1-D control associates about 2.7 dB of this cross-protocol gap with the data-budget change and leaves about 1.7 dB for architecture and run-to-run differences. This is a descriptive comparison, not a factorial causal decomposition.

Table S9 reports per-waveform $P_d = 0.5$ crossings (ten seeds, mean $\pm$ SD, γ_E axis via the S5.8 convention $+10\log_{10} N_s = +39.8$ dB). Figure S8 compares the P_d curve for P5 and per-waveform ResNet crossings. All ten means lie within a 3.15 dB band. Under this evaluated single-sensor exact-template protocol, P5 has the highest mean crossing; the paired P5–P1 difference is +0.49 dB (95% CI $[+0.33, +0.65]$ dB), and the paired P5–P4 difference is +0.56 dB ($[+0.34, +0.77]$ dB). A separate direct evaluation at 0 dB γ_E used 2000 fresh P5 H1 windows per seed and gave mean $\hat{P}_d = 0.0014$ (seed-level 95% CI $[0.00075, 0.00205]$), consistent with the P_{fa} floor. The 0-dB point is outside the training-SNR range and is a direct protocol-specific check rather than an interpolated curve value. The analysis scripts (`spectrogram_resnet.py`, `run_spectrogram_resnet.py`) and per-seed JSONs are included in the revision analysis package and are available from the corresponding author on request.

Table S9: Per-waveform $P_d = 0.5$ crossing [dB γ_E]: 2-D spectrogram ResNet-18 (online, 10 seeds, mean±SD) vs. the per-waveform 1-D CNN reference (fixed set, deterministic 12-seed rerun, S5.8 conditional means). $\Delta < 0$ = the ResNet breaches earlier (stronger attacker).

wf	ResNet-18	1-D CNN	Δ	wf	ResNet-18	1-D CNN	Δ
B1	$+18.09 \pm 0.15$	+22.46	−4.37	P1	$+20.75 \pm 0.15$	+23.46	−2.71
B2	$+18.88 \pm 0.16$	+22.18	−3.30	P2	$+20.61 \pm 0.19$	+23.19	−2.58
B3	$+19.58 \pm 0.24$	+22.82	−3.24	P3	$+19.68 \pm 0.18$	+23.26	−3.58
B4	$+19.13 \pm 0.13$	+23.74	−4.61	P4	$+20.68 \pm 0.11$	+22.97	−2.29
B5	$+20.66 \pm 0.11$	+23.04	−2.38	**P5**	$\mathbf{+21.24 \pm 0.25}$	**+25.63**	**−4.39**

S5.15 Measured-channel replay: full statistics and confound verification

Main-text § 6.5 replays band-scaled waveform variants through the measured NOF1/NCS1 impulse responses of the FFI WATERMARK V1 package (all 60 soundings per track; noise-free replay; 8460/14,340 packets per waveform for NOF1/NCS1). All four waveforms share one linear band map from the native 4–24 kHz design space into the measured 10–18 kHz band; P5's CAZAC length is reduced to $N = 127$, the largest value keeping $\geq 95\%$ of energy in-band

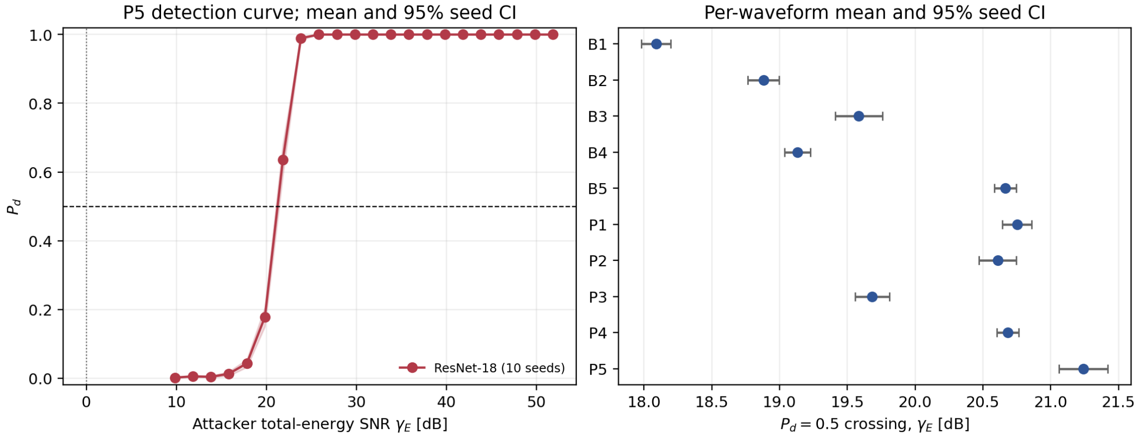

Figure S8: Ten-seed spectrogram ResNet-18 evaluation. Left: mean P_d against P5 versus total-energy attacker SNR, with a 95% confidence band over training seeds. Right: mean per-waveform $P_d = 0.5$ crossings with 95% seed-level confidence intervals; lower crossing denotes a stronger attacker. P5 has the highest mean under this evaluated single-sensor exact-template protocol.

(in-band fractions: B5 99.96%, P4 99.91%, $P5^{def}$ 95.5%, $P5^{opt}$ 95.8%). The same sounding and packet segment is paired across waveforms. Because packets within a sounding are correlated, the primary analysis averages within sounding and uses the 60 sounding means as independent units. Table S10 reports those all-sounding results and separately labels the earlier first-eight-sounding mechanistic controls.

The primary 20,000-resample paired cluster bootstrap (Figure S9) gives $P5^{def}$–B5 differences of -0.816 dB $[-1.021, -0.621]$ on NOF1 and -0.950 dB $[-0.977, -0.923]$ on NCS1, and $P5^{def}$–P4 differences of -0.707 dB $[-0.887, -0.535]$ and -0.839 dB $[-0.863, -0.815]$, respectively (95% CIs; seed 20260720). A paired hierarchical bootstrap that resamples soundings and then packets gives closely matching $P5^{def}$–B5 intervals $[-1.023, -0.622]$ and $[-0.987, -0.913]$ dB. Agreement between the two schemes shows that the adverse conclusion is not an artifact of treating the 91,200 packet records as independent.

Exploratory control summary. The following V1–V4 analyses use only the first eight consecutive soundings of each track and therefore do not establish the cause of the all-60-sounding result. (V1) The native-N variant ($N = 255$, in-band 91.0%) performs 0.39–0.46 dB *worse* than $N = 127$ on both tracks, so the length reduction favored P5 and is not a confound; lower chip rates are preferable in rapidly varying channels. (V2) The no-CAZAC control (HFM+LFM, same scaled bands) recovers essentially to B5's level (-0.29 dB on NOF1; $+0.07$ dB, CI including zero, on NCS1) and exceeds $P5^{def}$ by $+0.68$ / $+0.96$ dB, suggesting a CAZAC chip-modulation coherence cost under channel time variability. (V3) An equal-bandwidth LFM probe pair (band-center vs. the band-edge position of the scaled P5 LFM layer) bounds the edge-placement contribution at -0.55 dB on NCS1 and statistically zero on NOF1. (V4) The average measured transfer functions (not shown) show a -2.4 dB upper-band (15–18 kHz) rolloff on NOF1 and none on NCS1, consistent with V3 in that eight-sounding subset. Scripts (`exp_er4_watermark_replay.m`, `exp_er4_verification.m`) and raw per-packet losses are included in the revision analysis package and are available from the corresponding author on request.

Table S10: Measured-channel replay. Primary rows: mean ± SD of L_{eff} [dB] across 60 sounding means. Rows below the rule are exploratory first-eight-sounding controls and are not full-run estimates.

Signal	NOF1	NCS1
B5 (SA-HFM)	-4.389 ± 1.382	-7.231 ± 0.313
P4 (ISAC-SA-HFM)	-4.498 ± 1.347	-7.343 ± 0.324
$\mathrm{P5}^{\mathrm{def}}$ (N=127)	-5.205 ± 1.286	-8.181 ± 0.329
$\mathrm{P5}^{\mathrm{opt}}$ (N=127)	-5.081 ± 1.278	-8.100 ± 0.316
V1: P5 native N=255	−5.26	−8.57
V2: HFM+LFM, no CAZAC	−4.12	−7.23
V3: LFM probe, band-center	−2.86	−5.32
V3: LFM probe, band-edge	−2.94	−5.88

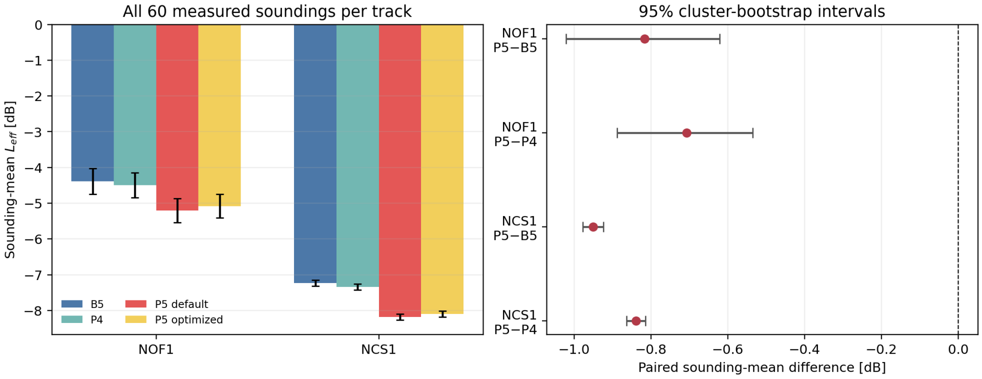

Figure S9: All-60-sounding measured-channel replay. Left: mean L_{eff} across sounding means with 95% t intervals. Right: paired $\mathrm{P5}^{\mathrm{def}}$ differences with 95% sounding-cluster bootstrap intervals (20,000 resamples).

References

[1] Van Trees, H.L. *Detection, Estimation, and Modulation Theory, Part III*; Wiley: New York, NY, USA, 2001.
